# Diffraction-Limited Molecular Cluster Quantification with Bayesian Nonparametrics

**DOI:** 10.1038/s43588-022-00197-1

**Published:** 2022-02-28

**Authors:** J. Shepard Bryan, Ioannis Sgouralis, Steve Pressé

**Affiliations:** 1 Center for Biological Physics, Arizona State University; 2 Department of Mathematics, University of Tennessee Knoxville; 3 School of Molecular Sciences, Arizona State University

**Keywords:** photobleaching, stochastic dynamics, superresolution, biophysics, molecular biology, data analysis, Bayesian nonparametrics

## Abstract

Life’s fundamental processes involve multiple molecules operating in close proximity within cells. To probe the composition and kinetics of molecular clusters confined within small (diffraction-limited) regions, experiments often report on the total fluorescence intensity simultaneously emitted from labeled molecules confined to such regions. Methods exist to enumerate total fluorophore numbers (e.g., step counting by photobleaching). However, methods aimed at step counting by photobleaching cannot treat photophysical dynamics in counting nor learn their associated kinetic rates. Here we propose a method to simultaneously enumerate fluorophores and determine their individual photophysical state trajectories. As the number of active (fluorescent) molecules at any given time is unknown, we rely on Bayesian nonparametrics and use specialized Monte Carlo algorithms to derive our estimates. Our formulation is benchmarked on synthetic and real data sets. While our focus here is on photophysical dynamics (in which labels transition between active and inactive states), such dynamics can also serve as a proxy for other types of dynamics such as assembly and disassembly kinetics of clusters. Similarly, while we focus on the case where all labels are initially fluorescent, other regimes, more appropriate to photoactivated localization microscopy, where fluorophores are instantiated in a non-fluorescent state, fall within the scope of the framework. As such, we provide a complete and versatile framework for the interpretation of complex time traces arising from the simultaneous activity of up to 100 fluorophores.

## 1 Introduction

Fluorescently labeled molecules, such as labeled proteins, are often used to create contrast between a cell’s background and the labeled molecular species of interest [1, 2, 3]. As biological processes unfold within cellular environments, these labeled molecular species may aggregate into clusters giving rise to the appearance of bright spots in fluorescence microscopy [4, 5, 6, 7]. Assessing the composition of these clusters on the basis of the spot’s variable brightness is a key step toward unraveling the role of molecular clusters [4, 5, 8, 9, 10, 6, 7].

**Figure 1:**
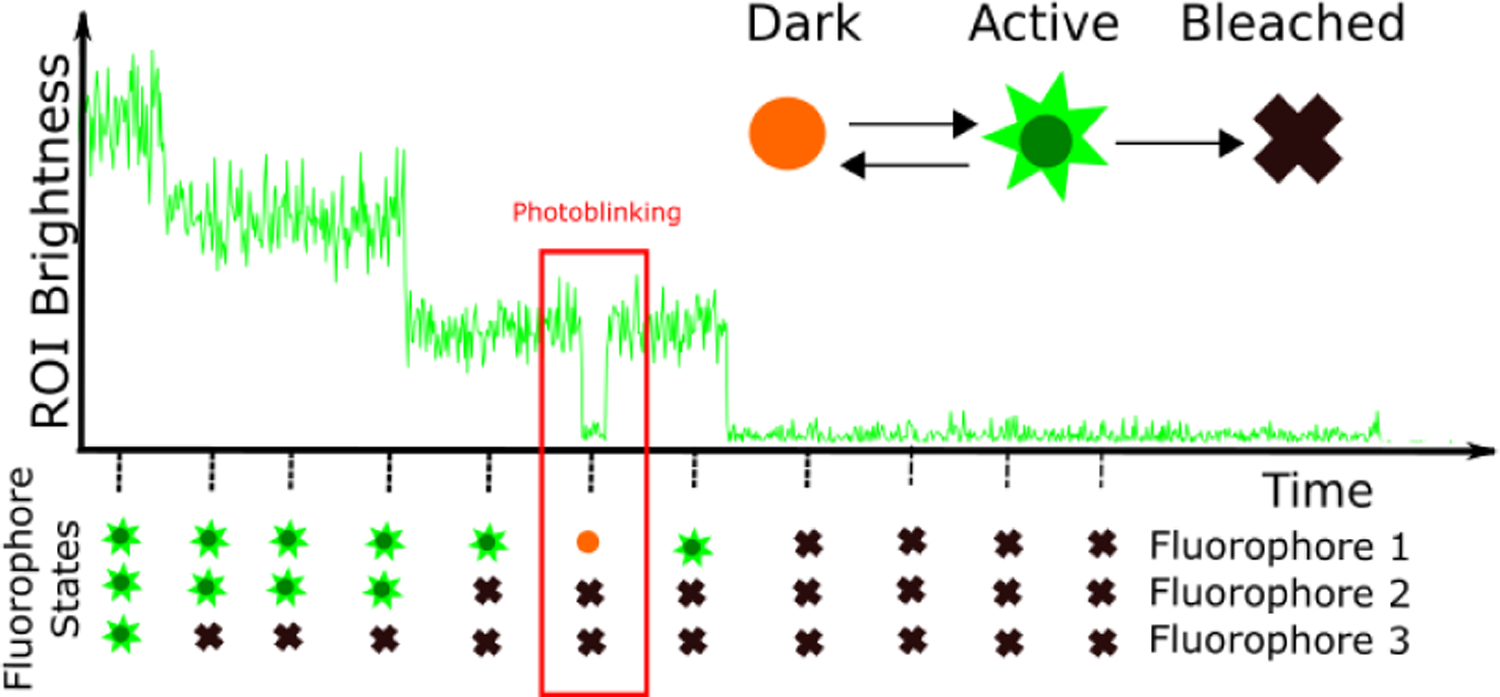
Summary of the problem. Each fluorophore attains one of three types of states: dark, active (bright), or photobleached. Only fluorophores in the active state emit photons. Each fluorophore transitions between these states as indicated by the arrows. The brightness over time of an ROI reflects the states of all fluorophores within the ROI. Our goal is to estimate the number of fluorophores in each ROI, as well as the photo-states of the fluorophores at each time level.

Directly enumerating fluorophores and tracking their photophysical dynamics by discriminating between them on the basis of their physical location [11] is often impossible as typically an entire bright spot lies below the diffraction limit [2, 3]. Furthermore, fluorescence ruler methods, which enumerate fluorophores across time by comparing the brightness of a region of interest (ROI) to the brightness of a known calibration standard, are unreliable when the number of fluorophores is large on account of the inherent uncertainty introduced by photon shot noise which increases with growing fluorophore numbers [12, 13]. Other sources of uncertainty, beyond shot noise, include camera or detector noise and the rapid rise and fall of fluorescence intensity of the spot [4, 5]. The latter can arise on account of photophysical activity of the individual fluorophore labels as they cycle between fluorescently emitting or active (i.e., bright) and non-emitting or inactive (i.e., dark or photobleached) states [14] or it can arise due to assembly and disassembly of a cluster as individual constituents bind and unbind. For the purposes of this manuscript we will focus on brightness steps as being caused exclusively by photophysical dynamics and postpone further mention of (dis)assembly to the Discussion. A cartoon depicting this process is shown in figure 1.

Traditionally, *Photobleaching step analysis* (PBSA) methods were developed to enumerate the number of fluorophores within a spot [12, 13, 15, 16, 17, 18, 19, 20, 21, 22, 23, 24, 25]. Such methods proceed in many ways; for example, by exploiting hidden Markov models [23, 25], data filtering to identify steps [17], statistical measures to identify expected violations of statistics characteristic of steps [21, 18, 20, 19], or neural nets [22]. In a recently submitted manuscript, additional ways of automating and improving upon PBSA methods listed above have also been explored [26]. Yet all PBSA are limited to clusters where fluorophores irreversibly inactivate one at a time until they are all photobleached giving rise to step-like transitions between brightness levels [27, 15, 2, 28, 29, 30, 31, 32, 33, 34, 35, 36, 37, 38, 39].

Our goal is to present a general framework that can simultaneously count and determine the photo-trajectories of fluorophores within a diffraction-limited ROI while taking into account photophysical artifacts such as blinking. To achieve this: 1) we exploit a realistic generative model that includes accurate photophysics, learns photophysical parameters, and can treat detailed camera models; 2) we relax the requirement that fluorophores all initialize from the bright state; and 3) we provide full Bayesian analysis providing not only point estimates, but also uncertainties over all unknown parameters. We show that the novel aspects of our method allow us to count upwards of 100 fluorophores in a single ROI. By virtue of the generality of our framework, we have the ability to treat other camera models or re-interpret the brightness step transitions as cluster assembly and disassembly kinetics. To illustrate our method, we use data in which a spot is illuminated with bright light and whose active fluorophores undergo photophysical transitions between bright and dark states before eventually photobleaching. As the number of fluorophores at any given time is unknown, we exploit tools within Bayesian nonparametrics [40, 41], in particular the Beta-Bernoulli process (BBP) [42, 43] never previously exploited in assessing the kinetics and composition of molecular clusters.

## 2 Methods

Here we set up: 1) the forward model, i.e., a model describing the stochastic dynamics of a large collection of molecules as well as other contributions to the data; and 2) discuss the inference strategy required to learn the number of fluorophores and their photophysical trajectories from the data. In particular, we show how we estimate the state (for simplicity we refer to photo-states as states) of each fluorophore at each time, the transition probabilities between fluorophore states, the probability that a fluorophore starts bright, the fluorophore mean brightness, and the background mean brightness. As with all methods within the Bayesian paradigm, whether parametric or nonparametric, we provide not only a point estimate for the maximum *a posteriori* (MAP) value of each variable, but also achieve full posterior inference with credible intervals.

### 2.1 Forward model

The forward model describes how the data are generated. We start with *R* diffraction-limited ROIs, indexed by *r* = 1, …, *R*. Each ROI has *K*_*r*_ fluorophores indexed by *m* = 1, …, *K*_*r*_. We record the brightness (measured in ADUs) of each ROI for *N* successive time levels, indexed by *n* = 1, …, *N*. The brightness of the ROI *r*, at time level *n* is denoted $w_{n}^{r}$ and is conditioned on the states of the fluorophores within the ROI at that time. The state of the *k*th fluorophore in ROI *r* at time level *n* is labeled $S_{n}^{k,r}$. For simplicity, at each time level, we let $s_{n}^{k,r}$ be in one of three states: dark, *σ*_*D*_, active, *σ*_*A*_, and photobleached, *σ*_*B*_. We tackle the obstacle of multiple bright states in the supplement (supplement 5.5).

At the first time level, each fluorophore in each ROI, starts either active or dark with probabilities given by ***π***_0_ which is an array with two elements: the probability of a fluorophore starting bright, *π*_0*A*_ and the probability of a fluorophore starting dark, *π*_0*D*_. At each following time level, *n*, the state of each fluorophore is conditioned on the previous state of the fluorophore according to ***π***, the transition probability matrix. Each element, *π*_*ij*_, of the matrix represents the probability that a fluorophore will be in state *σ*_*j*_ given that it was previously in state *σ*_*i*_ (supplement 5.2). These transitions include “dark to dark”, “dark to bright”, “bright to bright”, “bright to dark”, “bright to photobleached”, or “photobleached to photobleached” transitions (figure 1).

Our kinetic scheme is mapped here,

(1) $$s_{1}^{k,r}\sim \;\text{Categorical}\;\left(\pi_{0}\right)$$

(2) $$s_{n}^{k,r}|s_{n-1}^{k,r}\sim \;\text{Categorical}\;\left(\pi_{s_{n-1}^{k,r}}\right)$$

Where $S_{n}^{k,r}$ is the state of fluorophore *k* in ROI *r* at time level *n*, ~ means “is sampled from”, | means “given” or “conditioned on”, **Categorical** (*x*) means “the categorical distribution with probability mass *x*”, and $\pi_{s_{n-1}^{k,r}}$ means “the row of ***π*** corresponding to the state of $s_{n-1}^{k,r}”$. The support for these categorical distributions is understood to be the set of possible states of the fluorophores, {*σ*_*D*_, *σ*_*A*_, *σ*_*B*_} meaning that for all *n*, *k*, and *r*, $s_{n}^{k,r}=\sigma_{D}$, $s_{n}^{k,r}=\sigma_{A}$, or $s_{n}^{k,r}=\sigma_{B}$.

At each time level, the fluorophores in each ROI give rise to the mean brightness of the ROI at the time level, $\mu_{n}^{r}$. The mean brightness, $\mu_{n}^{r}$ is the expected number of photons for the time level (calculated as the time step multiplied by mean photons emitted per unit time for the time level). We can decompose it into the sum of the mean background brightness of the ROI, $\mu_{B}^{r}$, and the mean fluorophore brightness, *μ*_*A*_, multiplied by the number of active fluorophores in the ROI,

(3) $$\mu_{n}^{r}=\mu_{B}^{r}+\overset{K_{r}}{\underset{k=1}{\sum }}\mu_{s_{n}^{k,r}}$$

Where $\mu_{s_{n}^{k,r}}$ means “the brightness of the state corresponding to $S_{n}^{k,r}”$, as in, if $S_{n}^{k,r}=\sigma_{A}$ then $\mu_{s_{n}^{k,r}}=\mu_{A}$ or if $S_{n}^{k,r}=\sigma_{B}$ then $\mu_{s_{n}^{k,r}}=0$. We note that $\sum_{k=1}^{Kr}\mu_{s_{n}^{k,r}}$ simply counts how many fluorophores are in the active state in the ROI at the time level of interest.

For data obtained with an EMCCD camera the brightness measured, $w_{n}^{r}$, is conditioned on the mean brightness and the gain, *G*, through a gamma distribution [44]

(4) $$w_{n}^{r}|\mu_{n}^{r}\sim \text{Gamma}\left(\mu_{n}^{r}/2,2G\right).$$

This model takes into account both shot and the readout noise [45]. Substituting Eq. (3) into Eq. (4) we find

(5) $$W_{n}^{r}|S_{n}^{1:K_{r},r},\mu_{A},\mu_{B}^{r}\sim \text{Gamma}\left(\frac{1}{2}\left(\mu_{B}^{r}+\overset{K}{\underset{k=1}{\sum }}\mu_{s_{n}^{k,r}}\right),2G\right).$$

With this model, the mean expected readout is $\mu_{n}^{r}G$ ADUs (units of camera readout) with a standard deviation of $\sqrt{2\mu_{n}^{r}G^{2}}$ ADUs. Thus our model’s noise scales with the brightness with an excess noise factor of 2 that is characteristic of EMCCDs [46, 44]

This scheme, where many fluorophores give rise to a single measurement (the brightness at a time level), takes the form of a factorial hidden Markov model [47, 48].

### 2.2 Inverse formulation

**Figure 2:**
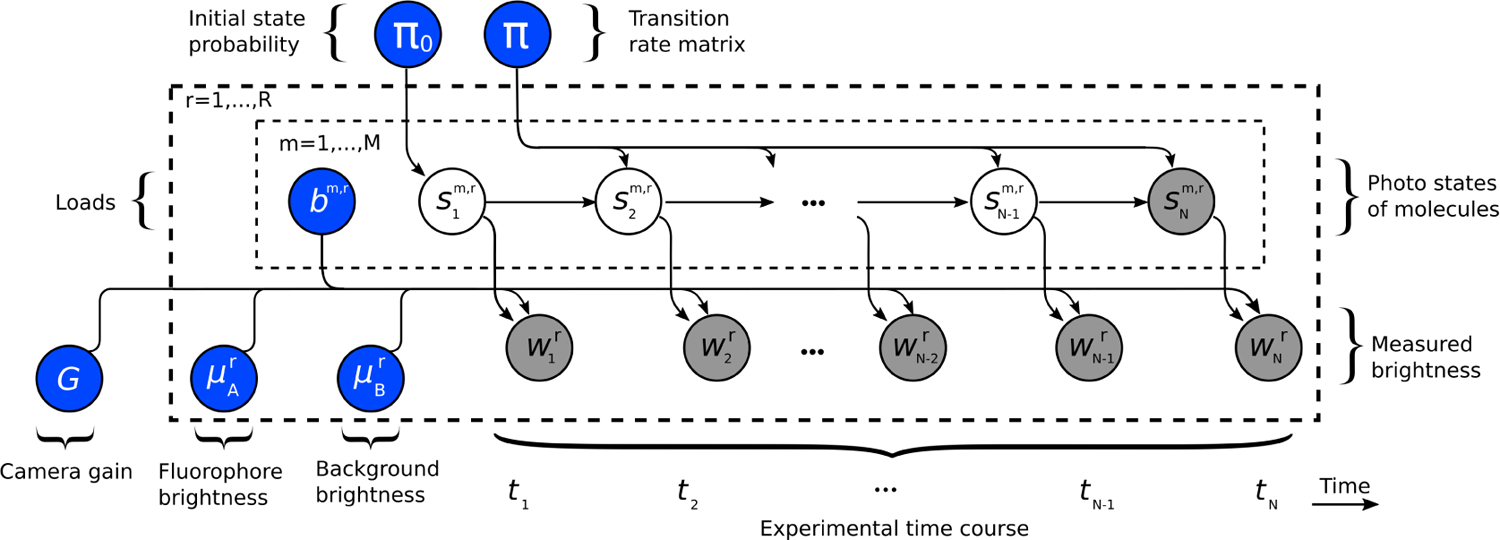
The graphical representation of the inverse model. Nodes (circles) in the graphical model represent random variables. Blue nodes are the random variables we infer. White nodes are auxiliary random variables (latent variables). Grey nodes are observations (data). The arrow between nodes indicates conditional dependence, meaning that if *x* is conditioned on *y* then we would draw an arrow going from node *y* to node *x*. The plates (dashed boxes) indicate that random variables within plates repeat over the index appearing at the top left of the plate. For example, the $\mu_{B}^{r}$ node is within the outer plate with index *r* implying an $\mu_{B}^{r}$ associated to each ROI (indexed *r*).

We now develop the inverse formulation needed to estimate parameters from the data given a known number of ROIs, *R*, and associated time trace lengths, *N*. Following the Bayesian paradigm, we place prior distributions on all parameters whose posterior distribution we wish to determine. A graphical representation of our inverse model is shown in figure 2. Our choice of priors for transition rates and brightness parameters is straightforward and can be found in the SI (See supplemental section 5.1). However, our prior on the number of fluorophores is less straightforward as it requires a Bayesian nonparametric formulation that we outline below.

As we cannot set a prior on the number of fluorophores in each ROI, *K*_*r*_, we invoke Bayesian nonparametrics in our analysis. Briefly, we implement this using a nonparametric weak limit [49, 50]. That is, we assume an exceedingly large number of model fluorophores in the ROI, *K* ≫ *K*_*r*_, indexing each fluorophore with *k* = 1, …, *K*. We then assign each model fluorophore a load variable *b*^*k*,*r*^. If the load is on, *b*^*k*,*r*^ = 1, we say that the fluorophore contributes to the ROI’s brightness. If the load is off, *b*^*k*,*r*^ = 0, then the fluorophore is a virtual fluorophore which does not contribute to the brightness. Thus by summing the loads over all model fluorophores, we obtain the number of fluorophores located within the ROI. A load, *b*^*k*,*r*^, is a random variable sampled from the Bernoulli distribution with hyperparameter *γ*

(6) $$b^{k,r}\sim \text{Bernoulli}\left(\frac{\gamma }{K+\gamma -1}\right).$$

This probability mass is motivated by the Beta-Bernoulli process [42, 43] further discussed in supplement 5.4. In particular, as *K* becomes large, formally as *K* → ∞, the probability distribution converges to a distribution in which an infinite number of model fluorophores are considered [49, 50]. This choice of prior allows for inference independent on our choice for *K* provided a sufficiently large *K* (exceeding any reasonable number of fluorophores) is set; see supplement 5.10).

In analogy to equations (1)–(5), states are sampled just as we did in the forward model, except that each measurement, $w_{n}^{r}$, is now conditioned on the loads

(7) $$s_{1}^{k,r}|\pi_{0}\sim \;\text{Categorical}\;\left(\pi_{0}\right)$$

(8) $$S_{n}^{k,r}|S_{n-1}^{k,r},\pi \sim \;\text{Categorical}\;\left(\pi_{s_{n-1}^{k,r}}\right)$$

(9) $$w_{n}^{r}|s_{n}^{1:K,r},b^{1:K,r},\mu_{A},\mu_{B}^{r}\sim \text{Gamma}\left(\frac{1}{2}\left(\mu_{B}^{r}+\overset{K}{\underset{k=1}{\sum }}b^{k,r}\mu_{s_{n}^{k,r}}\right),2G\right).$$

Here $\sum_{k=1}^{K}b^{k,r}\mu_{s_{n}^{k,r}}$ enumerates the number of fluorophores simultaneously active (i.e., loads in the active state in the ROI at time level *n*).

Lastly, if experiments are carried out long enough, all fluorophores eventually irreversibly photobleach. As such, we have knowledge of the final states of the fluorophores. Put differently, the fluorophore states at the last time level are fixed at

(10) $$s_{N}^{k,r}=\sigma_{B}.$$

As such $S_{N}^{k,r}$ is shaded in grey in figure 2.

Together, these equations allow us to construct the high dimensional posterior over the collection of random variables ($S_{1:N}^{1:K,1:R}$, *b*^1:*K*,1:*R*^, *μ*_*A*_, $\mu_{B}^{1:R}$,***π***, and ***π***_0_). This posterior does not assume an analytical form. As such, we employ the Markov chain Monte Carlo framework to computational sample parameters from this posterior [51, 52, 40, 53]. Briefly, our Gibbs sampler starts with an initial set of values for the parameters and attractively samples new values for each parameter one at a time while holding the others fixed (supplement 5.7).

## 3 Results

Here we demonstrate our method on simulated and experimental data for purposes of model validation. We show that we can accurately learn the number of fluorophores within in an ROI as well as the fluorophore photo-trajectories. We do so robustly even as the number of fluorophores approaches 100. In the supplement, we perform a more detailed robustness analysis on our method using simulated data (supplement 5.10). There, we test our method by varying the number of loads and the number of simulated fluorophores, and the fluorophore state model.

To validate our method on real data, we analyzed brightness traces where fluorophores undergo transitions between photophysical states as they eventually photobleach. This data uses Gattaquant DNA origami constructs with known number of fluorophore binding sites (such that ground truth be known on the total expected number of fluorophores) labelled with ATTO-647N fluorophores with known binding efficiency [26].

Traces with ATTO647N fluorophores examined by us and by others [54] show that ATTO647N has two bright states (see SI section 5.13 for plots of the data traces). We note that in the following sections, our model is supplemented to accommodate a second bright state for the fluorophores in the data we analyze. The expanded model is discussed in supplement 5.5.

### 3.1 Data acquisition

Data acquisition, provided by Hummert and Yserentant et al. [26], is briefly summarized here. ATTO647N labeled DNA oligomers were bound to DNA origami constructs. The DNA origami were imaged using a custom built Nikon Eclipse microscope with total internal reflection fluorescence (TIRF) illumination and a back illuminated EMCCD iXon Ultra 897 camera [26]. A log of Gaussian filter was used to select ROIs. Traces including artifacts such as diffusing fluorophores where excluded. For each ROI at each time level, we summed the brightness of every pixel within the ROI to get the total ROI’s brightness at each time level (i.e., the brightness time trace). We took time traces using two different types of of DNA origami constructs with 20 and 35 binding sites, respectively. For the 20 binding site origami, movies were taken for 1000 seconds at 50ms camera exposure (20000 frames) with a gain of 50. For the 35 binding site origami, movies were taken for 3000 seconds at 200ms camera exposure with an gain of 10 (15000 frames).

In order to analyze traces with more than 20 or 35 fluorophores, we also summed the brightness of every pixel involving multiple ROIs to get the total brightness arising from these combined ROIs at each time level. Because our camera model is a gamma distribution which is closed under addition, this procedure generates controlled traces with a ground truth containing known multiples of 20 or 35 fluorophores.

### 3.2 Results on simulated data

We evaluated our method with data simulated using the forward model put forward in Eqs. (1)–(5) with parameters chosen to mimic real data. We simulated 50 ROIs containing 14 fluorophores on average. The traces are 1000 *s* long with brightness $w_{n}^{r}$ collected every 50 *ms*, so 20000 total frames. The exact number of fluorophores in each ROI is sampled from a binomial distribution to mimic 20 binding sites with 70% labeling efficiency. The gain used for the simulation was 50 [55]. The dimensionless background brightness parameters are $\mu_{B}^{r}=1000$. The fluorophores were simulated with two bright states with brightness given by *μ*_*A*1_ = 450 and *μ*_*A*2_ = 350 (plus one dark state and a photobleached state with brightness given by *μ*_*D*_ = *μ*_*B*_ = 0). These values were chosen to mimic the experimental data that we analyze in section 3.4. For example, the height and duration of a simulated photobleaching event qualitatively match those seen in the real data (see SI section 5.13).

**Figure 3:**
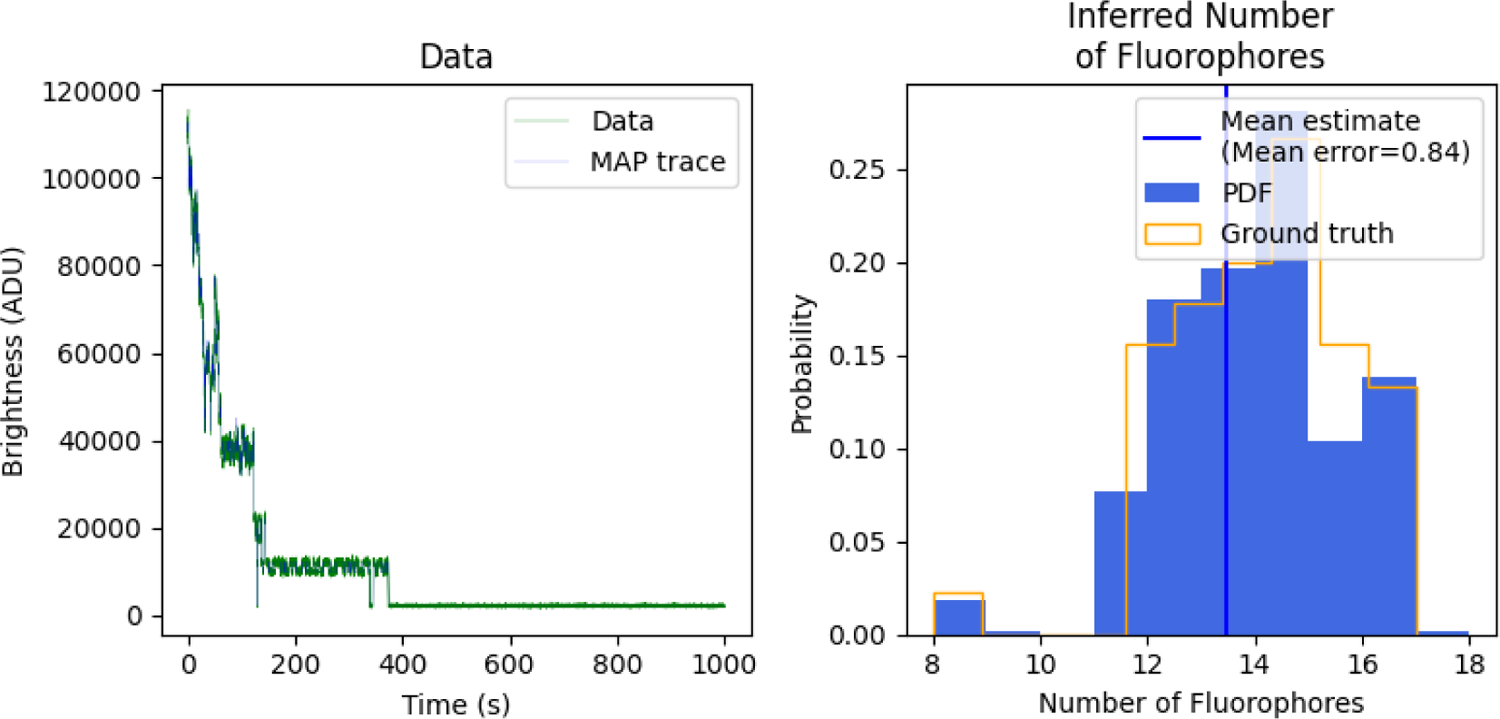
Inference on simulated data. We generated data using the forward model in section 2.1. We learn the number of fluorophores in each ROI and compare to ground truth (right panel) and associated photo-trajectories in each ROI (shown on left panel for one of many ROIs). In addition, we must also simultaneously and self-consistently learn all other associated parameters shown in figure 2.

Figure 3 shows the results for our analysis. The left panel shows the measured brightness versus time trace, superimposed with a sampled mean brightness over time, and the ground truth mean brightness over time. By mean brightness over time we mean the mean expected measurement at each time, $\mu_{n}^{r}$, given the number of fluorophores in each state at that time level, the brightness of each state, and the camera gain. The mean brightness over time directly informs us on the photo-states of the fluorophores; see Eq. 3. Importantly, we capture all brightness drops due to blinking (i.e., photophysical dynamics) that cannot otherwise be obtained using existing PBSA methods that have built into them assumptions 1 and 2 discussed in the introduction.

On the right panel we show posterior over the number of fluorophores per ROI. That is, we find $B_{r}=\sum_{k=1}^{K}b_{k}^{r}$ for each ROI and each sample of our posterior. We then histogram the *B*_*r*_’s for *r* = 1, …, *R*. In the limit that the number of ROIs is large, this should converge to the ground truth distribution of fluorophore numbers marginalized over the uncertainty associated with the number of fluorophores in a single ROI. We calculate the mean error of our method as the average difference between our estimate and the ground truth. Our sampled mean expected brightness trace matches well with the ground truth (within 1 fluorophore). Error analysis shows that roughly half of the samples were equal to the ground truth. No samples were more than 2 fluorophores off.

### 3.3 Comparison against other methods

**Figure 4:**
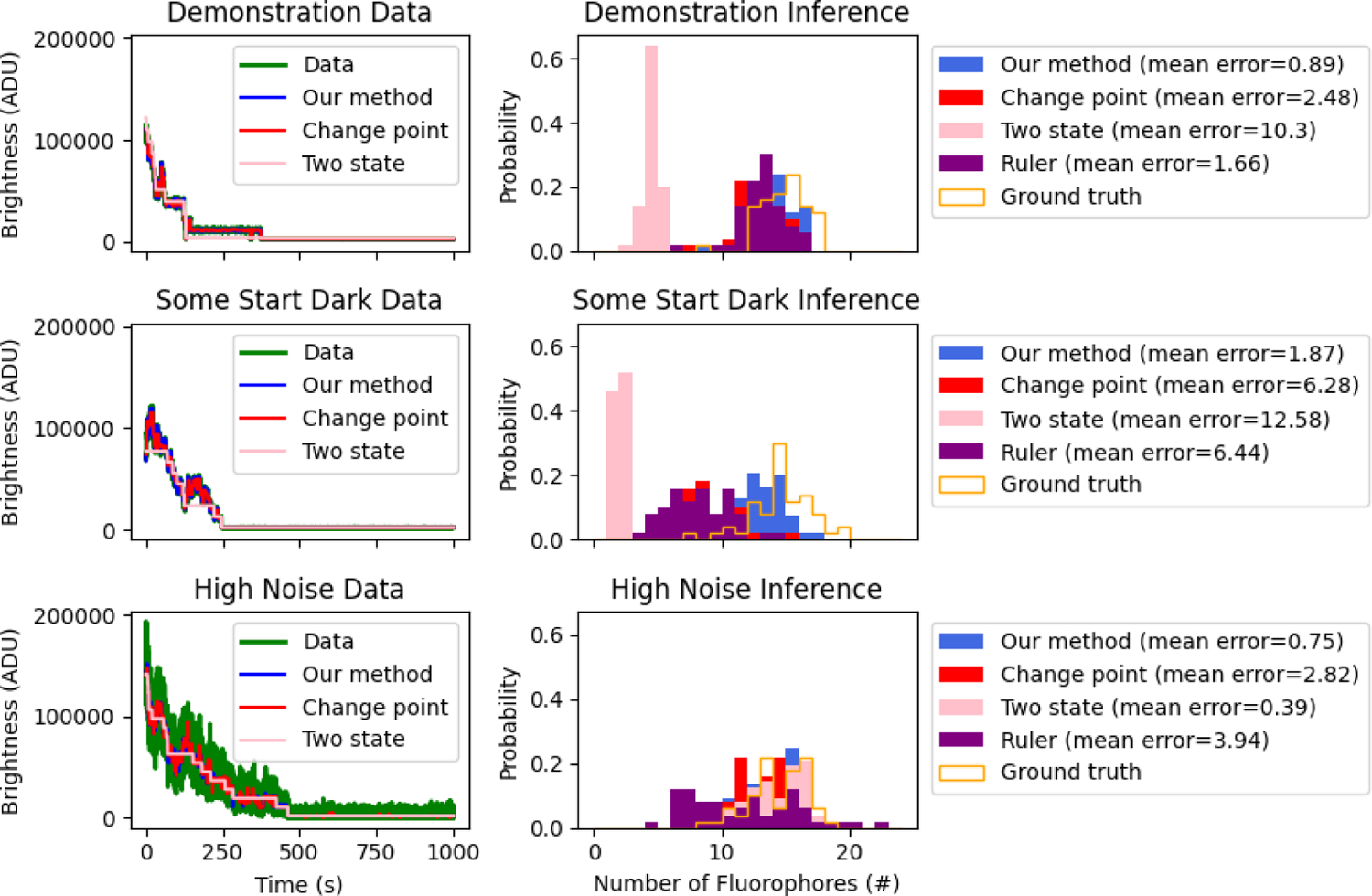
Comparison against other methods. Here we compare our method against a change point method, the ruler method, and the two state model. We compare all methods on three different data sets. On the top row we compare on simulated data using the base set of parameters. On the second row we compare on simulated data in which some fluorophores start dark. On the bottom we compare on simulated data with high noise. The left panel of each row shows the inferred phototrajectory for an ROI using our method, the change point method, and the two state model (the ruler method does not generate trajectories). The right panel shows the inferred distribution for the number of fluorophores for each of the different methods. In the legend we show the mean error of each method calculated as the average difference between the inferred number of fluorophores and the ground truth

Here we compare the results of our method to those obtained using the change point method of Tsekouras et al. [15], the two state model of Garry et al. [25], as well as a ruler method [12, 13]. We note that the Garry et al. method is equivalent to a two state implementation of our own method though they focus on state populations whereas we look at the state of each individual fluorophore. As such, we use our own method, but modified to include only one bright state, one photobleaching state, and no dark state, when comparing our method to the two state model. Our implementation of the ruler method is explained in SI section 5.9. We compare the methods on three different data sets: 1) data simulated using the same parameters as in the demonstration (section 3.2); 2) data simulated in which some fluorophores initiate in the dark state; and 3) data simulated with higher noise. Figure 4 shows the results of our comparison.

As seen in the top row of figure 4, all three methods, besides the two state model, do reasonably well (within 20% error) in inferring the number of fluorophores using the base set of parameters. The two state model underestimates the number of fluorophores due to the fact that it cannot account for blink events (see SI section 5.10.3). Note that the mean error (the average difference between the estimated number of fluorophores in an ROI and the ground truth number of fluorophores used in the simulation) was smallest for our method. That all four methods do well is expected because the data is clean and the steps are easy to see by eye and therefore all three methods should do well at identifying brightness levels and inferring the number of fluorophores.

Next we look at simulated data in which some (40%) of the fluorophores start in the dark state. The second row of figure 4 shows the results. Here, the two state model, the ruler method, and the change point method underestimate the number of fluorophores by over 40% because they do not allow for fluorophores to initiate in a dark. Our method, which allows fluorophores to initiate in such a state, learns the number of fluorophores with less than two fluorophores mean error.

The last row of figure 4 shows results on data simulated with higher noise. The higher noise level was achieved by decreasing the brightnesses, *μ*_*A*_ and *μ*_*B*_. This physically represents lowering the intensity of the laser used to excite the fluorophores down to a level where shot noise dominates. We simultaneously raised the gain to keep the average brightness at the same level. Under these conditions, the two state model no longer underestimates the number of fluorophores as brightness drops arising from blinking events are within the variance of the noise. As such, the two state model becomes reasonable. Our model, which has four states including two bright states and a dark state, has negligibly greater mean error than the two state model in this experiment, due to slight overfitting from having two bright states with brightnesses very close to each other relative to the measurement noise. While the two state model was able to infer the number of fluorophores in the noisy data essentially as accurately as our method, it was unable to do so in the low noise limit (figure 4 top row). As such, our physically-inspired method with photophysical dynamics reveals itself to be most robust across a range of scenarios.

### 3.4 Results on experimental data

**Figure 5:**
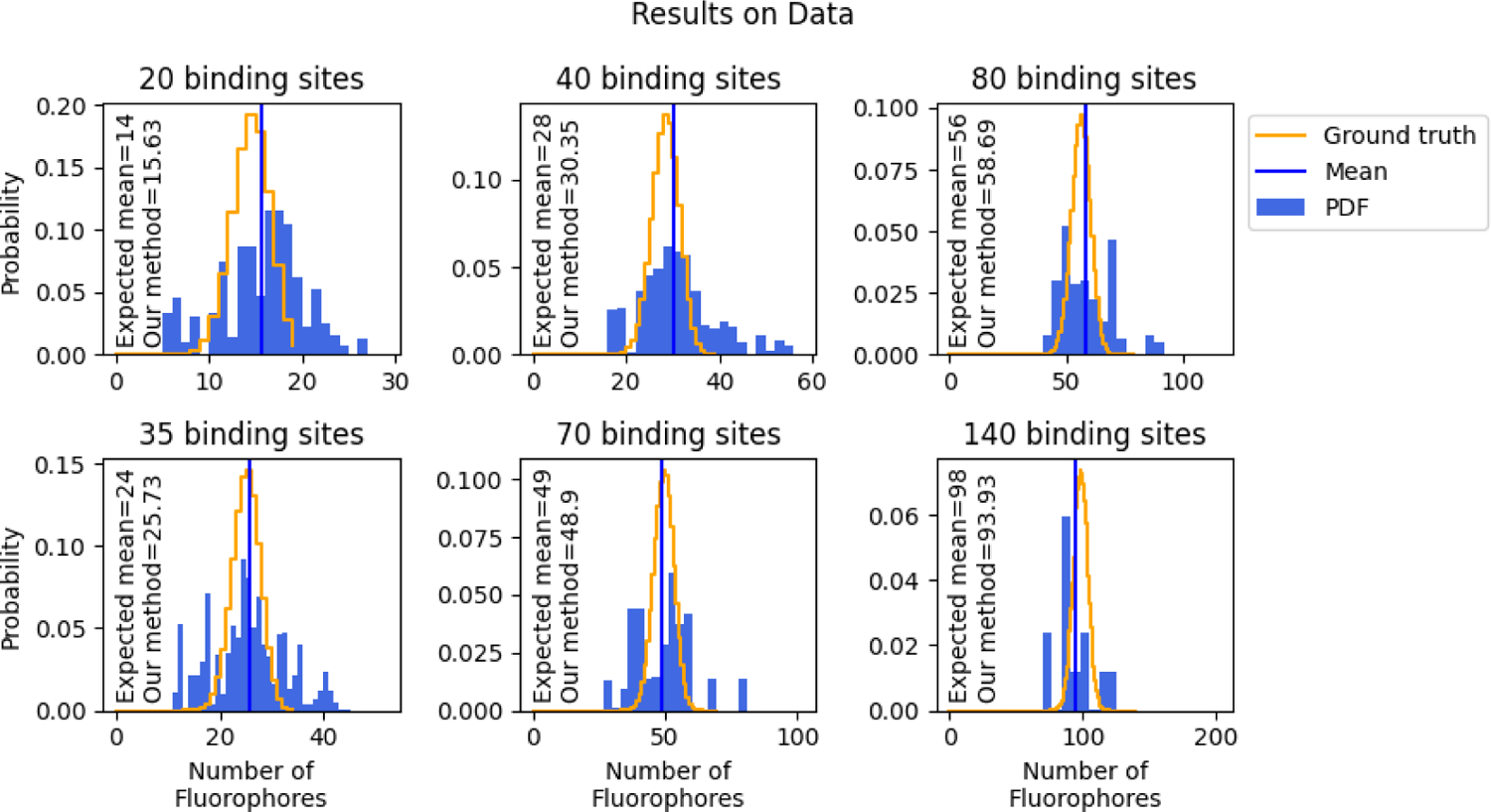
Inference on real data. Here we illustrate our method in enumerating fluorophores from real data. The top row analyzes data from experiments using DNA origami with 20 binding sites (we then combine data from different ROIs to generate data sets with a higher number of fluorophores). Similarly, the bottom row analyzes data from experiments using DNA origami with 35 binding sites (also combined to form ROIs with a larger number of fluorophores). Also plotted are the expected distribution of fluorophores (a binomial distribution), and a vertical line showing the mean expected number of fluorophores learned from our method.

Results from experimental data are shown in the top left and bottom left of figure 5. Here we plot the inferred distribution for the number of fluorophores in an ROI against the ground truth distribution for the number of fluorophores. The ground truth distribution of fluorophores here is binomially distributed [48] assuming a 70% percent labeling efficiency. The 70% labeling efficiency was provided to us by the manufacturer.

We note that the width of the ground truth distribution for the number of fluorophores in the ROI arises due to labeling efficiency of the fluorophores, whereas the width in the distribution of the learned number of fluorophores arises from labeling efficiency as well as uncertainty in the inference. As such, we expect the distribution over the learned number of fluorophores to naturally be wider than the ground truth distribution for the number of fluorophores. For example, in the extreme case where we had 100% labeling efficiency, the ground truth distribution would have zero width, yet our method would still have a width due to uncertainty in the estimate. On the other hand, the mean estimated number of fluorophores in each ROI should be close to the ground truth and thus remains a reliable way by which to evaluate the accuracy of our method.

For our 20 binding site analysis, the predicted mean of the distribution for the number of fluorophores is only about 1.3 fluorophores higher than expected as can be seen in the top left panel of figure 5. This is likely due to overfitting sources of noise not accounted for in our model such as unbound fluorophores freely diffusing above the origami structure.

Given the agreement between ground truth and our method for 20 and 35 binding sites, we wanted to test how high we could count. In order to create controlled data sets with known ground truth, we combined the data from ROIs as discussed in section 3.1. For example, by summing together two ROIs with 20 or 35 binding sites, we could count fluorophores in ROIs with as many as 40 or 70 total binding sites (figure 5 middle column). By adding together four ROIs with 20 or 35 binding sites, we could generate new ROIs with as many as 80 or 140 fluorophores (figure 5 right column). For all four cases, the mean number of fluorophores per ROI learned from our an analysis closely matches (within 3 fluorophores) the ground truth of the expected mean.

## 4 Discussion

Learning the number of molecules located within a molecular cluster, while simultaneously and self-consistently determining the dynamics of the cluster’s constituent members, is a key step toward unraveling life’s processes occurring well below light’s diffraction limit [27, 15, 2, 28, 29, 30, 31, 32, 33, 34, 35, 36, 37, 38, 39, 12, 13, 15, 16, 17, 18, 19, 20, 21, 22, 23, 24, 56]. In order to do so, we introduced a Bayesian nonparametric framework that accurately models the photophysics, shot noise, and detector noise that gives rise to the data, along with sampling methods capable of exploring this high dimensional probability space. Our method was illustrated for as many as 100 fluorophores. We note that the ability to count such a high number of fluorophores is necessary for cellular applications as, for example, nuclear pore complexes are known to be made up from 32 monomers [6, 26], Rac1 can aggregate into clusters of 50–100 [7], and Pol-II can aggregate into clusters of a few hundred [4].

By operating within the Bayesian paradigm, we can propagate uncertainty arising from sources of error, such as photon shot noise and detector signal amplification, into the full distributions over fluorophore numbers and the transition probabilities we determined.

Now, if the counting of fluorophores in a cluster were the only goal and it could be assumed that all fluorophores were initially active, then we could ignore dynamics altogether and avoid learning transition probabilities (as well as trajectories). In this case, a collapsed state formulation (one that keeps track of the total population of decreasing numbers of fluorophores) can be used [15, 25, 26]. However, even then, existing methods for enumeration do not sample full Bayesian posteriors and counting would not be possible for cases where the majority of fluorophores are initially inactive such as in the case of photoactivation localization microscopy (PALM) [57, 58, 59, 11]. Indeed, moving forward, PALM and other superresolution experiments [4, 5] could provide exciting in vivo test beds for our method.

Furthermore, while we have chosen to focus on brightness traces recorded using an EMCCD camera, we could in principle modify our method to allow other detector models. This could be achieved trivially be modifying equation 31 to incorporate the noise model of the desired detector. Moving forward this would allow photobleaching enumeration on a variety of detectors including photomultiplier tubes [60] or sCMOS cameras [61].

The generality afforded by our method in learning dynamics, and thus learning the state of every constituent member of a cluster explicitly, does come at an added computational cost. The majority of the computational cost comes from the forward-backward filtering algorithm used to sample the states. The forward filter backwards sample algorithm (FFBS) runs with time complexity $O\left(S^{2}N\right)$ where *S* is the size of the the state space and *N* is the number of time levels. As we must run the FFBS over each load in each ROI, the total computation time to sample all the states scales like $O\left(S^{2}NRK\right)$ where *R* is the number of ROIs and *K* is the number of loads per ROI. Additionally, in order to facilitate proper mixing of the variables, we sample the states, two loads at a time in a joint state space of size *S*^*J*^ where *J* is the number of loads we sample jointly (see SI section 5.7) which increases the size of the state space, but also decreases the number of times we have to run the FFBS per ROI (for example, sampling two loads at a time means we use FFBS half as many times). As such, overall, the time complexity of our algorithm scales as $O\left(S^{J}NRK/J\right)$. As the majority of the computational bottleneck is ascribed to sampling the states, we therefore sample the states of each ROI in parallel. Computational time can be improved by a factor of *R* if at each iteration of the Gibbs sampler, we sample the states for each ROI (which are independent from each other) in parallel. As the remaining parameters are sampled relatively quickly as compared to state sampling, we sample those globally at each iteration of the Gibbs sampler.

Finally, while we have focused on photophysical dynamics, it is possible to imagine learning the assembly and disassembly kinetics of a cluster. For example, using a two state model where the fluorophores transition between being cluster bound and unbound, our framework could be used to learn the state transition rates as well as the total number of fluorophores bound to the cluster at any given time. Learning such kinetics would be especially relevant to monitoring the formation of large transient protein assemblies relevant to cellular transcription [8, 9, 10]. What remains to be seen is how data could be analyzed if assembly and disassembly of molecules in a cluster are occurring while photophysics of labels on these molecules is simultaneously taking place. In this case, either stable fluorophores that remain in a bright state would need to be used or a difference in timescales between the assembly and disassembly kinetics and photophysical kinetics would need to be sufficiently large to be independently determined by a future analysis method.

## Supplementary Material

1

## Data Availability

The data analyzed in this project was provided by Dirk-Peter Herten, Klaus Yserentant, and Johan Hummert [26]. Brightness traces from ROIs used in this manuscript can be found at http://statphysbio.physics.asu.edu/.
