# Supplementary material for: Diffraction-Limited Molecular Cluster Quantification with Bayesian Nonparametrics
Source: Nat Comput Sci. Author manuscript; Available in PMC 2022 Jul 21. (PMC9302895; doi:10.1038/s43588-022-00197-1)

5 Supplemental Information

5.1 Choice of priors

Priors on initial state probabilities and priors on the rows of the transition probability matrix follow Dirichlet distributions [48, 62, 63, 64]

$$\boldsymbol{\pi}_0 \sim \textbf{Dirichlet}\left(\alpha_0\right) \tag{11}$$

$$\boldsymbol{\pi}_{\sigma_i} \sim \textbf{Dirichlet}\left(\alpha_{\sigma_i}\right) \qquad (i = D, A, B) \tag{12}$$

where α_0 and α_{σ_i} are hyperparameters (discussed further in supplement 5.7). The Dirichlet distributions are chosen for computational convenience alone as these are conjugate to the categorical distribution, Eq. (2); see supplement 5.3. The choice of prior becomes largely immaterial as the data set size increases.

We select gamma distributions as our priors on the mean fluorophore brightness and mean background brightness

$$\mu_A \sim \textbf{Gamma}\left(\phi_{\mu_A}, \psi_{\mu_A}\right) \tag{13}$$

$$\mu_B^r \sim \textbf{Gamma}\left(\phi_{\mu_B}, \psi_{\mu_B}\right). \tag{14}$$

bioRxiv preprint doi: https://doi.org/10.1101/2020.09.28.317057; this version posted September 23, 2021. The copyright holder for this preprint (which was not certified by peer review) is the author/funder, who has granted bioRxiv a license to display the preprint in perpetuity. It is made available under aCC-BY 4.0 International license.

The gamma distribution choice reflects our knowledge that these variables are positive and the hyperparameters are calibrated in such a way as to broaden the range of values these brightnesses may adopt. In particular, by selecting small hyperparameters ($\phi_{\mu_A}, \phi_{\mu_B} = 2$), the prior gives non-negligible probability to a wide range of values for μ_A and μ_B^r; the choice for these scale parameters of the gamma distribution, ψ_{μ_A} and ψ_{μ_B}, is further discussed in supplement 5.7. Once more, the choice of hyperparameters become increasingly unimportant as more data are collected [40, 65].

5.2 Remark on transition probabilities

As shown in figure 1, fluorophores can transition from "dark to bright", "bright to dark", and "bright to photobleached" states as well as self transition from "dark to dark", "bright to bright", and "photobleached to photobleached" states. This is captured by the layout of $\boldsymbol{\pi}$, where rows correspond to the "old state" and columns correspond to the "new state".

$$\boldsymbol{\pi} = \begin{bmatrix} \pi_{\sigma_D \to \sigma_D} & \pi_{\sigma_D \to \sigma_A} & 0 \\ \pi_{\sigma_A \to \sigma_D} & \pi_{\sigma_A \to \sigma_A} & \pi_{\sigma_A \to \sigma_B} \\ 0 & 0 & 1 \end{bmatrix} \tag{15}$$

Here, $\pi_{\sigma_B \to \sigma_B} = 1$ because there are no transitions out of the photobleached state. Similarly, $\boldsymbol{\pi}_0$, the initial state probability, takes the form

$$\boldsymbol{\pi}_0 = \begin{bmatrix} \pi_{0 \to \sigma_D} & \pi_{0 \to \sigma_A} & 0 \end{bmatrix}. \tag{16}$$

5.3 Conjugate prior on transition probabilities

The choice of Dirichlet prior on the rows of $\boldsymbol{\pi}$ combined with the likelihood give back a Dirichlet distribution [48].

Proof:

Using Bayes's theorem, and dropping all terms that do not depend on $\boldsymbol{\pi}_0$ and $\boldsymbol{\pi}$, the likelihood of our model is

$$\begin{aligned}
&\mathcal{P}\left(s_{1:N}^{1:K,1:R}, b^{1:K,1:R}, \mu_A^{1:R}, \mu_B^{1:R}, G, \boldsymbol{\pi}, \boldsymbol{\pi}_0 | w_{1:N}^{1:R}, \Omega\right) \\
&\propto \mathcal{P}\left(w_{1:N}^{1:R} | s_{1:N}^{1:K,1:R}, b^{1:K,1:R}, \mu_A^{1:R}, \mu_B^{1:R}, G, \boldsymbol{\pi}, \boldsymbol{\pi}_0, \Omega\right) \times \mathcal{P}(\boldsymbol{\pi}, \boldsymbol{\pi}_0) \\
&\propto \left(\prod_{r=1}^{R}\prod_{k=1}^{K} \mathcal{P}\left(s_1^{k,r}|\boldsymbol{\pi}_0\right) \prod_{n=2}^{N} \mathcal{P}\left(s_n^{k,r}|s_{n-1}^{k,r}, \boldsymbol{\pi}\right)\right) \left(\textbf{Dirichlet}\left(\boldsymbol{\pi}_0|\alpha_{\boldsymbol{\pi}_0}\right) \prod_i \textbf{Dirichlet}\left(\pi_{\sigma_i}|\alpha_{\pi_{\sigma_i}}\right)\right) \\
&\propto \left(\prod_{r=1}^{R}\prod_{k=1}^{K} \pi_{0,\sigma_D}^{[s_1^{k,r}=\sigma_D]} \pi_{0,\sigma_A}^{[s_1^{k,r}=\sigma_A]} \prod_{n=2}^{N}\prod_{i,j} \pi_{i,j}^{[s_{n-1}^{k,r}=\sigma_i][s_n^{k,r}=\sigma_j]}\right) \left(\pi_{0,\sigma_D}^{\alpha_{0,\sigma_D}-1} \pi_{0,\sigma_A}^{\alpha_{0,\sigma_A}-1} \prod_{i,j} \pi_{\sigma_i,\sigma_j}^{\alpha_{\sigma_i,\sigma_j}-1}\right) \\
&\propto \left(\pi_{0,\sigma_D}^{C_{0,D}} \pi_{0,\sigma_A}^{C_{0,A}} \prod_{i,j} \pi_{i,j}^{C_{i,j}}\right) \left(\pi_{0,\sigma_D}^{\alpha_{0,\sigma_D}-1} \pi_{0,\sigma_A}^{\alpha_{0,\sigma_A}-1} \prod_{i,j} \pi_{\sigma_i,\sigma_j}^{\alpha_{\sigma_i,\sigma_j}-1}\right) \\
&\propto \pi_{0,\sigma_D}^{C_{0,D}+\alpha_{0,\sigma_D}-1} \pi_{0,\sigma_A}^{C_{0,A}+\alpha_{0,\sigma_A}-1} \prod_{i,j} \pi_{\sigma_i,\sigma_j}^{C_{i,j}+\alpha_{\sigma_i,\sigma_j}-1} \\
&\propto \textbf{Dirichlet}\left(\boldsymbol{\pi}_0|C_0 + \alpha_{\boldsymbol{\pi}_0}\right) \prod_i \textbf{Dirichlet}\left(\pi_{\sigma_i}|C_i + \alpha_{\pi_{\sigma_i}}\right)
\end{aligned}$$

where $C_{0,D}$ and $C_{0,A}$ are the number of fluorophores that started in states σ_D, σ_A, and $C_{i,j}$ is the number of fluorophores that transitioned from σ_i to σ_j, C_0 and C_i are the arrays containing the counts $C_{i,j}$, and the products indexed by i and j go over σ_D, σ_A, and σ_B. So the Dirichlet prior on the initial state probability and transition probabilities allow us to get back independent Dirichlet distributions in the posterior.

bioRxiv preprint doi: https://doi.org/10.1101/2020.09.28.317057; this version posted September 23, 2021. The copyright holder for this preprint (which was not certified by peer review) is the author/funder, who has granted bioRxiv a license to display the preprint in perpetuity. It is made available under aCC-BY 4.0 International license.

Notice that all elements of $\boldsymbol{\pi}_0$ and $\boldsymbol{\pi}$ that are equal to zero also have zero counts in the trace. By declaring $0^0 = 1$, these components do not affect the probability.

5.4 Hyperparameter on loads

Each load is sampled from a Bernoulli distribution with a parameter $q^{k,r}$. This parameter $q^{k,r}$ is sampled from a beta distribution with hyperparameters γ and K, where γ is a hyperhyperparameter on all $q^{k,r}$'s and K is the total number of load on and load off fluorophores in ROI r.

$$b^{k,r} \sim \mathbf{Bernoulli}\left(q^{k,r}\right) \tag{17}$$

$$q^{k,r} \sim \mathbf{Beta}\left(\frac{\gamma}{K}, \frac{K-1}{K}\right) \tag{18}$$

This is the Beta-Bernoulli process (BBP) [42, 43]. The distribution on $q^{k,r}$ is constructed in such a way that as K goes to infinity, $q^{k,r}$ goes to zero consistent with our assumption that $K \gg K_r$. Because $q^{k,r}$ is a hyperparameter, it is computationally advantageous for us to marginalize it out. We marginalize over the $q^{k,r}$'s to get a Bernoulli distribution for $b^{k,r}$ in terms of γ and K.
Proof (with indices dropped for clarity):

$$\begin{aligned}
\mathcal{P}(b) &= \int_0^1 dq\ \mathcal{P}(b|q)\,\mathcal{P}(q) \\
&= \int_0^1 dq\ \mathbf{Bernoulli}(b|q)\ \mathbf{Beta}\left(q|\frac{\gamma}{K}, \frac{K-1}{K}\right) \\
&= \int_0^1 dq\left(q^b (1-q)^{1-b}\right)\left(\frac{\Gamma\left(\frac{\gamma}{K}+\frac{K-1}{K}\right)}{\Gamma\left(\frac{\gamma}{K}\right)\Gamma\left(\frac{K-1}{K}\right)} q^{\frac{\gamma}{K}}(1-q)^{\frac{K-1}{K}}\right) \\
&= \frac{\Gamma\left(\frac{\gamma}{K}+\frac{K-1}{K}\right)}{\Gamma\left(\frac{\gamma}{K}\right)\Gamma\left(\frac{K-1}{K}\right)}\int_0^1 dq\ q^{\frac{\gamma}{K}+b-1}(1-q)^{\frac{K-1}{K}-b+1-1} \\
&= \frac{\Gamma\left(\frac{\gamma}{K}+\frac{K-1}{K}\right)}{\Gamma\left(\frac{\gamma}{K}\right)\Gamma\left(\frac{K-1}{K}\right)}\frac{\Gamma\left(\frac{\gamma}{K}+b\right)\Gamma\left(\frac{K-1}{K}-b+1\right)}{\Gamma\left(\frac{\gamma}{K}+\frac{K-1}{K}+1\right)}\int_0^1 dq\ \mathbf{Beta}\left(q|\frac{\gamma}{K}+b, \frac{K-1}{K}-b+1\right) \\
&= \frac{K}{\gamma+K-1}\frac{\Gamma\left(\frac{\gamma}{K}+b\right)}{\Gamma\left(\frac{\gamma}{K}\right)}\frac{\Gamma\left(\frac{K-1}{K}-b+1\right)}{\Gamma\left(\frac{K-1}{K}\right)} \\
&= \begin{cases} \frac{K-1}{\gamma+K-1} & b=0 \\ \frac{\gamma}{\gamma+K-1} & b=1 \end{cases} \\
&= \mathbf{Bernoulli}\left(\frac{\gamma}{\gamma+K-1}\right).
\end{aligned}$$

Following our assumption, $K \gg K_r$, $\gamma/(\gamma+K-1)$ must be a small number (we use $\gamma/(\gamma+K-1) = .1$ with $K = 100$). Beyond this restriction, we can set γ by hand knowing that its effect is minimal for sufficient data. Altogether we have

$$b^{k,r} \sim \mathbf{Bernoulli}\left(\frac{\gamma}{\gamma+K-1}\right). \tag{19}$$

5.5 Multiple bright states

Here instead of having one active state, σ_A, we will have L active states, $\sigma_{A1}, \ldots, \sigma_{AL}$ each with brightness $\mu_{A1}, \ldots \mu_{AL}$. We note that L must be selected *a priori*. The transition matrix is now an $L+2$ by $L+2$

bioRxiv preprint doi: https://doi.org/10.1101/2020.09.28.317057; this version posted September 23, 2021. The copyright holder for this preprint (which was not certified by peer review) is the author/funder, who has granted bioRxiv a license to display the preprint in perpetuity. It is made available under aCC-BY 4.0 International license.

matrix

$$\boldsymbol{\pi} = \begin{bmatrix} \pi_{\sigma_D \to \sigma_D} & \pi_{\sigma_D \to \sigma_{A1}} & \pi_{\sigma_D \to \sigma_{A2}} & \cdots & 0 \\ \pi_{\sigma_{A1} \to \sigma_D} & \pi_{\sigma_{A1} \to \sigma_{A1}} & \pi_{\sigma_{A1} \to \sigma_{A2}} & \cdots & \pi_{\sigma_{A1} \to \sigma_B} \\ \pi_{\sigma_{A2} \to \sigma_D} & \pi_{\sigma_{A2} \to \sigma_{A1}} & \pi_{\sigma_{A2} \to \sigma_{A2}} & \cdots & \pi_{\sigma_{A2} \to \sigma_B} \\ \cdots & \cdots & \cdots & \cdots & \cdots \\ 0 & 0 & 0 & \cdots & 1 \end{bmatrix}. \tag{20}$$

We note that as we add these bright states, we must add L different random variables $(\mu_{A1}, \ldots \mu_{AL})$.

5.6 Model summary and posterior

The model is summarized as follows.

$$\mu_A \sim \textbf{Gamma}\left(\phi_{\mu_A}, \psi_{\mu_A}\right) \tag{21}$$

$$\mu_B^r \sim \textbf{Gamma}\left(\phi_{\mu_B}, \psi_{\mu_B}\right) \qquad r = 1, \ldots, R \tag{22}$$

$$\boldsymbol{\pi}_0 \sim \textbf{Dirichlet}\left(\alpha_0\right) \tag{23}$$

$$\boldsymbol{\pi}_{\sigma_i} \sim \textbf{Dirichlet}\left(\alpha_{\sigma_i}\right) \qquad i = D, B, A \tag{24}$$

$$b^{k,r} \sim \textbf{Bernoulli}\left(\frac{\gamma}{K + \gamma - 1}\right) \qquad k = 1, \ldots, K;\ r = 1, \ldots, R \tag{25}$$

$$s_1^{k,r} | \boldsymbol{\pi}_0 \sim \textbf{Categorical}\left(\boldsymbol{\pi}_0\right) \tag{26}$$

$$s_n^{k,r} | s_{n-1}^{k,r}, \boldsymbol{\pi} \sim \textbf{Categorical}\left(\boldsymbol{\pi}_{s_{n-1}^{k,r}}\right) \qquad k = 1, \ldots, K;\ r = 1, \ldots, R \tag{27}$$

$$w_n^r | s_n^{1:K,r}, b^{1:K,r}, \mu_A, \mu_B^r \sim \textbf{Gamma}\left((\mu_B^r + \sum_{k=1}^{K} b^{k,r} \mu_{s_n^{k,r}})/2, 2G\right) \qquad n = 1, \ldots, N;\ r = 1, \ldots, R \tag{28}$$

$$s_N^{k,r} = \sigma_B. \tag{29}$$

The inverse model scheme (section 2.2) allow us to develop the joint posterior of our model

$$\begin{aligned} &\mathcal{P}\left(s_{1:N}^{1:K,1:R}, b^{1:K,1:R}, \mu_A, \mu_B^{1:R}, \boldsymbol{\pi}, \boldsymbol{\pi}_0 | w_{1:N}^{1:R}\right) \\ &\propto \mathcal{P}\left(w_{1:N}^{1:R} | s_{1:N}^{1:K,1:R}, b^{1:K,1:R}, \mu_A, \mu_B^{1:R}, \boldsymbol{\pi}, \boldsymbol{\pi}_0\right) \\ &\times \mathcal{P}\left(s_{1:N}^{1:K,1:R}, b^{1:K,1:R}, \mu_A, \mu_B^{1:R}, \boldsymbol{\pi}, \boldsymbol{\pi}_0\right) \end{aligned} \tag{30}$$

where the likelihood (middle term of Eq. (30)) is the product of Eq. (31) for all time levels and ROIs, which looks like

$$\begin{aligned} &\mathcal{P}\left(w_{1:N}^{1:R} | s_{1:N}^{1:K,1:R}, b^{1:K,1:R}, \mu_A, \mu_B^{1:R}, \boldsymbol{\pi}, \boldsymbol{\pi}_0\right) \\ &= \prod_{r=1}^{R} \prod_{n=1}^{N} \textbf{Gamma}\left(w_n^r; (\mu_B^{r,n} + \sum_{k=1}^{K} b^{k,r} \mu_{s_n^{k,r}})/2, 2G\right), \end{aligned} \tag{31}$$

and the prior (final term) of can be further decomposed into

$$\begin{aligned} &\mathcal{P}\left(s_{1:N}^{1:K,1:R}, b^{1:K,1:R}, \mu_A, \mu_B^{1:R}, \boldsymbol{\pi}, \boldsymbol{\pi}_0\right) \\ &\propto \mathcal{P}\left(b^{1:K,1:R}\right) \mathcal{P}\left(\mu_A\right) \mathcal{P}\left(\mu_B^{1:R}\right) \mathcal{P}\left(\boldsymbol{\pi}\right) \mathcal{P}\left(\boldsymbol{\pi}_0\right) \\ &\times \mathcal{P}\left(s_{1:N-1}^{1:K,1:R} | \boldsymbol{\pi}, \boldsymbol{\pi}_0\right) \end{aligned} \tag{32}$$

which has two main parts, 1) the middle terms of Eq. (32) are the product of Eqs. (11)-(6) over all indexes 2) the last term of Eq. (32) is the product of $\pi_{0,D}$ to the power of the number of fluorophores that start dark (C_{0D} is the number of fluorophores that start dark) times $\pi_{0,A}$ to the power of the number of

bioRxiv preprint doi: https://doi.org/10.1101/2020.09.28.317057; this version posted September 23, 2021. The copyright holder for this preprint (which was not certified by peer review) is the author/funder, who has granted bioRxiv a license to display the preprint in perpetuity. It is made available under aCC-BY 4.0 International license.

fluorophores that start bright (C_{0A} is the number of fluorophores that start bright) times π_{ij} to the power of the number of transitions that occurred from σ_i to σ_j (C_{ij} is the number of transitions from σ_i to σ_j)

$$\mathcal{P}\left(s_{1:N-1}^{1:K,1:R}|\boldsymbol{\pi},\boldsymbol{\pi}_0\right) = \pi_{O,D}^{C_{0D}}\pi_{0,A}^{C_{0A}}\prod_{i,j}\pi_{ij}^{C_{ij}} \tag{33}$$

$$C_{0D} = \sum_{r=1}^{R}\sum_{k=1}^{K} I[s_1^{k,r} = \sigma_D] \tag{34}$$

$$C_{0A} = \sum_{r=1}^{R}\sum_{k=1}^{K} I[s_1^{k,r} = \sigma_A] \tag{35}$$

$$C_{ij} = \sum_{r=1}^{R}\sum_{k=1}^{K}\sum_{n=2}^{N} I[s_n^{k,r} = \sigma_j]I[s_{n-1}^{k,r} = \sigma_i] \tag{36}$$

where $I[x = y]$ is the indicator function that is equal to 1 if $x = y$ and 0 else. We note that in this scheme (Eqs. (30)-(33)), we are learning the number of fluorophores in many ROI simultaneously. This is important because by analyzing many ROI together, we effectively have more information to train on and therefore obtain more accurate results.

5.7 Sampling

We outline the Gibbs sampling scheme below where (i) indexes the iteration in the Gibbs sampler.

- Step 1: Choose initial $s_{1:N}^{1:K,1:R,(0)}$, $b^{1:K,1:R,(0)}$, $\mu_A^{(0)}$, $\mu_B^{1:R,(0)}$, $\boldsymbol{\pi}^{(0)}$, and $\boldsymbol{\pi}_0^{(0)}$.
- Step 2: For many iterations, i:
  - A) Sample new states and loads from

    $$\mathcal{P}\left(s_{1:N}^{1:K,1:R,(i)}, b^{1:K,1:R,(i)}|\mu_A^{(i-1)}, \mu_B^{1:R,(i-1)}, \boldsymbol{\pi}^{(i-1)}, \boldsymbol{\pi}_0^{(i-1)}, w_{1:N}^{1:R}\right)$$

    which is the product of Eq. (31) and Eq. (33).
  - B) Sample new transition probabilities and initial state probabilities from

    $$\mathcal{P}\left(\boldsymbol{\pi}^{(i)}, \boldsymbol{\pi}_0^{(i)}|s_{1:N}^{1:K,1:R,(i)}, b^{1:K,1:R,(i)}, \mu_A^{(i-1)}, \mu_B^{1:R,(i-1)}, w_{1:N}^{1:R}\right)$$

    which is the product of Eqs. (11), (12), and (33). Here the choice of conjugate priors allow us to sample $\boldsymbol{\pi}_0$ and $\boldsymbol{\pi}$ directly.
  - C) Sample new camera parameters from

    $$\mathcal{P}\left(\mu_A^{(i)}, \mu_B^{1:R,(i)}|s_{1:N}^{1:K,1:R,(i)}, b^{1:K,1:R,(i)}, \boldsymbol{\pi}^{(i)}, \boldsymbol{\pi}_0^{(i)}, w_{1:N}^{1:R}\right)$$

    which is the product of Eq. (31) and the priors (Eqs. (13)-(14)).

For Step 1, the initial values are chosen to be the means of the prior. These means can in principle be anything, although, better guesses lead to faster convergence. We did notice the sampler is sensitive to the initial value for the fluorophore brightness. In other words we could sample, for example, half as many fluorophores with twice the brightness or three times the number of fluorophore with a third the brightness. To avoid this problem we set a sharp prior for the value of μ_A on the brightness of a single fluorophore which is clear at the end of the trace. This makes sure that our model posterior is sharply peaked at reasonable numbers of fluorophores. We calibrate gain, G, using methods described in Hirsch et al [44]. We estimate the background brightness for each ROI by looking at the end of the brightness

bioRxiv preprint doi: https://doi.org/10.1101/2020.09.28.317057; this version posted September 23, 2021. The copyright holder for this preprint (which was not certified by peer review) is the author/funder, who has granted bioRxiv a license to display the preprint in perpetuity. It is made available under aCC-BY 4.0 International license.

time trace, when all fluorophores are presumed to be photobleached. For $\boldsymbol{\pi}_0^{(0)}$ we take the maximally naive approach and choose the starting probability for bright and dark to be 50/50. We can choose $\boldsymbol{\pi}^{(0)}$ using this approach, but we find that choosing self transitions to be larger speeds up the convergence significantly (by about 100-500 iterations) For $s_{1:N}^{(0),1:R,1:K}$ we guess that there are no fluorophores in any ROI, i.e that for all loads, $b^{k,r} = 0$. In our analysis, we chose the hyperparameters to coincide with the initial guess. So $\alpha_0 = [.5, .5, 0]$, $\alpha_{\sigma_D} = [.9, .1, 0]$, $\alpha_{\sigma_A} = [.1, .8, .1]$, $\alpha_{\sigma_B} = [0, 0, 1]$. For the mean background brightness and mean fluorophore brightness, we chose the initial guess to be the mean of the prior by scaling the scale parameter by the shape parameter,

$$\psi_{\mu_A} = \mu_A^{(0)}/\phi_{\mu_A}$$
$$\psi_{\mu_B}^r = \mu_B^{r,(0)}/\phi_{\mu_B}$$

In Step 2 A, for each ROI, we sample loads and states together by using the forward filter backwards sample algorithm [48]. To speed up computation we do this using a collapsed state space where we treat photobleached fluorophores and fluorophores with load off to be the same. That is, under our model each fluorophore is described by two different random variables, its load and its state. However, we instead describe it with one random variable by lumping together fluorophores with off loads and photobleached fluorophores with on loads. Consider a new state space defined by

$$\sigma'_D = \{b^{k,r} = 1 \text{ and } s_n^{k,r} = \sigma_D\} \tag{37}$$
$$\sigma'_A = \{b^{k,r} = 1 \text{ and } s_n^{k,r} = \sigma_A\} \tag{38}$$
$$\sigma'_B = \{(b^{k,r} = 1 \text{ and } s_n^{k,r} = \sigma_B) \text{ or } (b^{k,r} = 0)\} \tag{39}$$

In this "collapsed state space", we ignore the loads entirely, but allow for fluorophores to start from the photobleached state with probability $(K-1)/(\gamma+K-1)$ (the probability that a fluorophore has load off). This in turn causes $\boldsymbol{\pi}_0$ to scale down by $\gamma/(\gamma+K-1)$ as well,

$$\boldsymbol{\pi}_0 = \left[\frac{\gamma}{\gamma+K-1}\pi_{0D}, \frac{\gamma}{\gamma+K-1}\pi_{0A}, \frac{K-1}{\gamma+K-1}\pi_{0B}\right] \tag{40}$$

but notice that $\boldsymbol{\pi}$ is not affected (as off load fluorophores cannot become on and vice versa) nor are any of the other random variables. Thus we can simplify our calculations by simply allowing load off fluorophores to be considered photobleached so long as we keep track of $\boldsymbol{\pi}_0$. Additionally, rather than sample each $s_n^{k,r}$ individually, we sample $s_n^{k,r}$, $s_n^{k+1,r}$,... $s_n^{k+J,r}$ together (for $J = 4$ in our case). We additionally shuffle the fluorophore indices k so that at each sampler iteration, different fluorophores are sampled together. Sampling fluorophore states together in this way helps the sampler mixing. The joint microstate, $S_n^{k,r}$, specifies the states of the fluorophores when we consider them together. For example, one realization of a joint microstate might look like

$$S_n^{k,r} = \Sigma_{17}$$
$$S_n^{k,r} = \{s_n^{k,r} = \sigma'_D,\ s_n^{k+1,r} = \sigma'_A,\ s_n^{k+2,r} = \sigma'_B,\ s_n^{k+3,r} = \sigma'_A,\ s_n^{k+4,r} = \sigma'_D,\ s_n^{k+5,r} = \sigma'_B\},$$

or using a notation $\Sigma_1, \Sigma_2, \ldots$ to represent the possible joint microstates that $S_n^{k,r}$ can take,

$$S_n^{k,r} = \Sigma_{17}$$
$$\Sigma_{17} = \{\sigma'_D, \sigma'_A, \sigma'_B, \sigma'_A, \sigma'_D, \sigma'_B\},$$

where the index, 17, on Σ_{17} is used simply to illustrate an example. The number of values $S_n^{k,r}$ can take $(\Sigma_1, \Sigma_2, \ldots)$ is the Kronecker product of the individual state spaces $(\{\sigma'_D, \sigma'_A, \sigma'_B\})$. For Step 2 C, we sample μ_A and $\mu_B^{1:R}$ each separately using a Hamiltonian Monte Carlo step [66].

As the sampler runs, higher probability regions are sampled more often and lower probability regions are sampled less often [51, 52, 40, 53]. After many iterations we can histogram the results to approximate the shape of our posterior. By looking at the histogram and not just the MAP or mean, we are able to get credible intervals for our estimates.

bioRxiv preprint doi: https://doi.org/10.1101/2020.09.28.317057; this version posted September 23, 2021. The copyright holder for this preprint (which was not certified by peer review) is the author/funder, who has granted bioRxiv a license to display the preprint in perpetuity. It is made available under aCC-BY 4.0 International license.

We note that the R ROIs in the base set are analyzed simultaneously. To be clear, we mean that each ROI has its own fluorophore states, loads, and mean background brightness, but the transition probabilities, initial state probability, and mean fluorophore brightness are global. This is advantageous because it allows the inference of each ROI to benefit from the others by providing more data on which to train the transition probabilities.

5.8 Hamiltonian Monte Carlo

We sampled our state brightness and background brightness using Hamiltonian Monte Carlo [66]. Briefly, Hamiltonian Monte Carlo proposes samples using a numerical integrator with strong parallels to Hamiltonian dynamics. Here, our random variables are sampled along with normally distributed auxiliary variables, referred to as momenta, p_A and p_B

$$\mathcal{P}\left(\mu_A, \mu_B|\boldsymbol{w}\right) = \int dp_A \int dp_B \mathcal{P}\left(\mu_A, \mu_B|\boldsymbol{w}\right)\mathcal{P}\left(\boldsymbol{p}\right) \tag{41}$$

$$= \int dp_A \int dp_B \mathcal{P}\left(\mu_A, \mu_B|\boldsymbol{w}\right) \textbf{Normal}\left(p_A; 0, m_A\right) \textbf{Normal}\left(p_B; 0, m_B\right) \tag{42}$$

where m_A and m_B, called the masses, are the variances of the priors on the momenta (for simplicity in the derivation we only look at a single bright state and single background brightness). The negative log of the conditional posterior, including the momenta, is called the Hamiltonian.

$$\mathcal{H} = -\log(\mathcal{P}\left(\mu_A, \mu_B|\boldsymbol{w}\right)\mathcal{P}\left(\boldsymbol{p}\right)) \tag{43}$$

$$= -\log\Bigg(\textbf{Gamma}\left(\mu_A; \alpha_{\mu_A}, \theta_{\mu_A}\right) \textbf{Gamma}\left(\mu_B; \alpha_{\mu_B}, \theta_{\mu_B}\right) \tag{44}$$

$$\times \left(\prod_{n=1}^{N} \textbf{Gamma}\left(w_n; \mu_n/2, 2G\right)\right) \tag{45}$$

$$\times \textbf{Normal}\left(p_A; 0, m_A\right) \textbf{Normal}\left(p_B; 0, m_B\right)\Bigg) \tag{46}$$

$$= \frac{p_A^2}{2m_A} + \frac{p_B^2}{2m_B} + \frac{\mu_A}{\theta_{\mu_A}} - (\alpha_A - 1)\log(\mu_A) + \frac{\mu_B}{\theta_{\mu_B}} - (\alpha_B - 1)\log(\mu_B) \tag{47}$$

$$+ \sum_{n=1}^{N} \left(\log(\Gamma(\mu_n/2)) - (\mu_n/2 - 1)\log(w_n/2G)\right) + C \tag{48}$$

where $\mu_n = \mu_B + X_n\mu_A$ with X_n is the number of fluorophores in the bright state at time level n, and where C includes all terms not dependent on p_A, p_B, μ_A, or mu_B. Note that the momenta in the Hamiltonian appear exactly like kinetic energy terms (motivating the name Hamiltonian Monte Carlo). We may use Hamilton's equations of motion to modify p_A, p_B, μ_A, and μ_B in such a way that the total

bioRxiv preprint doi: https://doi.org/10.1101/2020.09.28.317057; this version posted September 23, 2021. The copyright holder for this preprint (which was not certified by peer review) is the author/funder, who has granted bioRxiv a license to display the preprint in perpetuity. It is made available under aCC-BY 4.0 International license.

"energy" of the system is conserved

$$\frac{\partial \mu_A}{\partial t} = \frac{\partial \mathcal{H}}{\partial p_A} \tag{49}$$

$$= \frac{p_A}{m_A} \tag{50}$$

$$\frac{\partial \mu_B}{\partial t} = \frac{\partial \mathcal{H}}{\partial p_B} \tag{51}$$

$$= \frac{p_B}{\mu_B} \tag{52}$$

$$\frac{\partial p_A}{\partial t} = -\frac{\partial \mathcal{H}}{\partial \mu_A} \tag{53}$$

$$= -\frac{1}{\theta_{\mu_A}} + \frac{\alpha_{\mu_A} - 1}{\mu_A} - \frac{1}{2}\sum_{n=1}^{N} X_n \left(\psi(\mu_n/2) - \log(w_n/2G)\right) \tag{54}$$

$$\frac{\partial \mu_B}{\partial t} = -\frac{\partial \mathcal{H}}{\partial p_B} \tag{55}$$

$$= -\frac{1}{\theta_{\mu_A}} + \frac{\alpha_{\mu_A} - 1}{\mu_A} - \frac{1}{2}\sum_{n=1}^{N} \left(\psi(\mu_n/2) - \log(w_n/2G)\right). \tag{56}$$

Conservation of energy in this case corresponds exactly to conservation of probability in the posterior. In principle, this means that starting with sampled values for p_A and p_B, we can use a Hamiltonian mechanics integrator to find proposal values of μ_A and μ_B with equal probability as those from the previous Gibbs sampler iteration.

5.9 Ruler method

The ruler method estimates the number of fluorophores by dividing the initial brightness by the estimated fluorophore brightness. For our implementation of the ruler method we average the last 10% of the brightness trace to get an estimate for the background brightness and we average the first 10 data points to get an estimate for the initial brightness. To estimate the brightness of a single fluorophore we average the last ten frames of the final photobleaching step less the background. The final photobleaching step is found by looking for the last time level where the brightness is above a set threshold. For our purposes, this threshold is always chosen to be the same brightness guess that we use to inform our prior on brightness, except for the high noise experiment (figure 4 bottom row) in which the high noise causes the background brightness to be frequently measured higher than the fluorophore brightness step. For the high noise case, we set the threshold to be 3 times the fluorophore brightness guess, which is high enough that the background is excluded, but low enough that it is sampled in the final photobleaching step (see figure 5.13). We note that setting the brightness of a fluorophore by hand can improve the ruler method's accuracy. However, as our method, the change point method, and the two state method simultaneously learn fluorophore brightness with the number of fluorophores, we require that the ruler method also learn the fluorophore brightness in establish a fair comparison.

5.10 Robustness analysis on simulated data

In this section we evaluate the robustness of our method. In the main (section 3.2), we demonstrated the method using a "base set of parameters". For the base set we have 50 ROIs containing 14 fluorophores on average. The traces are 1000 seconds long with brightness w_n^r collected every 50 ms, so 20000 total frames. The exact number of fluorophores in each ROI is sampled from a binomial distribution to mimic 20 binding sites with 70% labeling efficiency. The gain used for the simulation was 50. The dimensionless background brightness parameters are $\mu_B^r = 1000$. The fluorophores were simulated with two bright states with brightness given by $\mu_{A1} = 450$ and $\mu_{A2} = 350$ (plus one dark state and a photobleached state with brightness given by $\mu_D = \mu_B = 0$). For the number of loads, K, we chose 39. These parameters were chosen to mimic the parameters learned from real data, for example, the brightness states (μ_{A1} and μ_{A2})

bioRxiv preprint doi: https://doi.org/10.1101/2020.09.28.317057; this version posted September 23, 2021. The copyright holder for this preprint (which was not certified by peer review) is the author/funder, who has granted bioRxiv a license to display the preprint in perpetuity. It is made available under aCC-BY 4.0 International license.

were chosen so that the height of a photobleaching step in the simulated data is the same height as a photobleaching step in the real data. We will refer to these parameters as the "base set."

5.10.1 Varying the number of loads

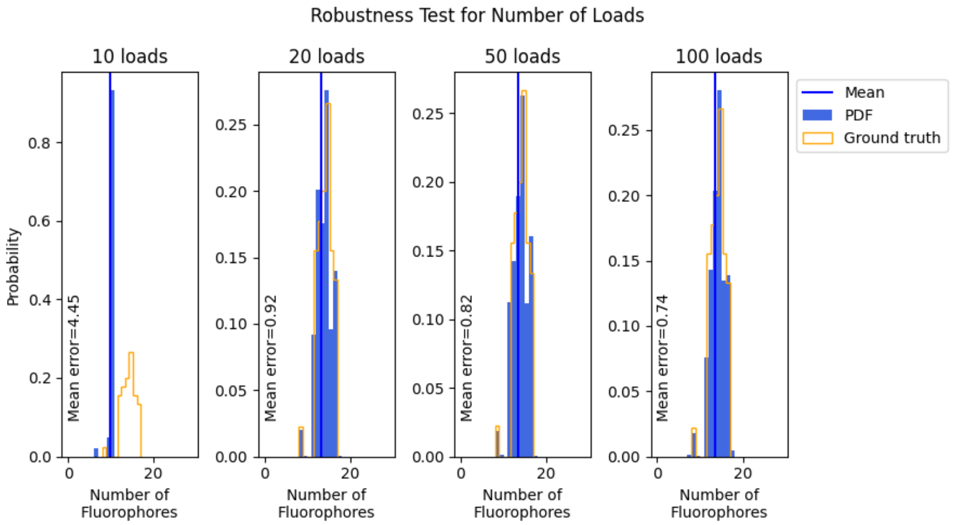

Figure 6: **Inference on fluorophore number from data simulated with varying number of loads.** Each panel shows inference on simulated data using a different number of loads. The mean error is annotated on the bottom left of each panel.

We must make sure that our choice of nonparametric limit in our beta-Bernoulli prior, Eq. (6), does not heavily the number of loads we choose. That is, we want to make sure that if 14 fluorophores are present in an ROI, then our choice of $K = 30$ or $K = 300$ from Eq. (6) is inconsequential. To address this we ran inference on the same simulated data, but with a different number of loads. The results are shown in figure 6.

The top row of figure 6 shows that if we do not provide a sufficient number of loads, then we cannot count the correct number of fluorophores. This is obvious because if the number of fluorophores is determined by how many loads are "on", then when there are not enough loads, we cannot turn on enough loads to account for all the fluorophores. For example, if we provide 9 loads, but there are 14 fluorophores, then even if all 9 loads are "on" we underestimate by 5 fluorophores. Aside from this constraint, we found that once a sufficient number of loads are chosen then the results are not dependent on the number of loads. As seen in the bottom three rows of figure 6, increasing the number of loads has negligible impact on the performance of the sampler.

Therefore, for the rest of this paper we will take for granted that we are free to set the number of loads, K, as we please provided that we set K larger than the maximum expected number of fluorophores. A note however is in order. If we choose too many loads, then the computation becomes expensive and inefficient. As a nice balance between sufficiency and efficiency, we will choose to make the number of loads equal to two times the expected number of fluorophores. In the case that the ground truth number of fluorophores is unknown (like for real data) the best way forward would be to guess a number of loads and if it maxes out (i.e., the number of sampled fluorophores in an ROI is equal to the number of loads) then to double the number of loads and try again.

bioRxiv preprint doi: https://doi.org/10.1101/2020.09.28.317057; this version posted September 23, 2021. The copyright holder for this preprint (which was not certified by peer review) is the author/funder, who has granted bioRxiv a license to display the preprint in perpetuity. It is made available under aCC-BY 4.0 International license.

5.10.2 Varying the number of fluorophores

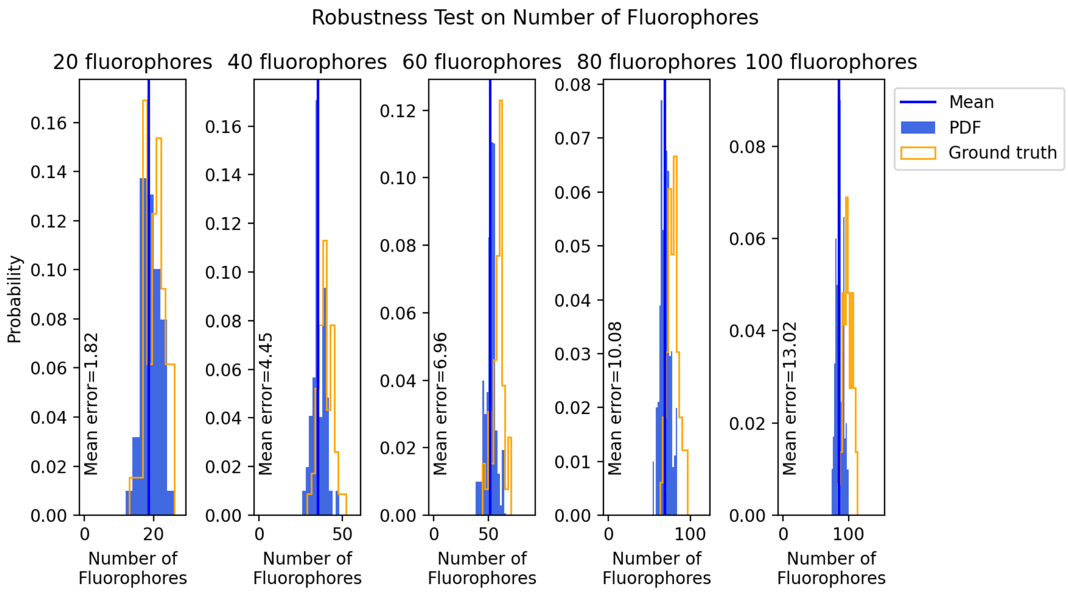

Figure 7: **Inference on fluorophore number from data simulated with varying fluorophore numbers.** Each panel shows inference on data simulated with a different number of fluorophores per ROI.

Now that we have shown that we can accurately count the number of fluorophores in an ROI, the next clear step forward is to see how high we can count. For this, we simulated data using the same base case parameters, but changed the way in which we sample the ground truth number of fluorophores. Here we simulate ten different experiments with 20, 40, ..., 200 fluorophore binding sites and 50% labeling efficiency so that the expected number of fluorophores for each ROI is 10, 20, ..., 100 respectively.

bioRxiv preprint doi: https://doi.org/10.1101/2020.09.28.317057; this version posted September 23, 2021. The copyright holder for this preprint (which was not certified by peer review) is the author/funder, who has granted bioRxiv a license to display the preprint in perpetuity. It is made available under aCC-BY 4.0 International license.

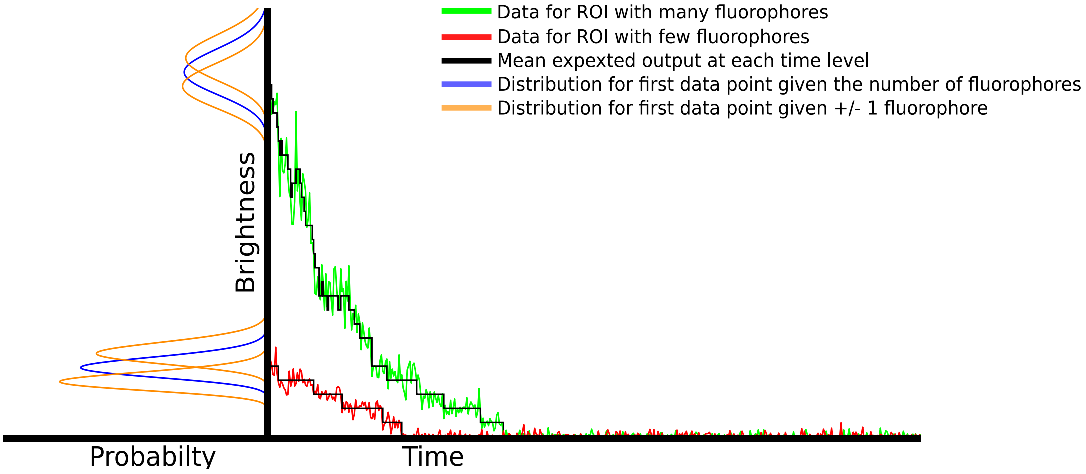

Figure 8: **As the number of active fluorophores increases, photon shot noise, amplified by camera noise, becomes an essential modeling component.** On the right we see two brightness time traces for an ROI. The ROI giving rise to the red trace contains 5 fluorophores and the ROI giving rise to the green trace contains 26 fluorophores. The black line through each trace represents the expected mean brightness at each time level based on the background brightness, fluorophore brightness, and number of active fluorophores at the time level. On the left we see the probability distribution for the initial brightness of each ROI (blue) as well as the probability distribution for the initial brightness if the ROI contained one more or fewer fluorophores (orange). We notice that: 1) for the red trace, the measured brightness matches closely with the mean, whereas the green trace fluctuates wildly; 2) the probability for the initial brightness is sharply peaked for the red trace which allows us to easily tell how many fluorophores are initially active. By contrast, the probability for the initial brightness is much more spread out for the green trace and overlaps greatly with the distributions for one more and one fewer fluorophores.

As seen in figure 7, on simulated data for the parameters chosen, the learned distribution of fluorophores overlaps with the ground truth for all simulations. There is a slight underestimation, in which the sampler misses about 1 fluorophore out of every ten, that becomes more noticeable as the number of simulated fluorophores increases. The source of this underestimation is the prior on the number of loads(equation 6), which favors samples from the posterior that use fewer fluorophores to explain the data. At the beginning of the brightness trace, where the width of the brightness levels is much wider than the separation between brightness levels (figure 8), the sampler may "choose" to interpret steps as noise fluctuations, thus missing fluorophores. This underestimation percentage is consistent across number of fluorophores used for the simulations (we have similar error percentage when there are 20 ground truth fluorophores as when there are 100 fluorophores) and thus does not negate that we can count high number of fluorophores.

bioRxiv preprint doi: https://doi.org/10.1101/2020.09.28.317057; this version posted September 23, 2021. The copyright holder for this preprint (which was not certified by peer review) is the author/funder, who has granted bioRxiv a license to display the preprint in perpetuity. It is made available under aCC-BY 4.0 International license.

5.10.3 Varying the number of states

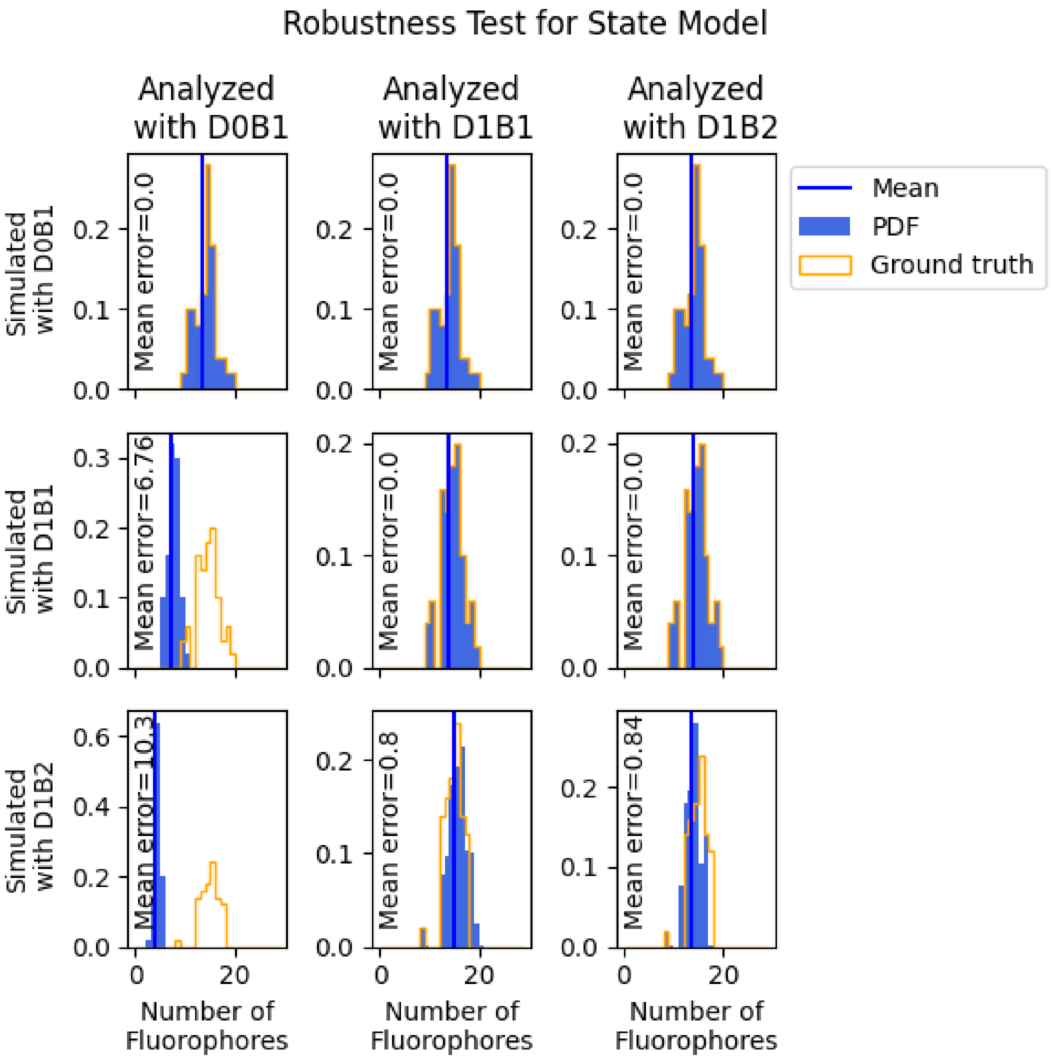

Figure 9: **Inference on data simulated with different number of states.** Here for each row we generate simulated data using a different state model for the forward model. Each column shows the inference on that data set using a different state model for the inverse model.

We note that while our model is nonparametric in the number of fluorophores in an ROI (i.e., that the number of fluorophores in an ROI is not a fixed quantity but inferred), our model is parametric in the number of fluorophore states. That is, we must pre-specify the number of fluorophore states. It is difficult to be both nonparametric in the number of fluorophores and the number of states (as then, trivially, each transition could be considered to visit a new state of the fluorophore). In the previous sections, we have taken for granted that we knew the correct number of fluorophore states. We modeled that the fluorophores have two separate bright states, a dark state, and a photobleached state. Here we explore the photophysical state space further using different numbers of bright states. For conciseness, in this section we will refer to these models using a naming convention that starts with 'D' followed by the number of dark states and then 'B' followed by the number of bright states: 1) D0B1, a model with no dark state and one bright state (i.e., the fluorophore is either bright or photobleached); 2) D1B1, a model with one dark state one bright state; and 3) D1B2, a model with one dark state and two bright states. In principle

bioRxiv preprint doi: https://doi.org/10.1101/2020.09.28.317057; this version posted September 23, 2021. The copyright holder for this preprint (which was not certified by peer review) is the author/funder, who has granted bioRxiv a license to display the preprint in perpetuity. It is made available under aCC-BY 4.0 International license.

we could also explore more bright or dark states, but the combinatorics on the possible arrangements on fluorophore states adds computational burden. We note that the base case set of parameters uses a D1B2 model and the model with only two states, D0B1, is the same model as the one used in Garry et al. [25].

We simulated data using each of the different state models and then analyzed each simulated data set using each of the different state models. Results are shown in figure 9. Starting left to right, we first notice that the D0B1 model does well at counting fluorophores from data generated using the D0B1 model (figure 9 top left). On the other hand, the inference severely underestimates the number of fluorophores for the data generated using the D1B1 model (figure 9 middle left) and the D1B2 model (figure 9 bottom left). This is because, lacking a dark state, no possible fluorophore state trajectory under the D0B1 model can account for blinks where the brightness of the ROI goes down and then comes back up. Thus the only way for the method to account for blinks is to increase the variance (by increasing the background brightness, μ_B) to a level where a sudden drop in brightness is explained purely by noise. This in turn makes photobleaching steps the same size as noise fluctuations (because the drop in brightness from a blink is the same as a drop in brightness from a photobleaching event), thus the method "misses" photobleaching steps and most brightness drops are explained purely by the exponentially decaying background.

The remaining models, D1B1 and D1B2, provide good matches to the ground truth number of fluorophores for the data with no dark state (figure 9 top row). In this case the dark state is never visited despite being available. Similarly both models are able to exactly learn the ground truth number of fluorophores in the data sets with only one bright state. Again, in this case the two bright state inverse model will simply rarely visit one of its available bright states. For the data generated using two bright states, both the single bright state model, D1B1, and the two bright state model, D1B2, both infer a distribution of fluorophore numbers with similar accuracy. As the two bright states are very close to each other in brightness, the D1B1 model is able to find a brightness that averages the two together. For this reason, we say that our inference scheme is robust to bright state number. The advantage to using the two bright state model, D1B2, is that the inferred brightness traces will learn bright state transitions kinetics.

5.11 Additional comparison

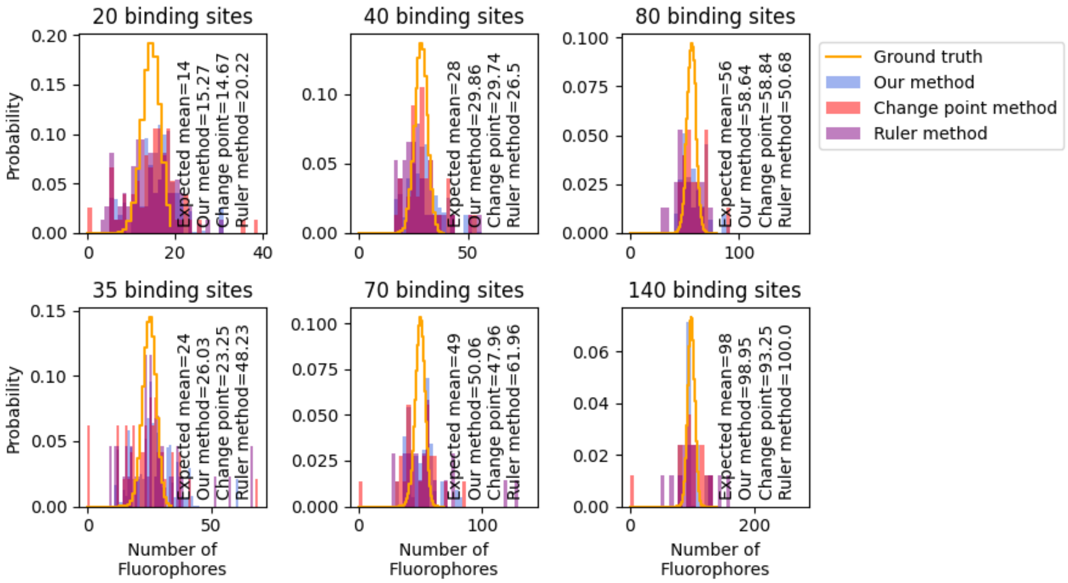

Figure 10: **Comparison against other methods on real data.** Here we compare our method against a change point method and the ruler methods on real data. Each panel shows the inference on a data set with a different number of fluorophores. We plot the inferred distribution for the number of fluorophores for each method in a different color. On the bottom right of each panel we annotate the mean expected number of fluorophores as well as the learned mean expected number of fluorophores.

bioRxiv preprint doi: https://doi.org/10.1101/2020.09.28.317057; this version posted September 23, 2021. The copyright holder for this preprint (which was not certified by peer review) is the author/funder, who has granted bioRxiv a license to display the preprint in perpetuity. It is made available under aCC-BY 4.0 International license.

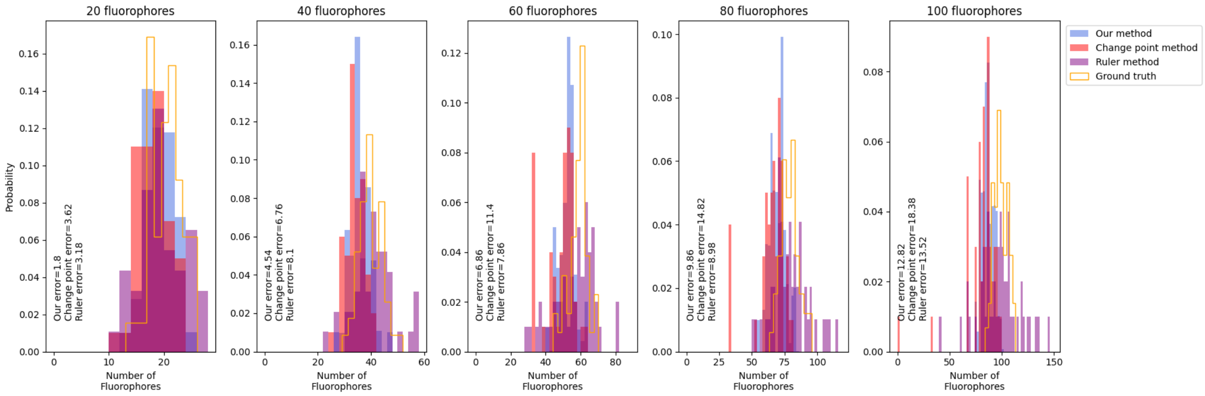

Figure 11: **Additional comparison against other methods on simulated data**. Here we compare our method against a change point method [15] and the ruler methods on real data. Each panel shows the inference on a data set simulated with a different number of fluorophores. We plot the inferred distribution for the number of fluorophores for each method in a different color. On the bottom left of each panel we annotate the mean error.

Here we plot additional figures comparing our method to the change point detection algorithm laid out in Tsekouras et al. [15] and the ruler method[12, 13].

In figure 11 we plot the learned distribution for the number of fluorophores for each method as well as the ground truth distribution for the number of fluorophores. On the bottom left of each panel we show the mean error of each method, that is the average difference between the predicted number of fluorophores and the ground truth number of fluorophores for each ROI. Our method provides the least error for all data sets except the 80 fluorophore data set in which the ruler method has less error. This is likely a statistical artifact in which the noise fluctuations at the beginning of the trace and end of the last photobleaching step made estimating the fluorophore brightness and initial brightness easier. Note on the other hand that because such statistical deviations are rare, for every other data set our method is more accurate than the ruler method for this point statistic alone (determining fluorophore counts).

In figure 10 we plot the learned distribution for the number of fluorophores for each method as well as the ground truth expected distribution for the number of fluorophores. As there is no known ground truth, we cannot compare errors. Instead we compare the learned average number of fluorophores against the best guess for the expected average number of fluorophores given the factory estimated binding rate. We note that the ruler method occasionally predicts very high number of fluorophores (200+) when the fluorophore brightness is underestimated due to blinking or random spikes.

We can use our obtained mean number of fluorophores to validate the 70% binding efficiency provided to us by the manufacturer. We do this by dividing the learned mean number of fluorophores by the number of binding sites. Doing this we estimate 76% binding efficiency from our 20 binding site data and we estimate 74% binding efficiency from our 35 binding site data. Both of these estimates are within 10% of the provided estimate.

5.12 Full posteriors

Here we plot full posterior samples for each unknown quantity from the analysis on each data set. We do this in two figures for each experiment. For the first part we plot the Gibbs sample trace showing the values of each of our parameters as a function of iteration. The top panel of each figure shows the log probability at each iteration. The second panel shows the brightness of the bright states at each iteration. The third panel shows the lifetime of the bright states at each iteration. The final panel shows the learned average number of fluorophores at each iteration, that is, we learn the number of fluorophores in each ROI and then average them. For simulated data sets we plot the ground truth values as a horizontal line. As there are two bright states, the middle two panels will have two Gibbs sample traces (there are also two ground truth values, but they are very close together). For the second figure we show a scatter plot of the covariance of the posterior. For this plot, each row and column represent a different variable (we simplify

bioRxiv preprint doi: https://doi.org/10.1101/2020.09.28.317057; this version posted September 23, 2021. The copyright holder for this preprint (which was not certified by peer review) is the author/funder, who has granted bioRxiv a license to display the preprint in perpetuity. It is made available under aCC-BY 4.0 International license.

by only showing the brightness of the brightest state, the lifetime of the brightest state, and the mean number of fluorophores per ROI). Each panel i, j shows there covariance between variable i and variable j (where, again, i and j will be the brightness of the brightest state, the lifetime of the brightest state, and the mean number of fluorophores per ROI) and each panel along the diagonal shows the histogrammed posterior of variable i.

The input parameters used for analysis are the same for each data set and correspond to the default inputs on the provided code. We reproduce those default values below. Let u_r be the average of the last 100 data points of ROI r which is used for estimating the background brightness.

f	fluorophore brightness guess	10000
G	gain*	22
γ	hyperparameter on loads	.1
ϕ_{μ_A}	scale parameter for fluorophore brightness	100
ψ_{μ_A}	shape parameter for fluorophore brightness	$f/(G \times \phi_{\mu_A})$
$\phi^r_{\mu_B}$	scale parameter for background brightness	10
$\psi^r_{\mu_B}$	shape parameter for background brightness	$u_r/\phi^r_{\mu_B}$
$\boldsymbol{\pi}$	concentration parameter for transitions	$\begin{bmatrix} 100 & 1 & 1 & 0 \\ 1 & 100 & 1 & 1 \\ 1 & 1 & 100 & 1 \\ 0 & 0 & 0 & 1 \end{bmatrix}$
$\boldsymbol{\pi_0}$	concentration parameter for initial state	$\begin{bmatrix} 1 & 1 & 1 & 0 \end{bmatrix}$

*Gain is $G = 2200$ for the experiment in figure 4 bottom row.

For all experiments we perform one MCMC chain with a set random seed. We run our Gibbs sampler for 20,000 iterations or 1 week, whichever comes first, then eliminate the burn in phase by selecting only the samples where the variables and the log posterior have stabilized. Our heuristic metric for stablization is when the slope of all samples with respect to Gibbs sample iteration is less than one percent of the mean.

Note that the state lifetimes are calculated from the transition learned transition matrix as a post processing step via $\tau_k = \mathrm{dt}/(1 - \pi_{k,k})$ where τ_k is the lifetime of state k and $\pi_{k,k}$ is the self transition probability of state k. Under this transformation, small displacements in $\pi_{k,k}$ can cause large displacements in τ_k. For this reason, the learned bright state lifetimes can be off by as much as a factor of 2 even if the learned transition rate is only off by 10% or less.

bioRxiv preprint doi: https://doi.org/10.1101/2020.09.28.317057; this version posted September 23, 2021. The copyright holder for this preprint (which was not certified by peer review) is the author/funder, who has granted bioRxiv a license to display the preprint in perpetuity. It is made available under aCC-BY 4.0 International license.

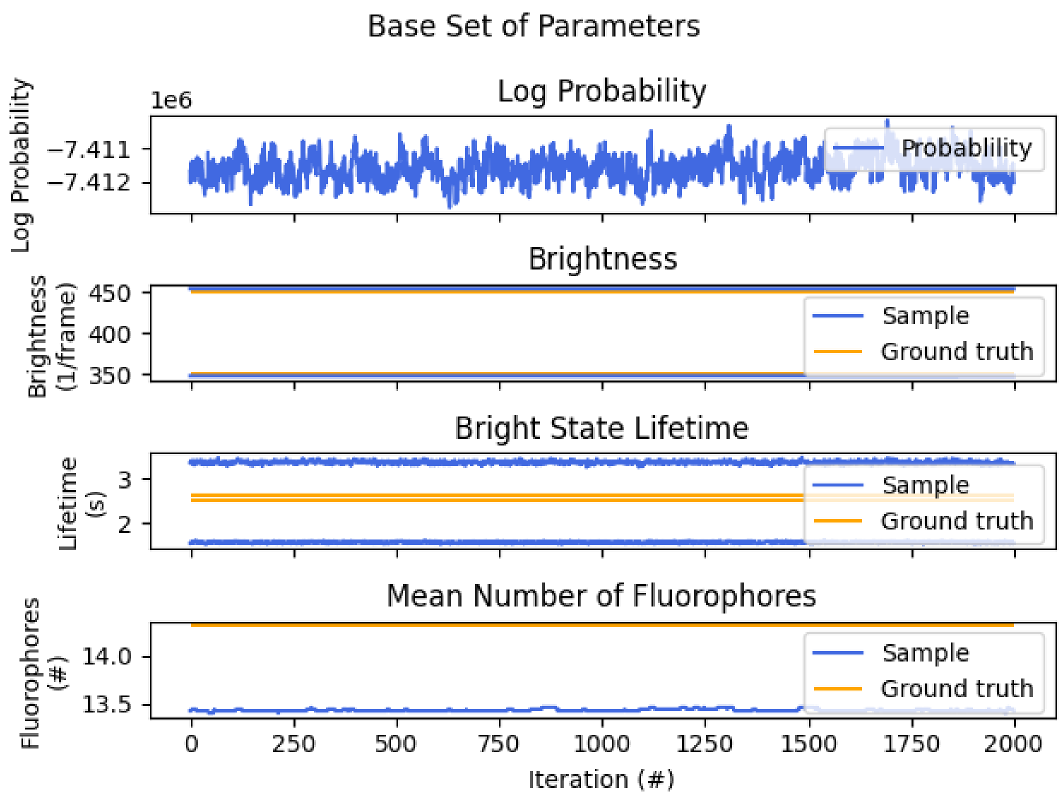

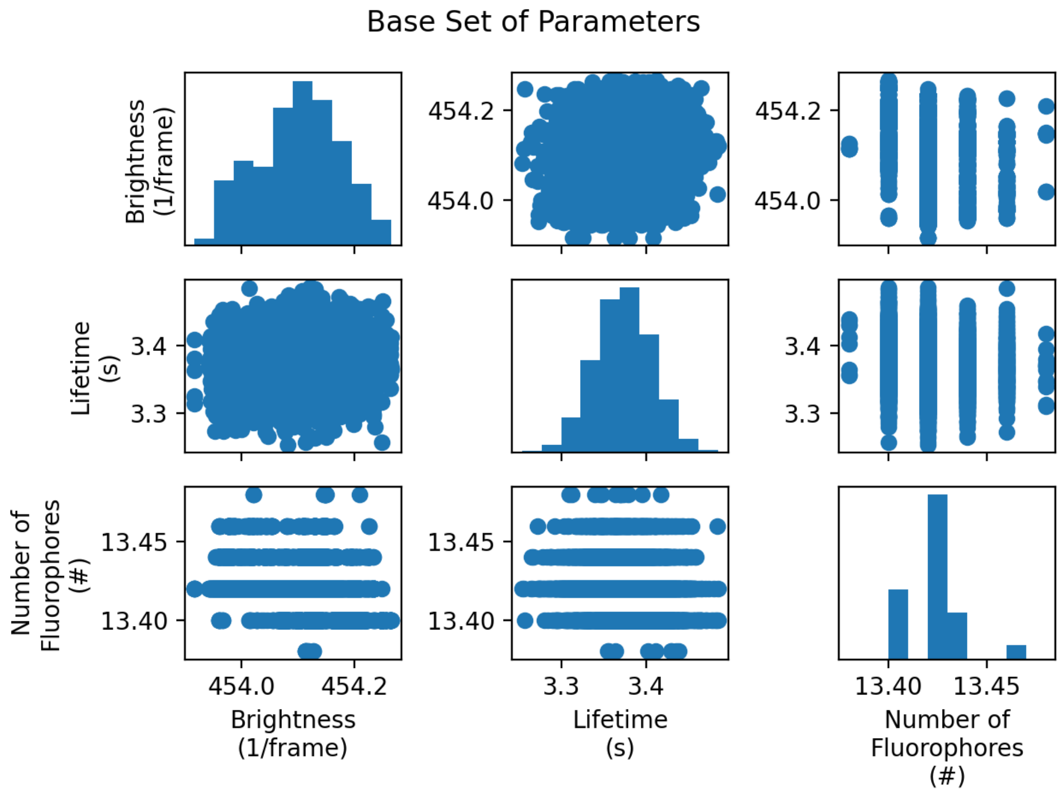

bioRxiv preprint doi: https://doi.org/10.1101/2020.09.28.317057; this version posted September 23, 2021. The copyright holder for this preprint (which was not certified by peer review) is the author/funder, who has granted bioRxiv a license to display the preprint in perpetuity. It is made available under aCC-BY 4.0 International license.

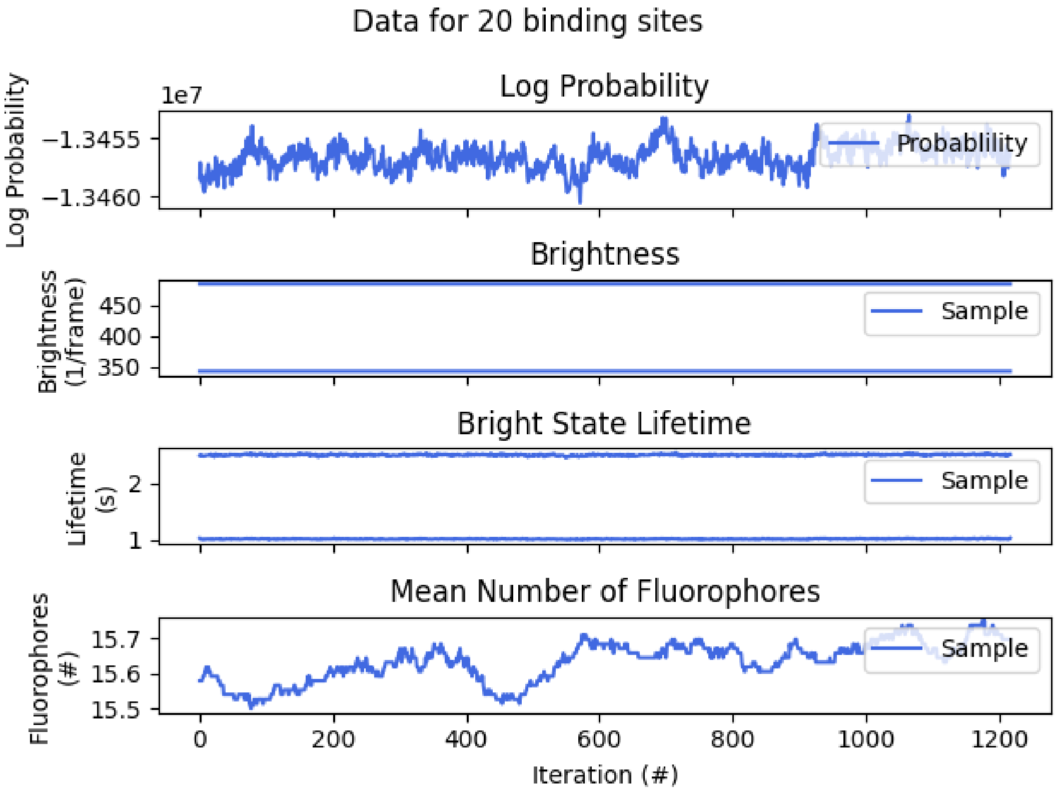

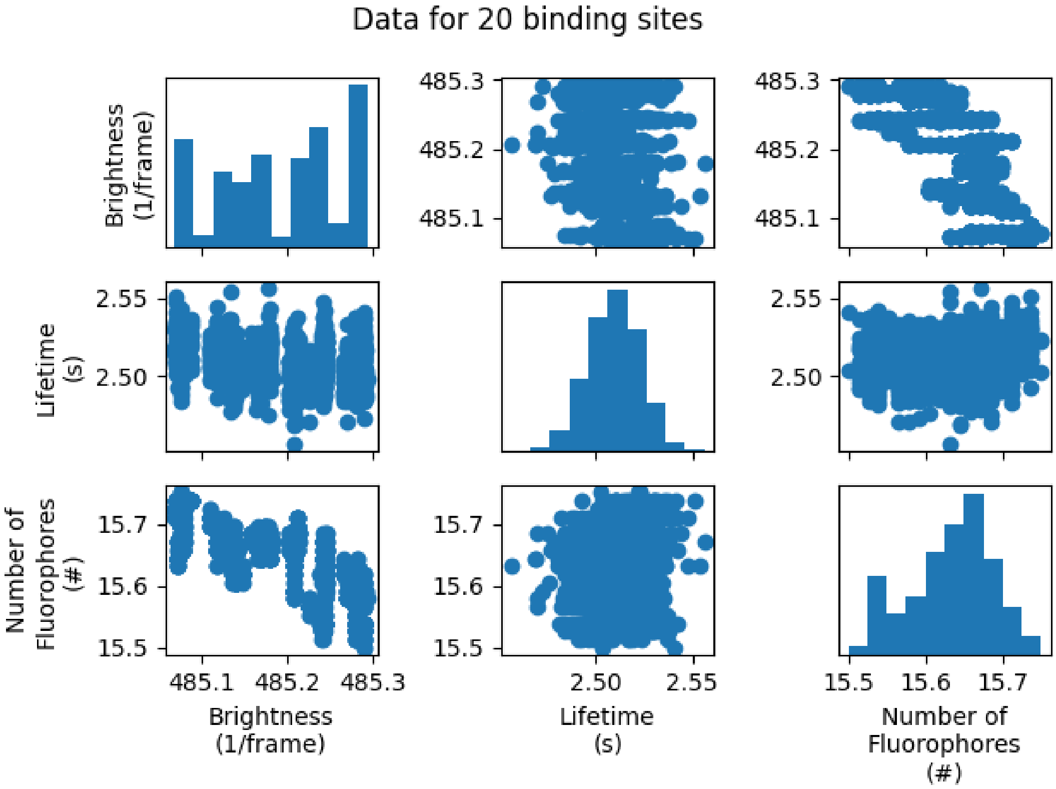

bioRxiv preprint doi: https://doi.org/10.1101/2020.09.28.317057; this version posted September 23, 2021. The copyright holder for this preprint (which was not certified by peer review) is the author/funder, who has granted bioRxiv a license to display the preprint in perpetuity. It is made available under aCC-BY 4.0 International license.

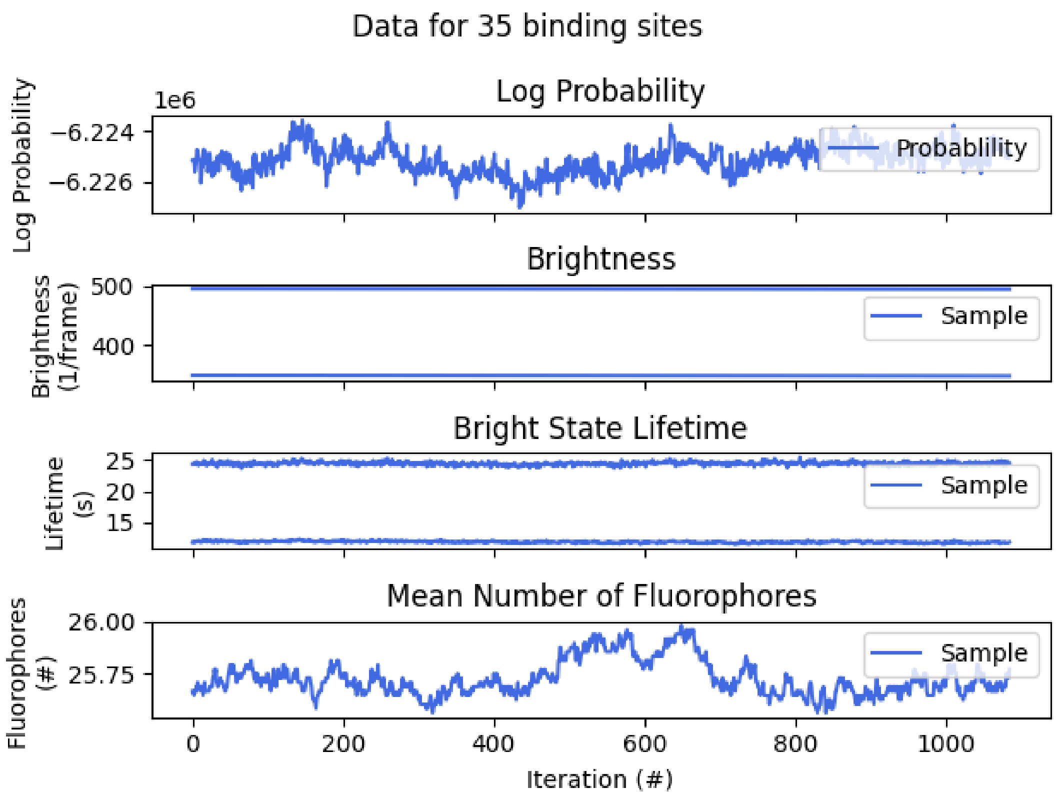

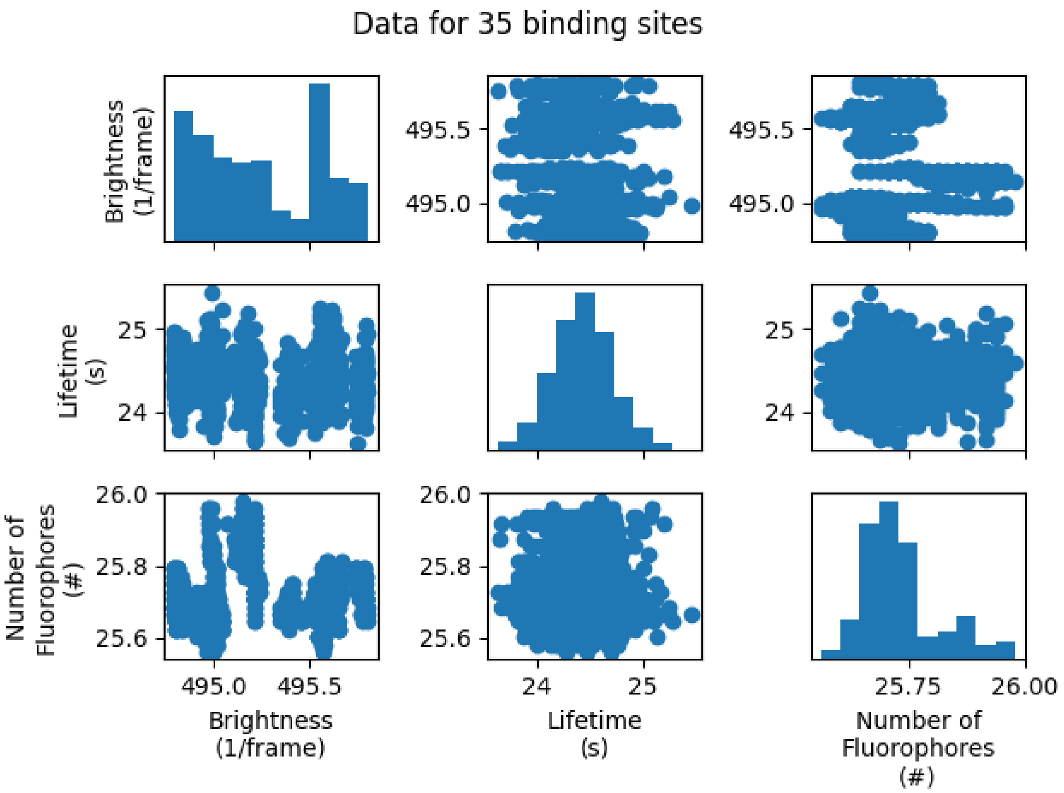

bioRxiv preprint doi: https://doi.org/10.1101/2020.09.28.317057; this version posted September 23, 2021. The copyright holder for this preprint (which was not certified by peer review) is the author/funder, who has granted bioRxiv a license to display the preprint in perpetuity. It is made available under aCC-BY 4.0 International license.

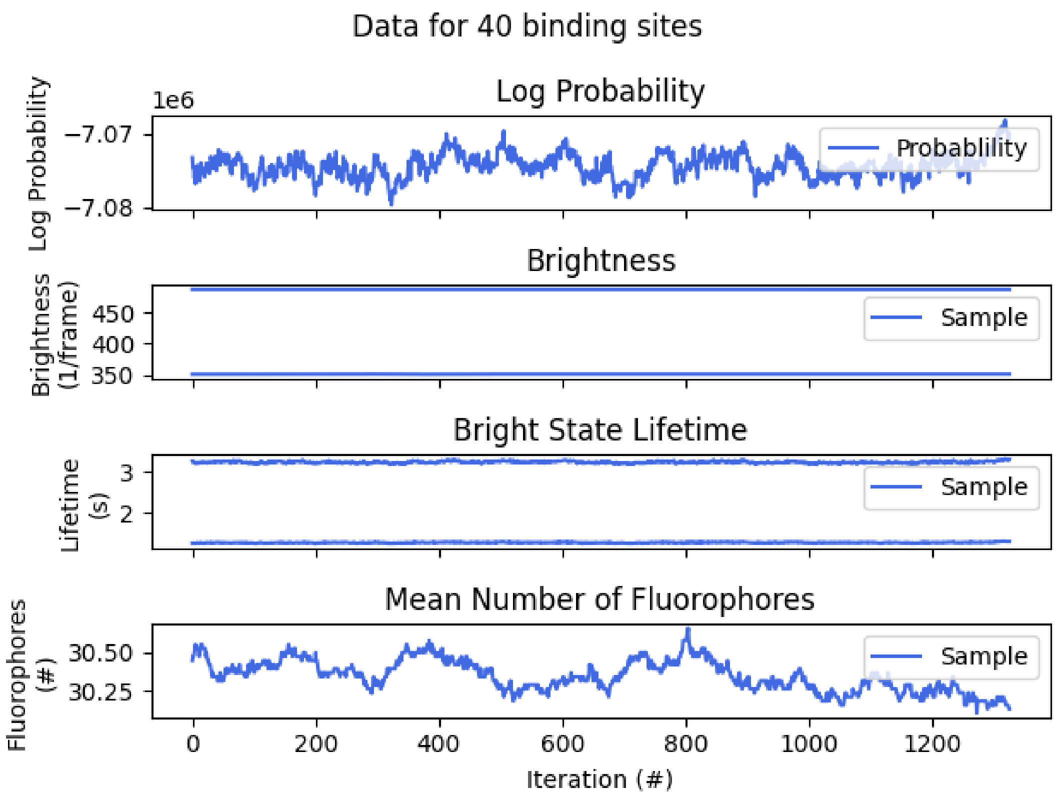

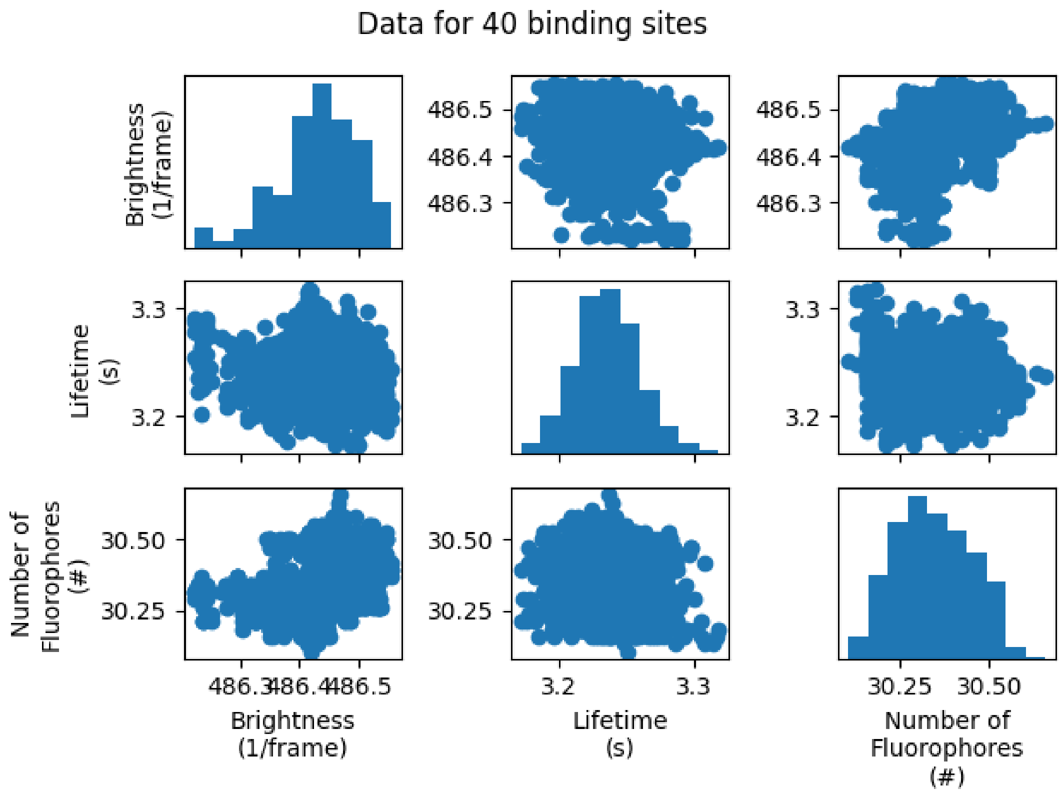

bioRxiv preprint doi: https://doi.org/10.1101/2020.09.28.317057; this version posted September 23, 2021. The copyright holder for this preprint (which was not certified by peer review) is the author/funder, who has granted bioRxiv a license to display the preprint in perpetuity. It is made available under aCC-BY 4.0 International license.

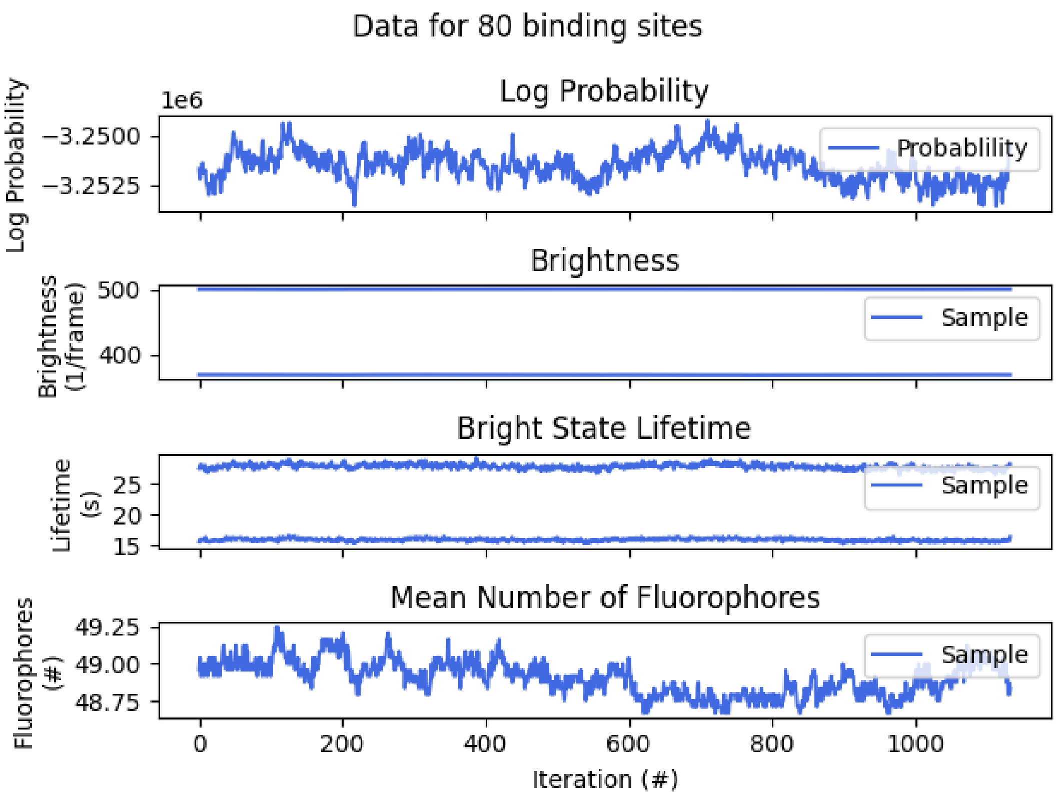

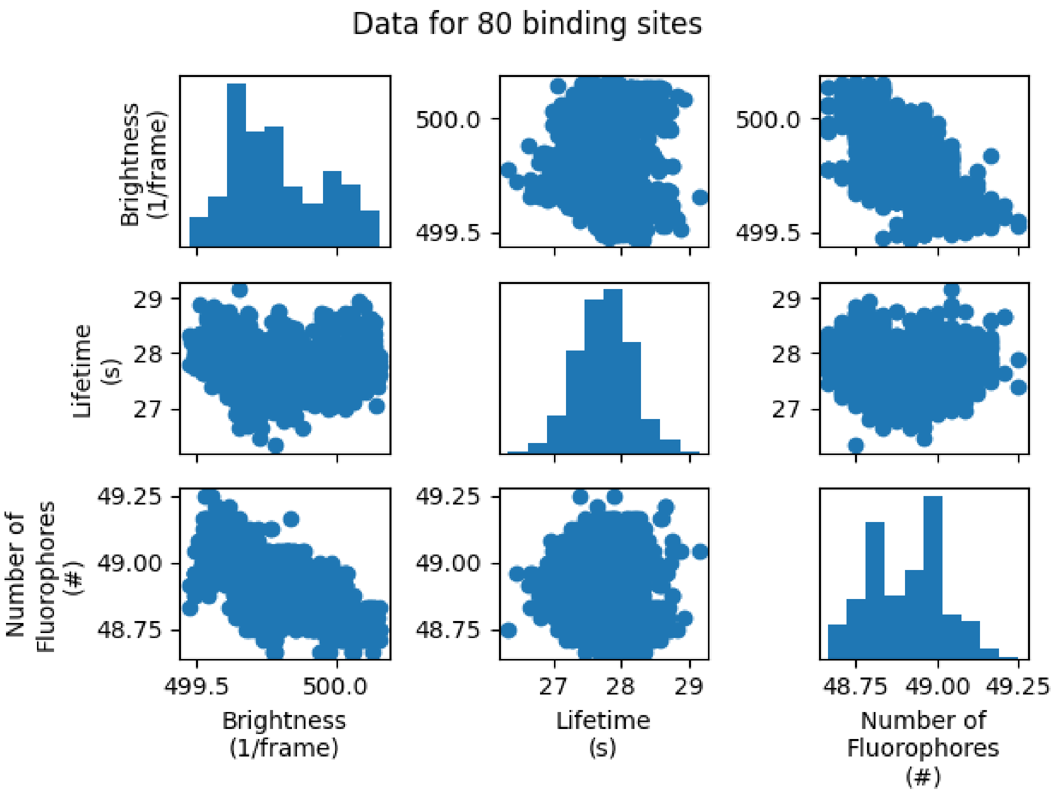

bioRxiv preprint doi: https://doi.org/10.1101/2020.09.28.317057; this version posted September 23, 2021. The copyright holder for this preprint (which was not certified by peer review) is the author/funder, who has granted bioRxiv a license to display the preprint in perpetuity. It is made available under aCC-BY 4.0 International license.

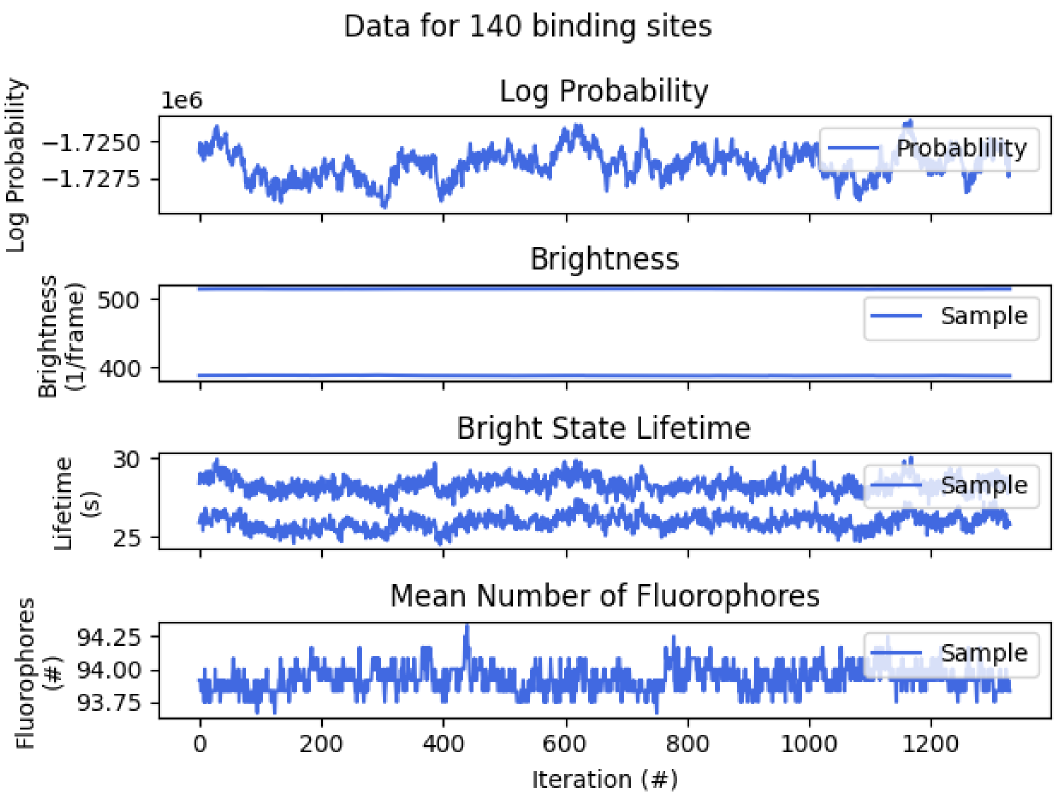

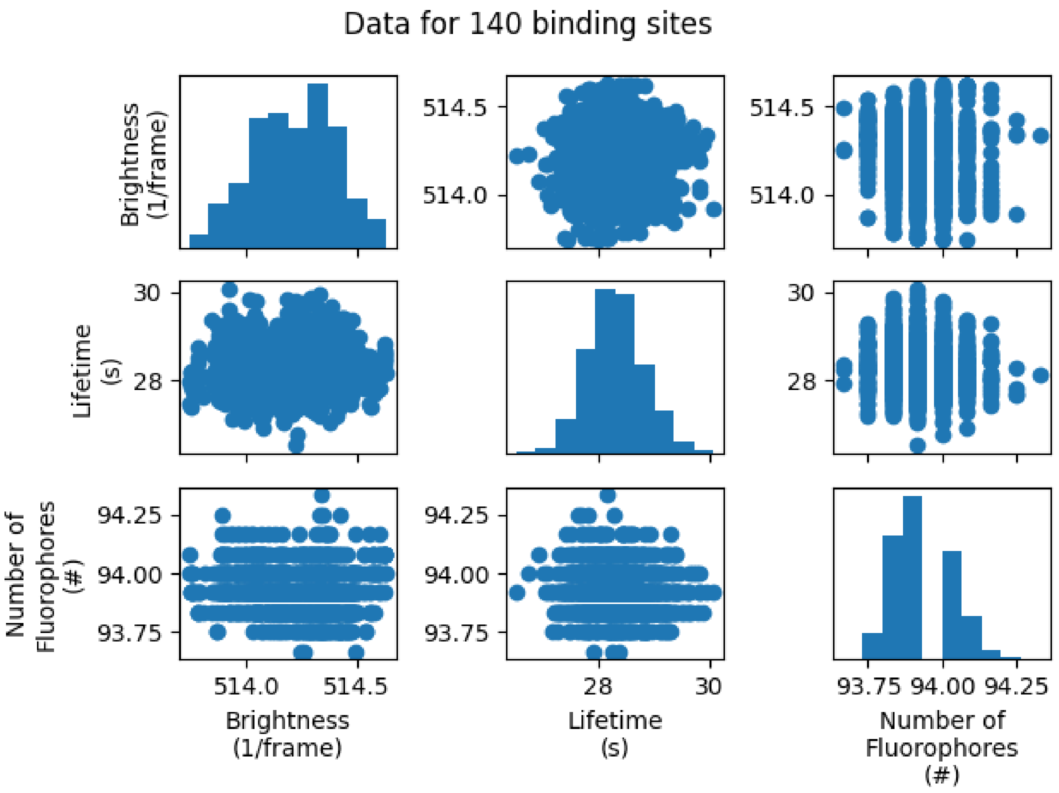

bioRxiv preprint doi: https://doi.org/10.1101/2020.09.28.317057; this version posted September 23, 2021. The copyright holder for this preprint (which was not certified by peer review) is the author/funder, who has granted bioRxiv a license to display the preprint in perpetuity. It is made available under aCC-BY 4.0 International license.

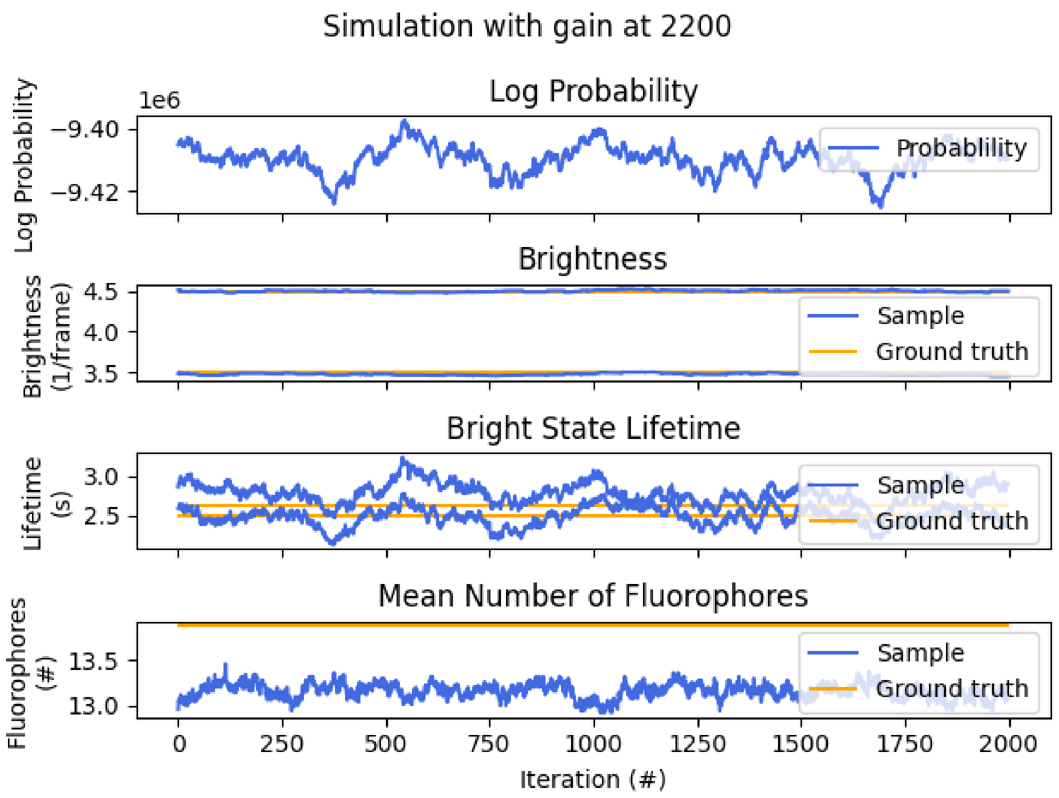

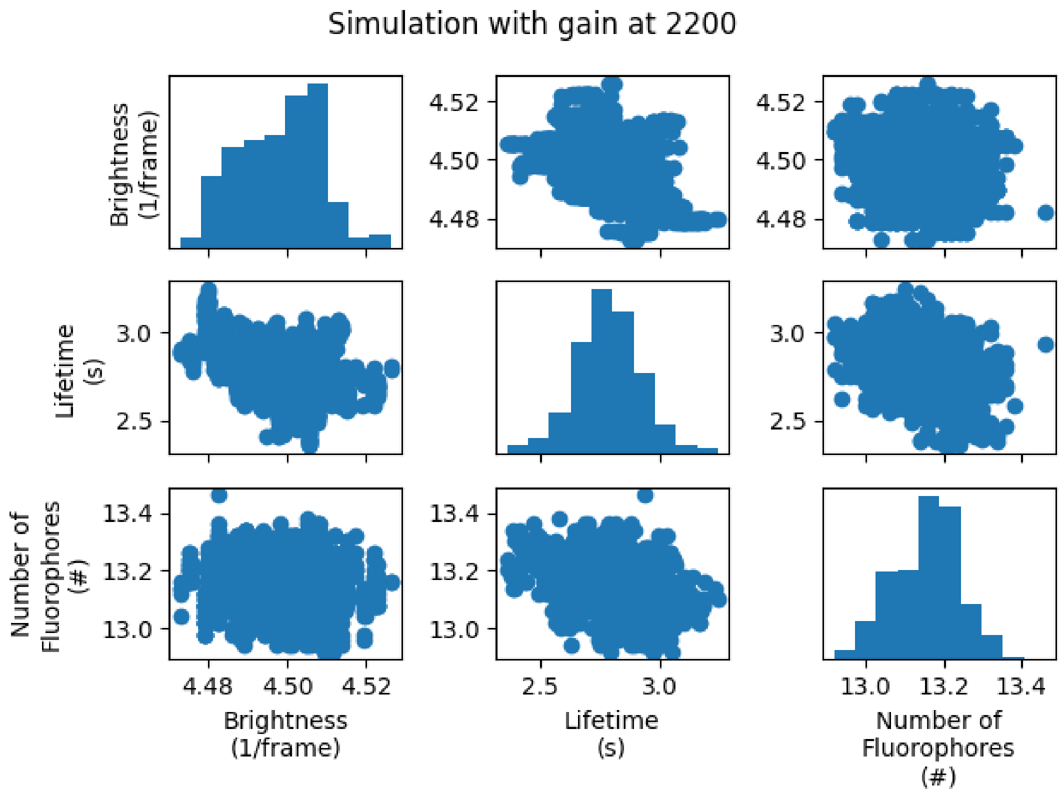

bioRxiv preprint doi: https://doi.org/10.1101/2020.09.28.317057; this version posted September 23, 2021. The copyright holder for this preprint (which was not certified by peer review) is the author/funder, who has granted bioRxiv a license to display the preprint in perpetuity. It is made available under aCC-BY 4.0 International license.

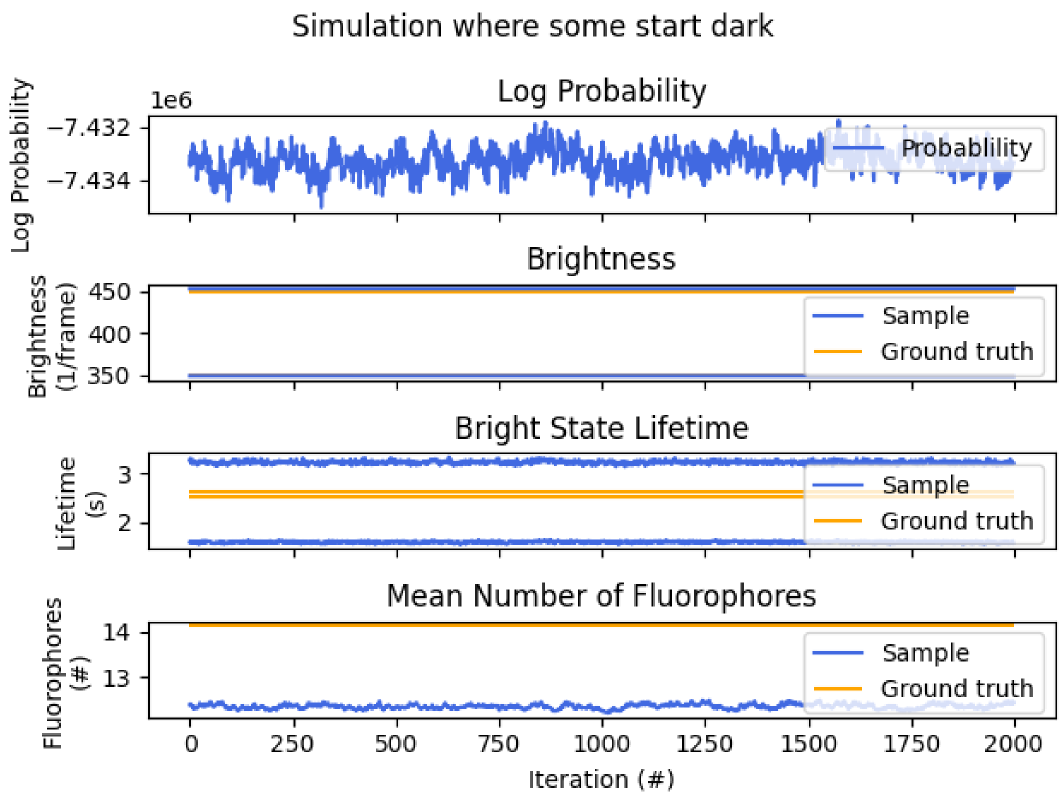

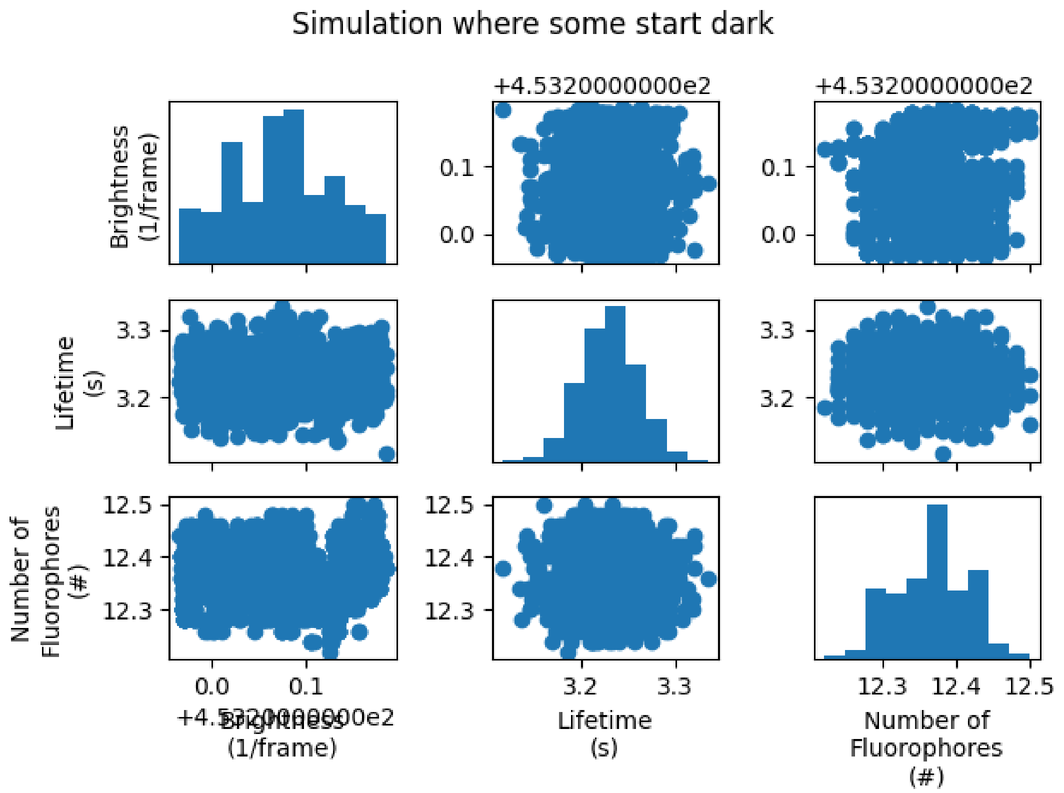

bioRxiv preprint doi: https://doi.org/10.1101/2020.09.28.317057; this version posted September 23, 2021. The copyright holder for this preprint (which was not certified by peer review) is the author/funder, who has granted bioRxiv a license to display the preprint in perpetuity. It is made available under aCC-BY 4.0 International license.

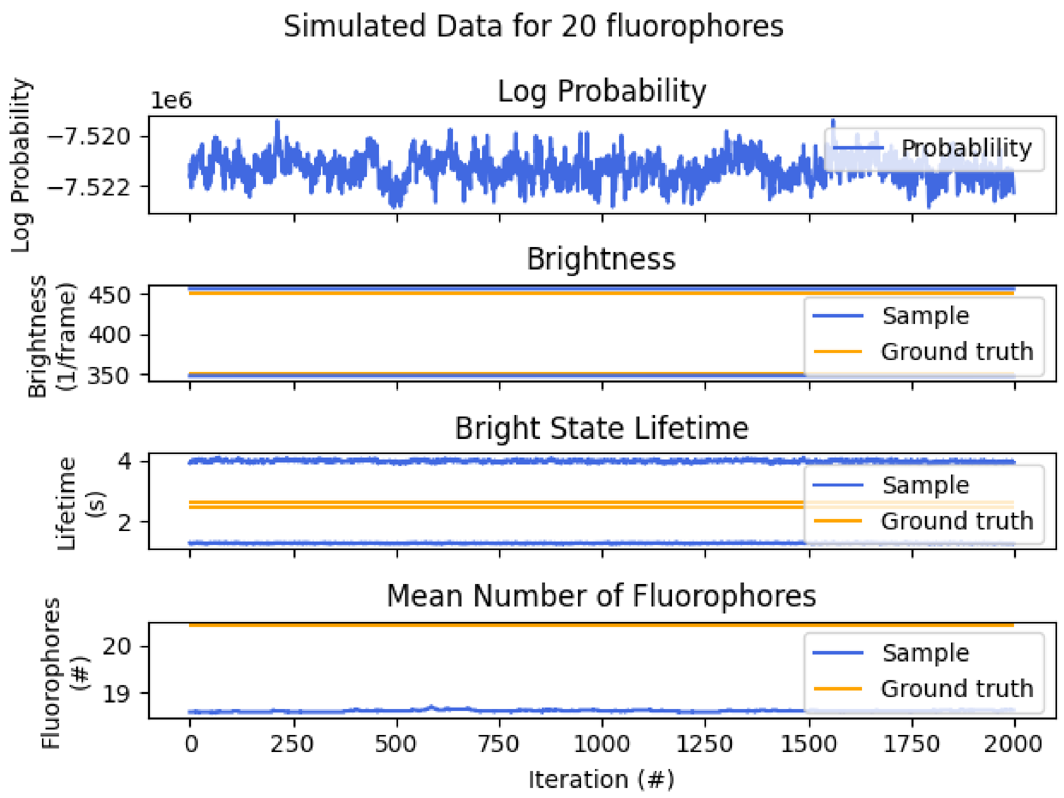

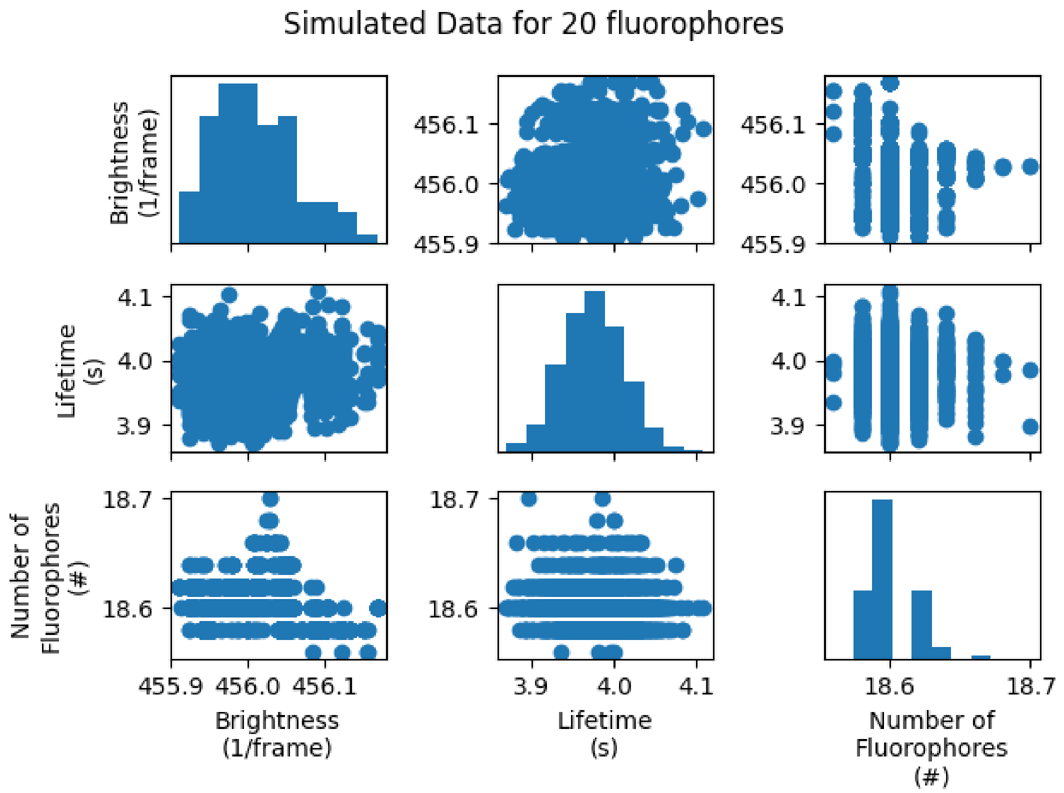

bioRxiv preprint doi: https://doi.org/10.1101/2020.09.28.317057; this version posted September 23, 2021. The copyright holder for this preprint (which was not certified by peer review) is the author/funder, who has granted bioRxiv a license to display the preprint in perpetuity. It is made available under aCC-BY 4.0 International license.

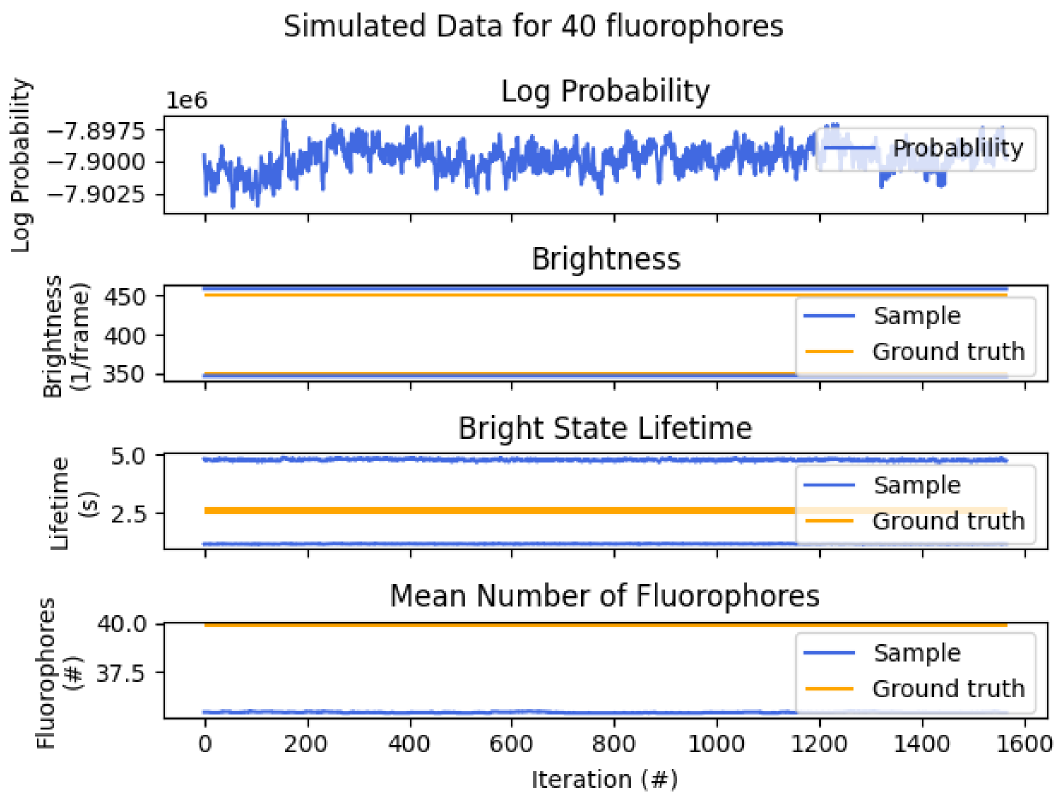

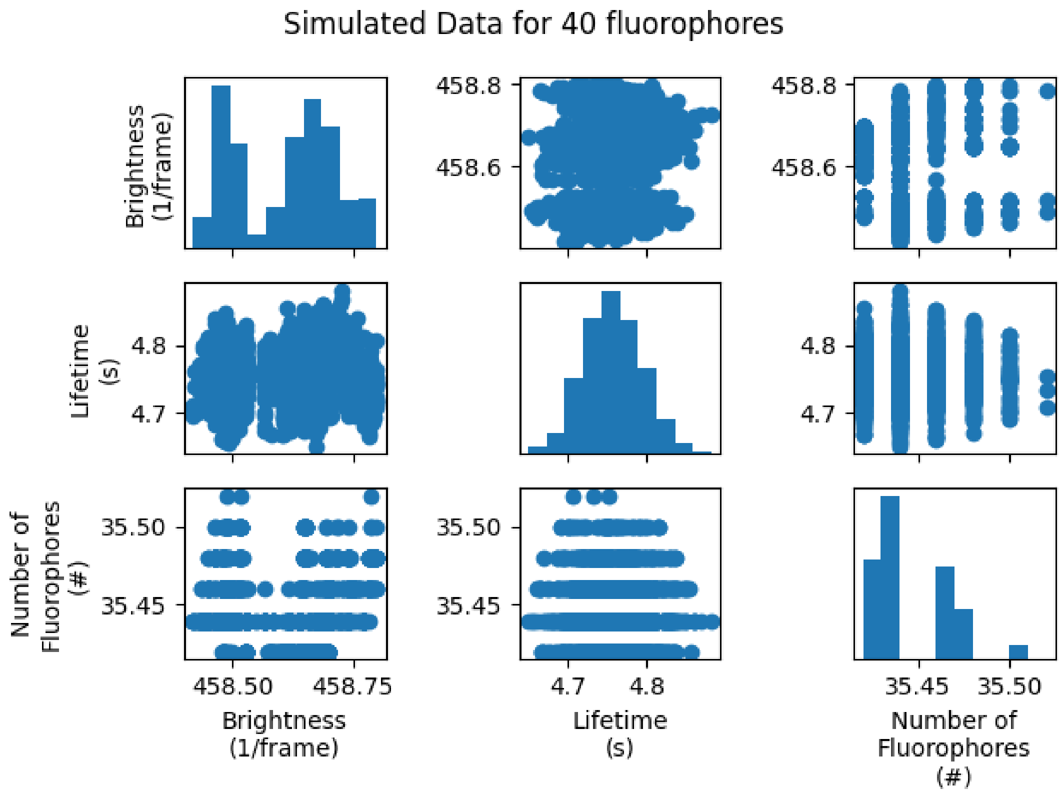

bioRxiv preprint doi: https://doi.org/10.1101/2020.09.28.317057; this version posted September 23, 2021. The copyright holder for this preprint (which was not certified by peer review) is the author/funder, who has granted bioRxiv a license to display the preprint in perpetuity. It is made available under aCC-BY 4.0 International license.

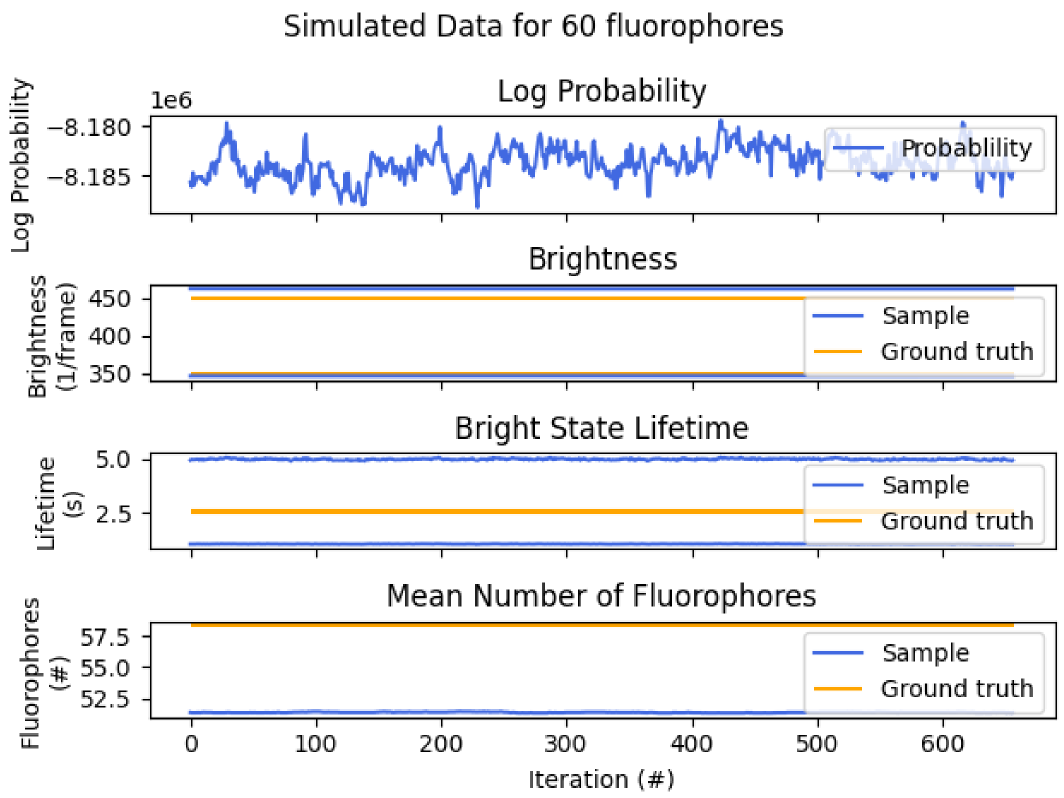

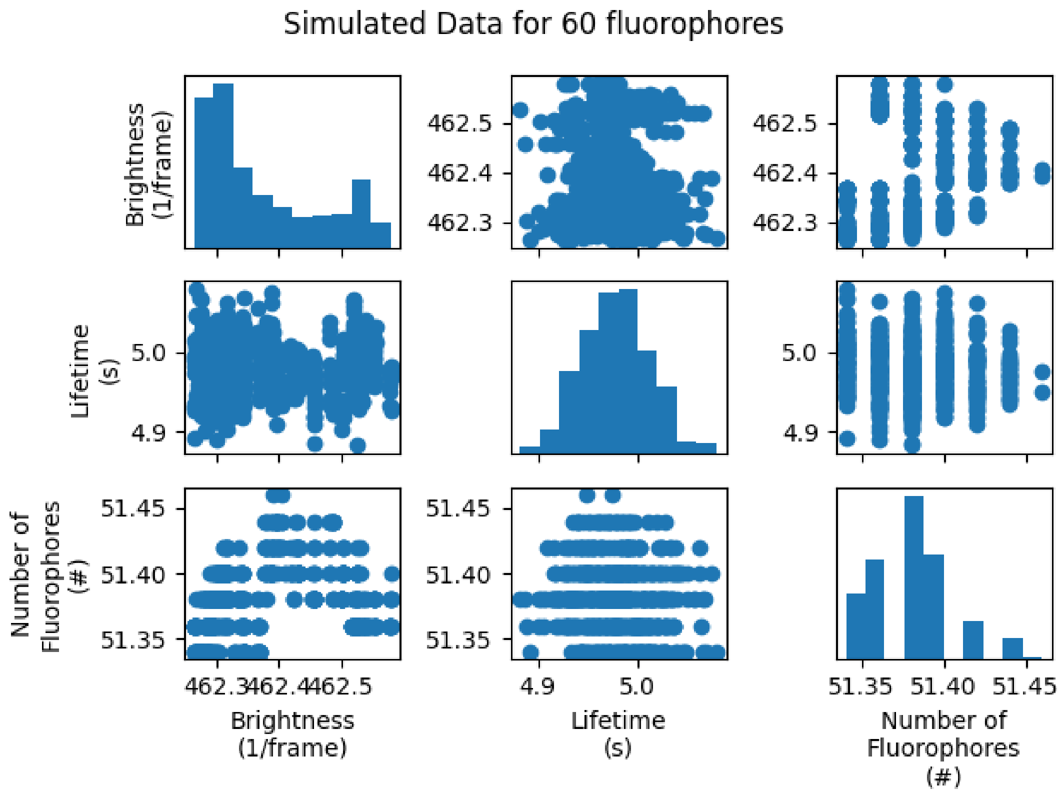

bioRxiv preprint doi: https://doi.org/10.1101/2020.09.28.317057; this version posted September 23, 2021. The copyright holder for this preprint (which was not certified by peer review) is the author/funder, who has granted bioRxiv a license to display the preprint in perpetuity. It is made available under aCC-BY 4.0 International license.

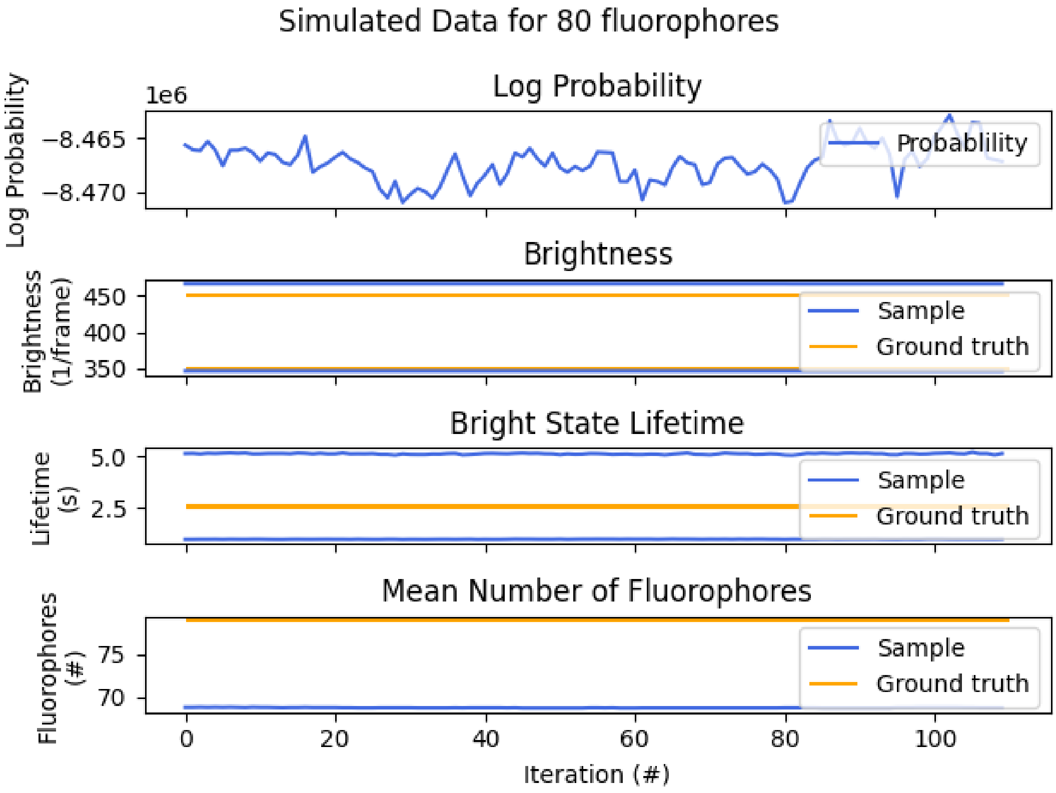

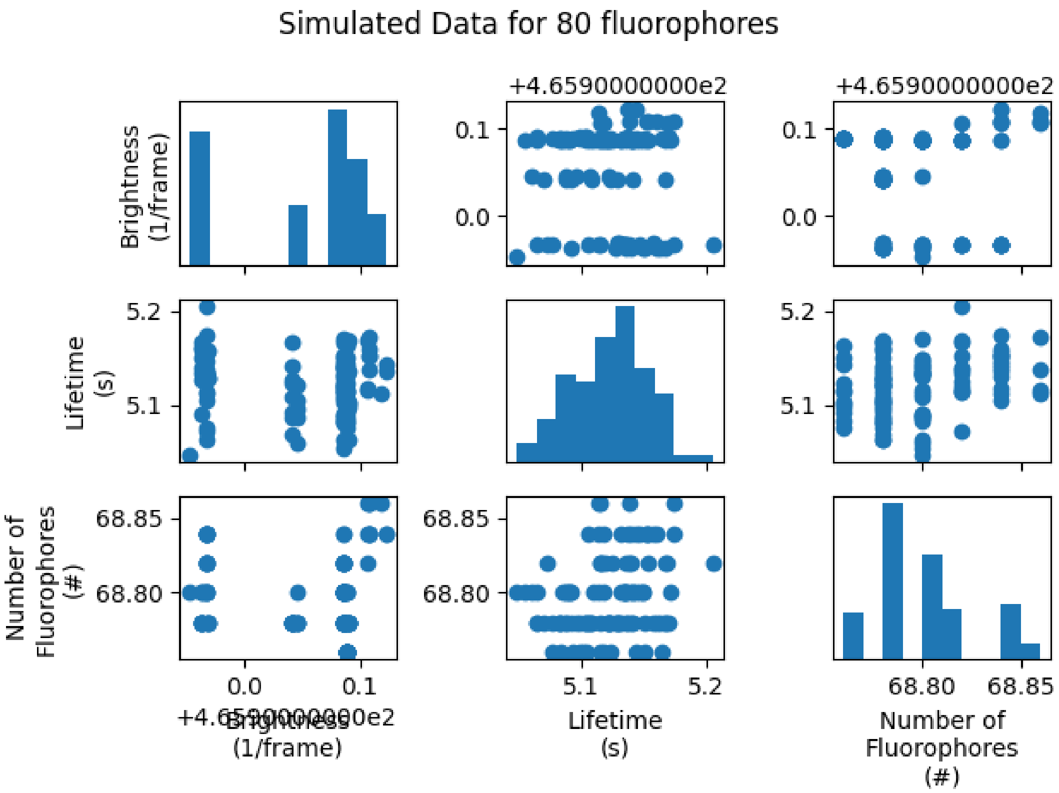

bioRxiv preprint doi: https://doi.org/10.1101/2020.09.28.317057; this version posted September 23, 2021. The copyright holder for this preprint (which was not certified by peer review) is the author/funder, who has granted bioRxiv a license to display the preprint in perpetuity. It is made available under aCC-BY 4.0 International license.

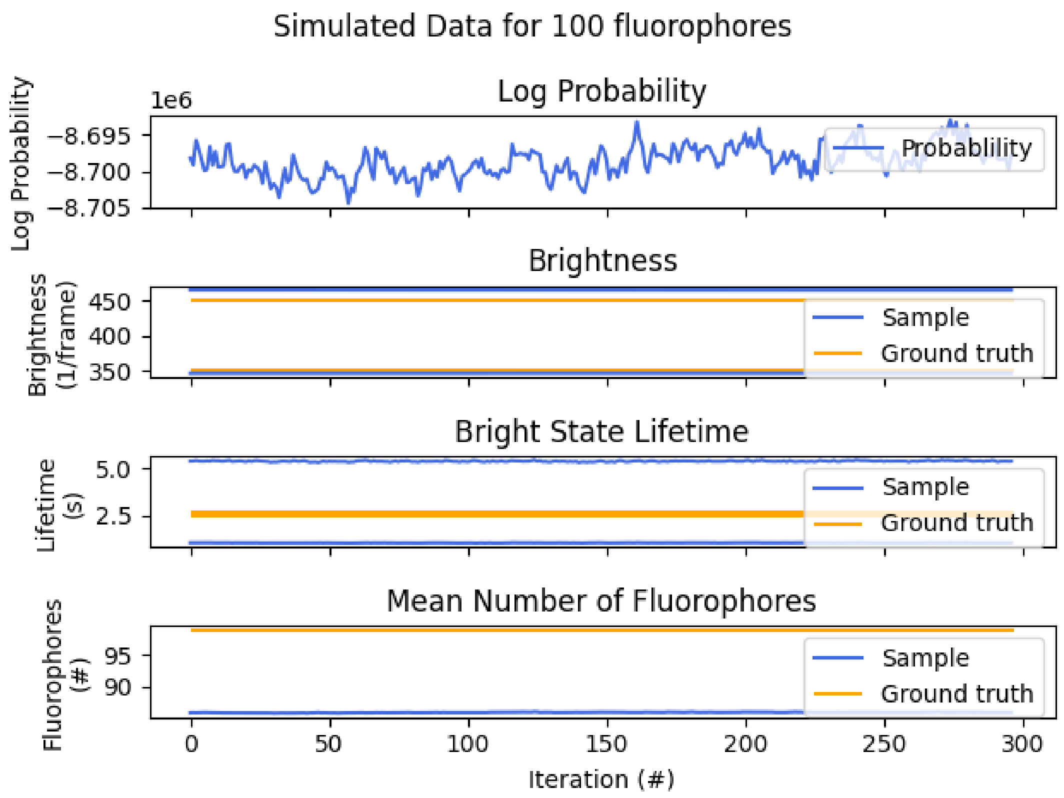

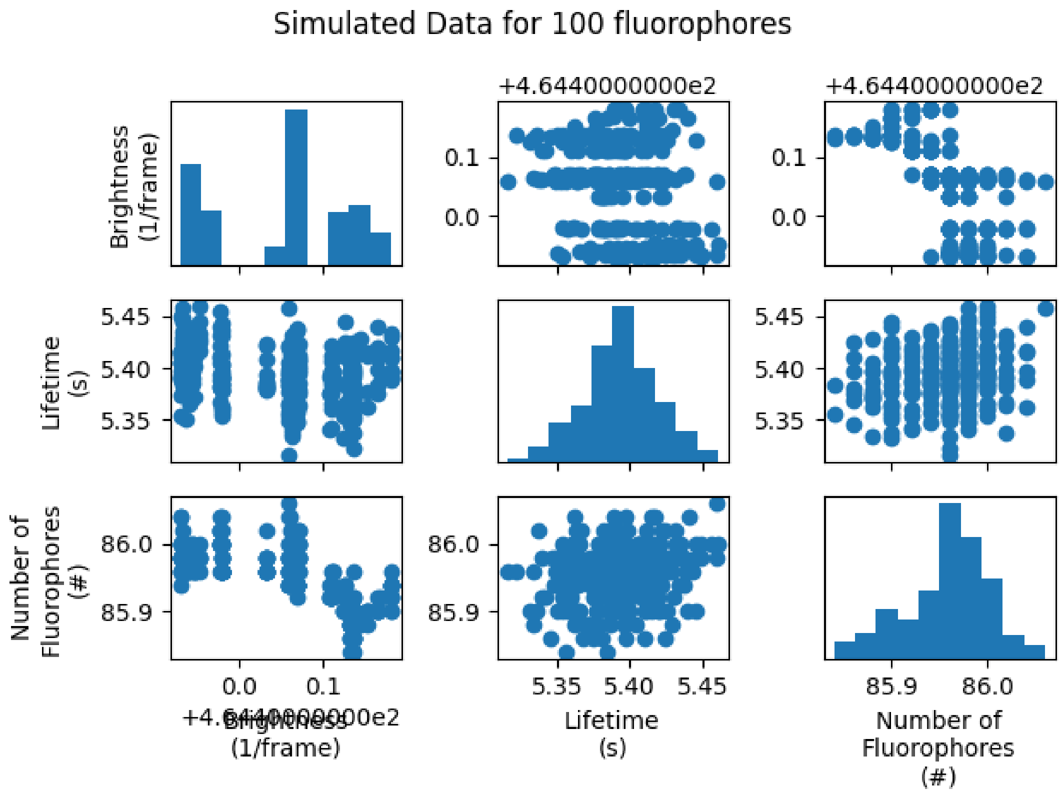

bioRxiv preprint doi: https://doi.org/10.1101/2020.09.28.317057; this version posted September 23, 2021. The copyright holder for this preprint (which was not certified by peer review) is the author/funder, who has granted bioRxiv a license to display the preprint in perpetuity. It is made available under aCC-BY 4.0 International license.

5.13 Plots of data

Here we plot the brightness vs time traces that we analyze in the main manuscript. Note that we only plot data from the first three ROIs, but there are, in fact, many more traces that we do not show. On the top under the title of each figure we print the total number of ROIs per data set.

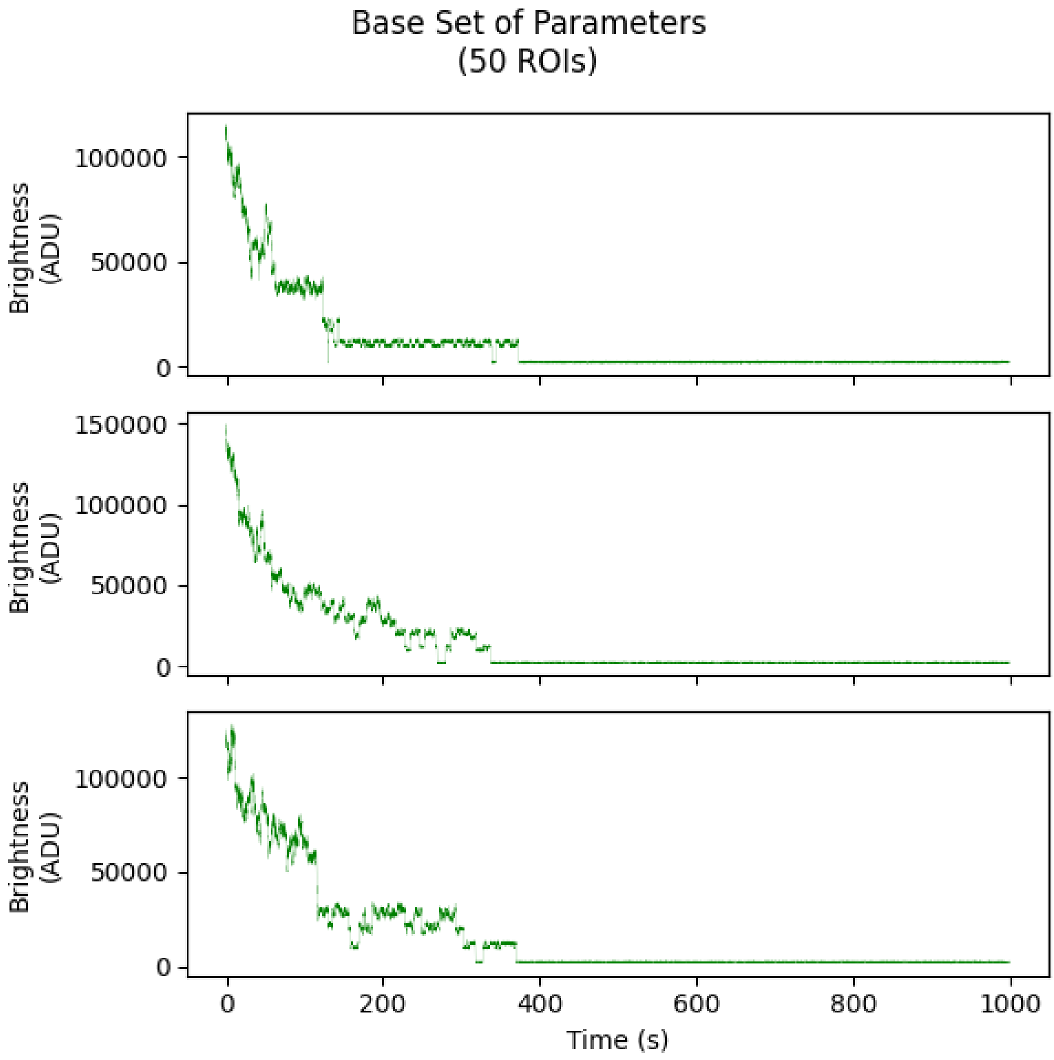

bioRxiv preprint doi: https://doi.org/10.1101/2020.09.28.317057; this version posted September 23, 2021. The copyright holder for this preprint (which was not certified by peer review) is the author/funder, who has granted bioRxiv a license to display the preprint in perpetuity. It is made available under aCC-BY 4.0 International license.

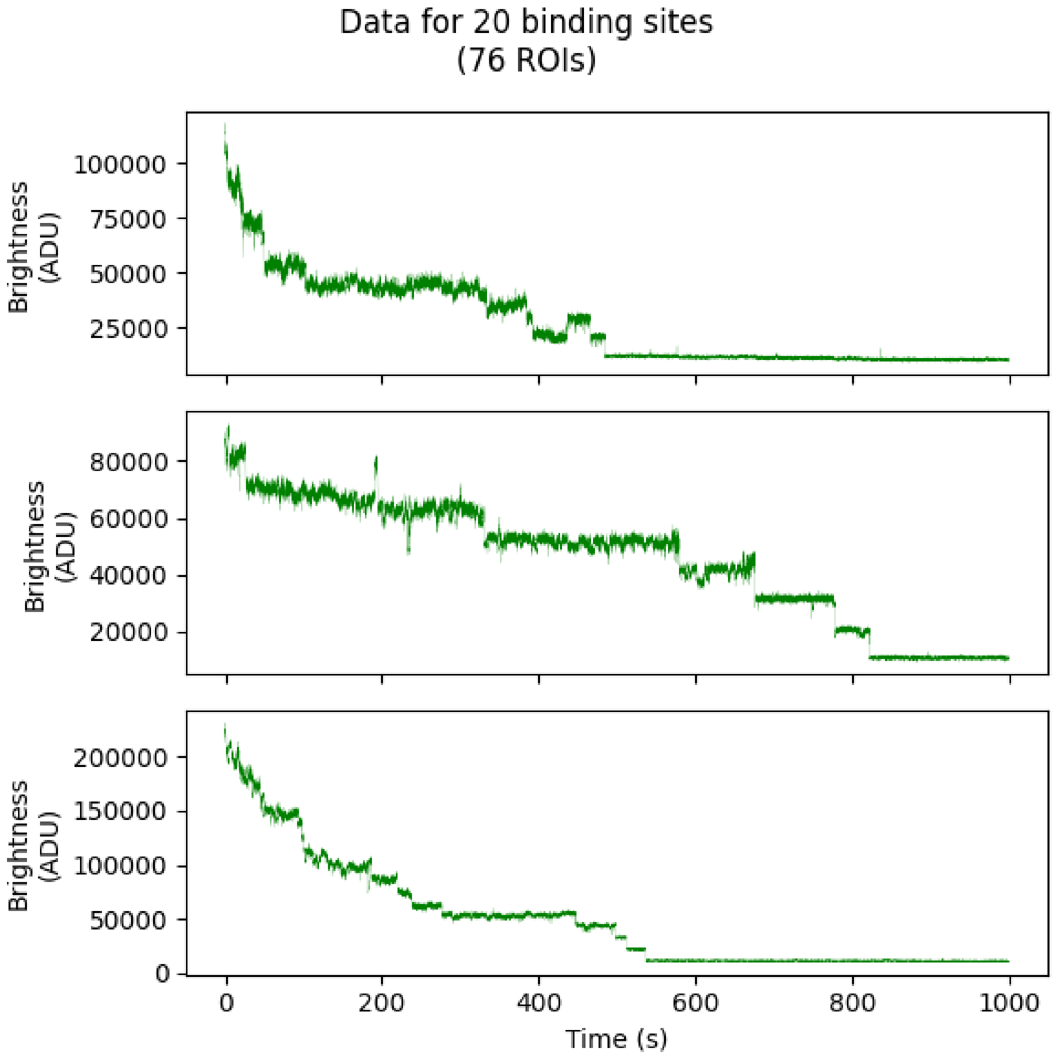

bioRxiv preprint doi: https://doi.org/10.1101/2020.09.28.317057; this version posted September 23, 2021. The copyright holder for this preprint (which was not certified by peer review) is the author/funder, who has granted bioRxiv a license to display the preprint in perpetuity. It is made available under aCC-BY 4.0 International license.

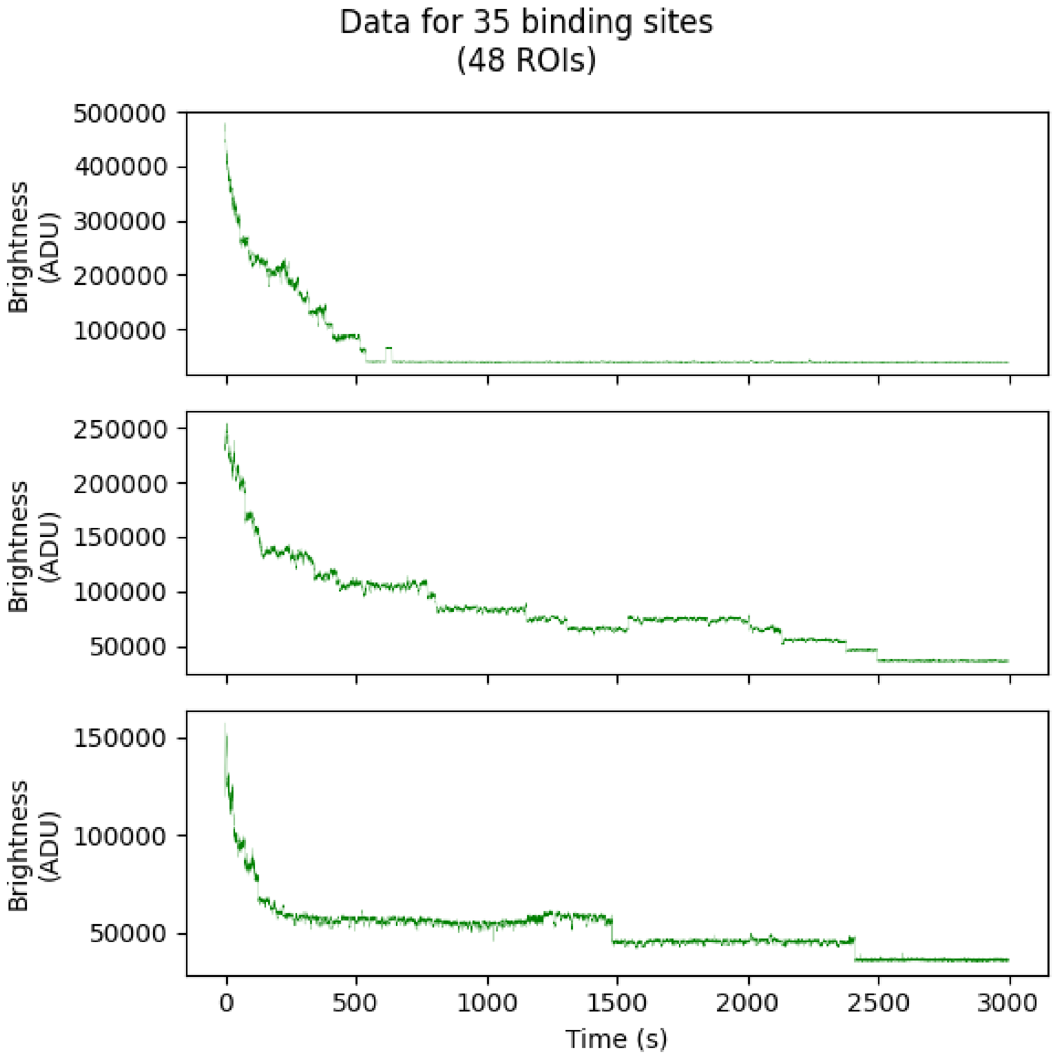

bioRxiv preprint doi: https://doi.org/10.1101/2020.09.28.317057; this version posted September 23, 2021. The copyright holder for this preprint (which was not certified by peer review) is the author/funder, who has granted bioRxiv a license to display the preprint in perpetuity. It is made available under aCC-BY 4.0 International license.

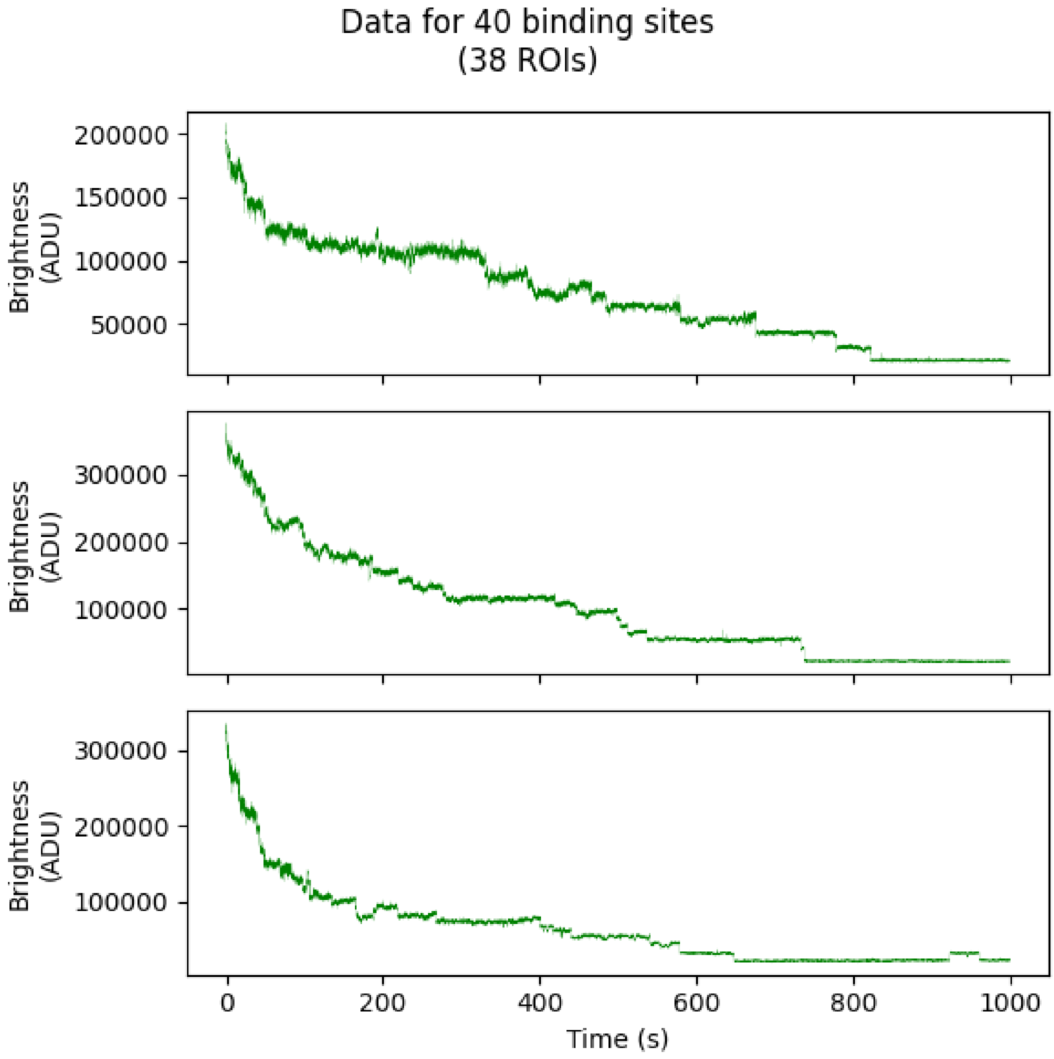

bioRxiv preprint doi: https://doi.org/10.1101/2020.09.28.317057; this version posted September 23, 2021. The copyright holder for this preprint (which was not certified by peer review) is the author/funder, who has granted bioRxiv a license to display the preprint in perpetuity. It is made available under aCC-BY 4.0 International license.

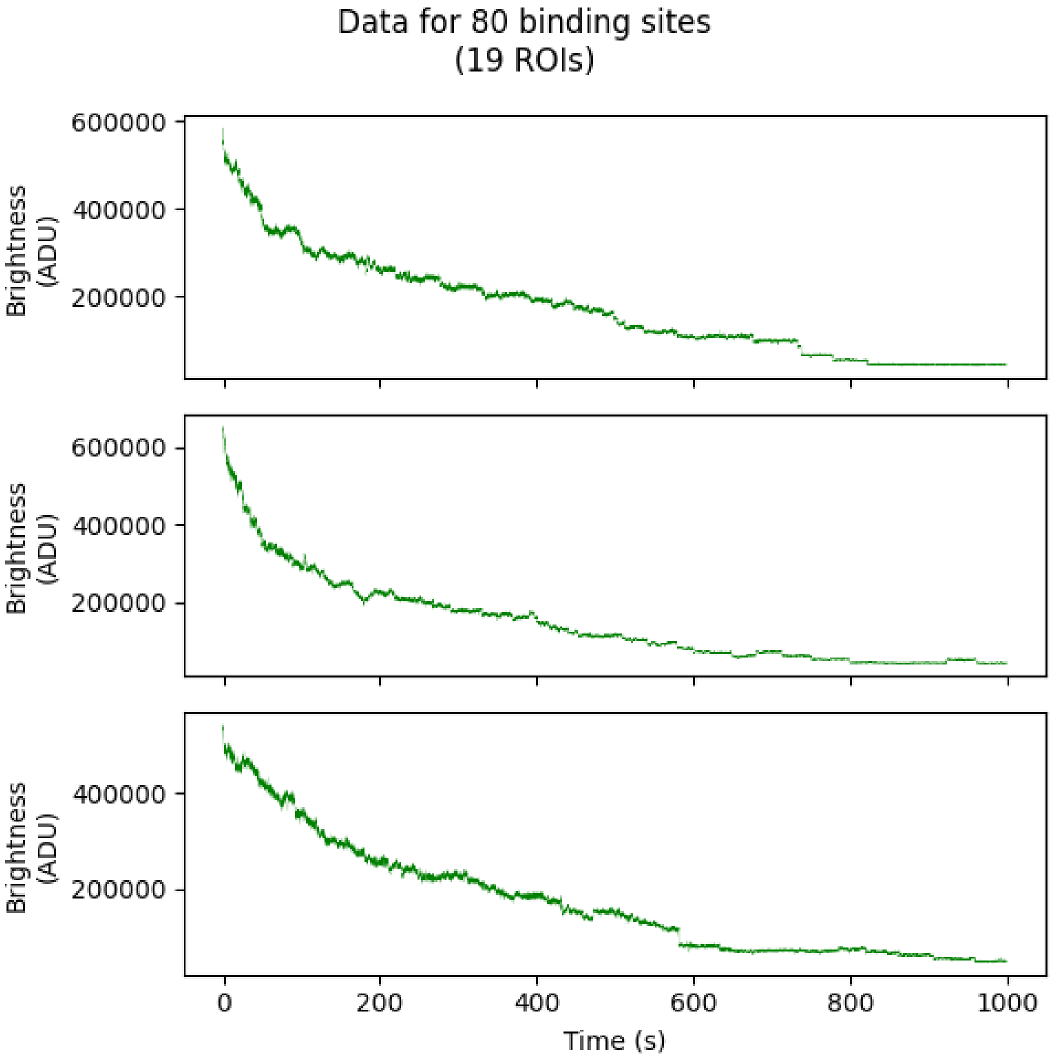

bioRxiv preprint doi: https://doi.org/10.1101/2020.09.28.317057; this version posted September 23, 2021. The copyright holder for this preprint (which was not certified by peer review) is the author/funder, who has granted bioRxiv a license to display the preprint in perpetuity. It is made available under aCC-BY 4.0 International license.

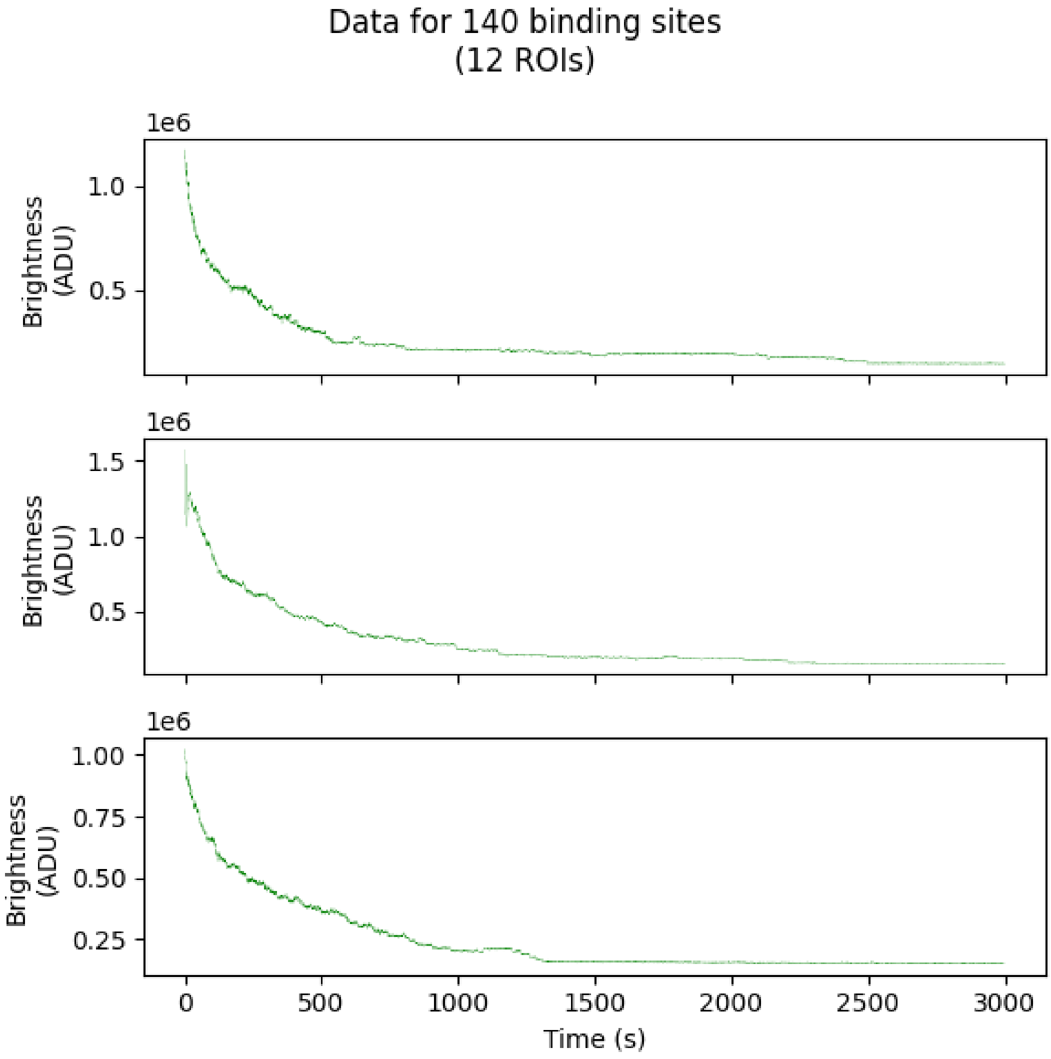

bioRxiv preprint doi: https://doi.org/10.1101/2020.09.28.317057; this version posted September 23, 2021. The copyright holder for this preprint (which was not certified by peer review) is the author/funder, who has granted bioRxiv a license to display the preprint in perpetuity. It is made available under aCC-BY 4.0 International license.

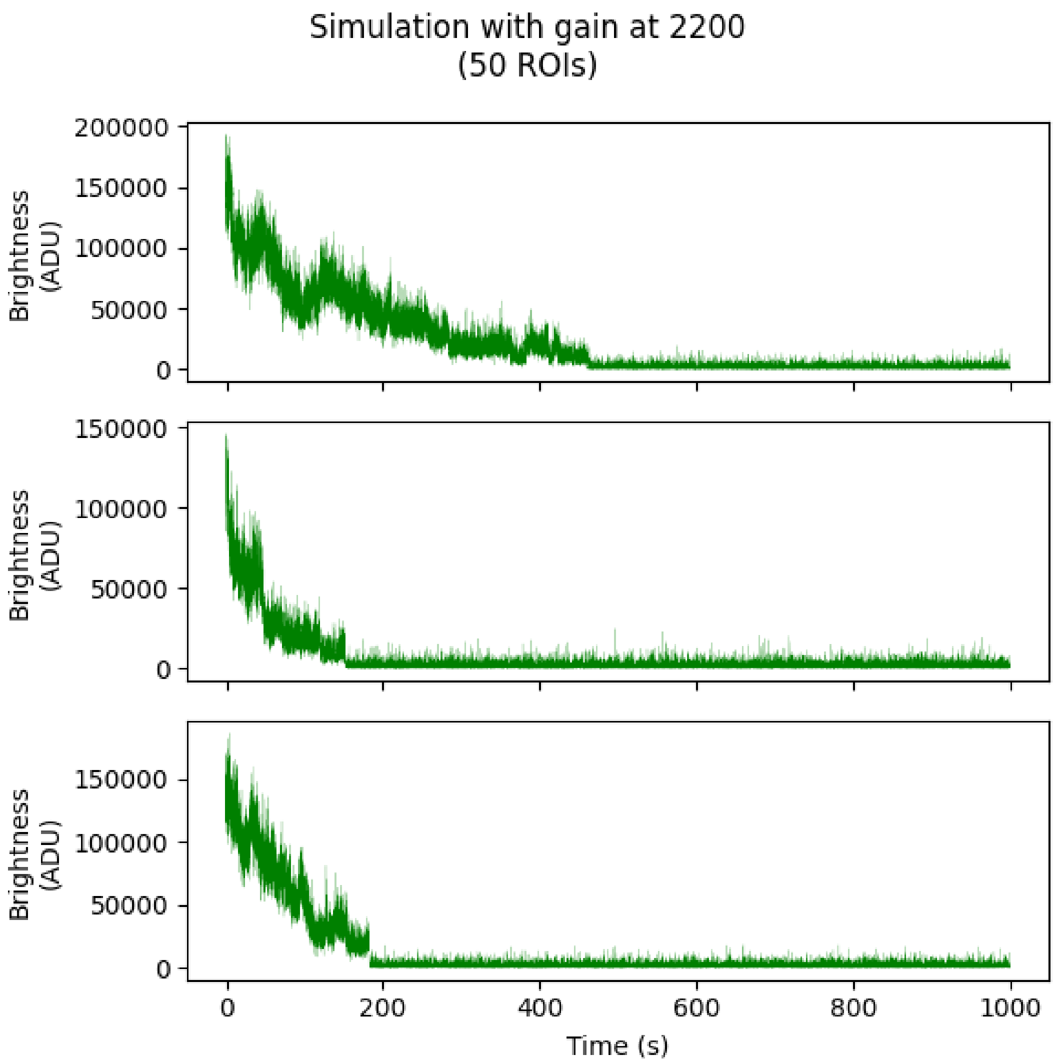

bioRxiv preprint doi: https://doi.org/10.1101/2020.09.28.317057; this version posted September 23, 2021. The copyright holder for this preprint (which was not certified by peer review) is the author/funder, who has granted bioRxiv a license to display the preprint in perpetuity. It is made available under aCC-BY 4.0 International license.

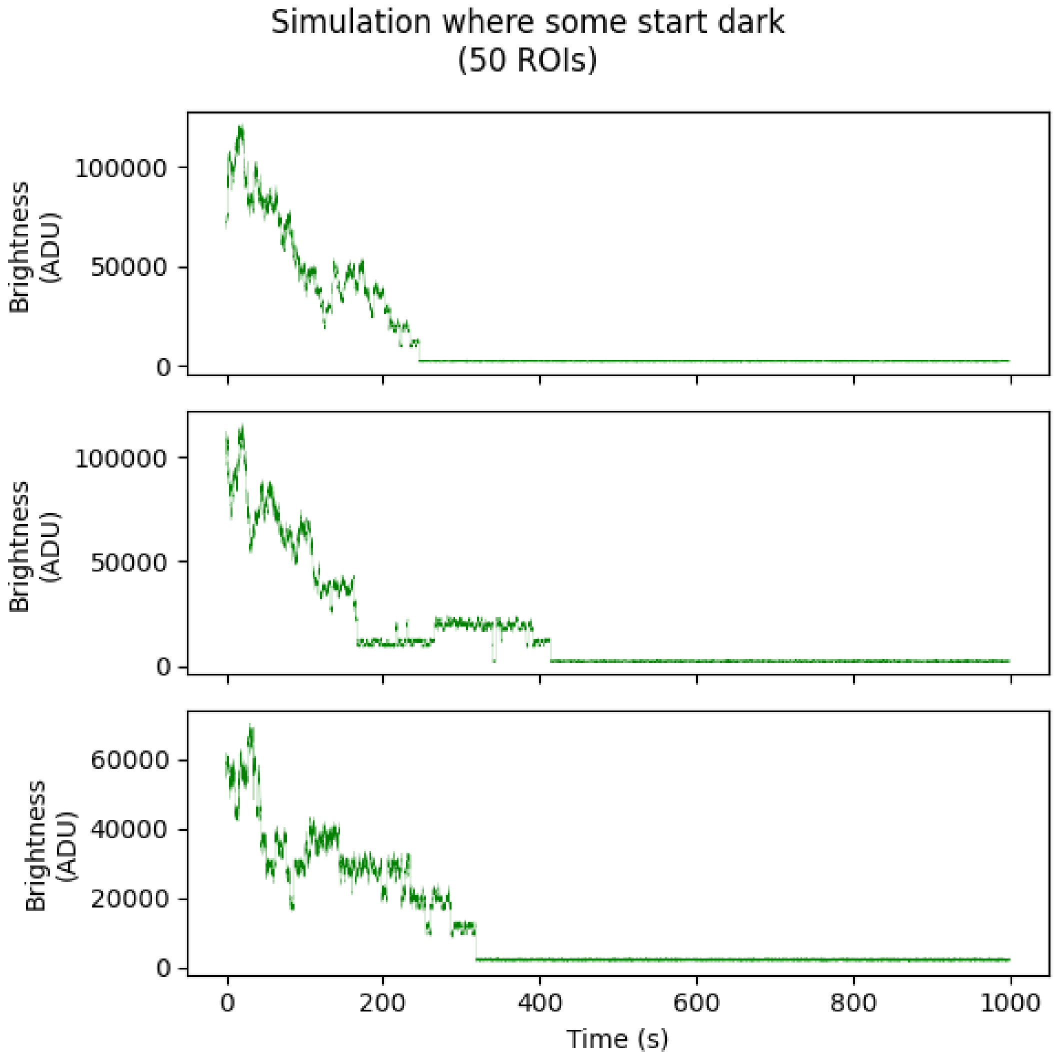

bioRxiv preprint doi: https://doi.org/10.1101/2020.09.28.317057; this version posted September 23, 2021. The copyright holder for this preprint (which was not certified by peer review) is the author/funder, who has granted bioRxiv a license to display the preprint in perpetuity. It is made available under aCC-BY 4.0 International license.

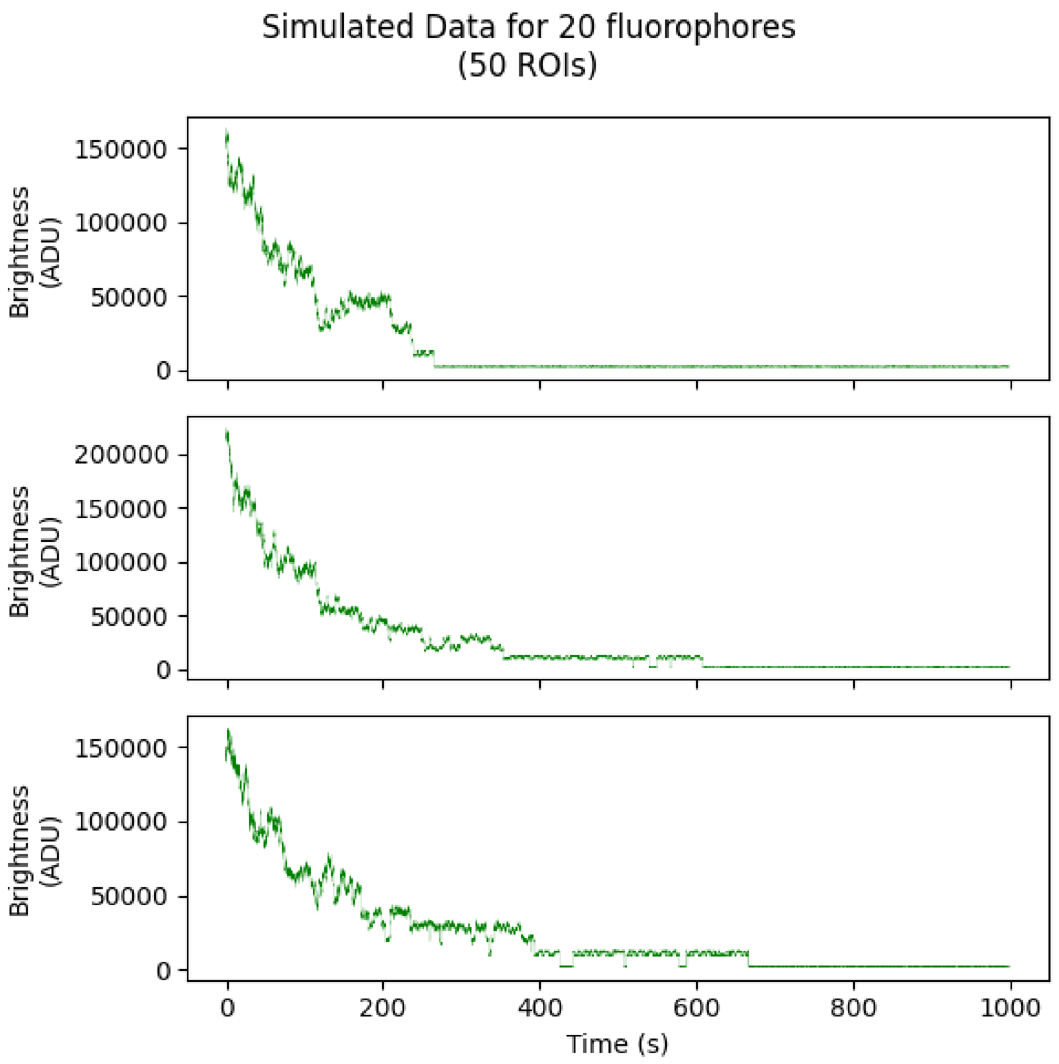

bioRxiv preprint doi: https://doi.org/10.1101/2020.09.28.317057; this version posted September 23, 2021. The copyright holder for this preprint (which was not certified by peer review) is the author/funder, who has granted bioRxiv a license to display the preprint in perpetuity. It is made available under aCC-BY 4.0 International license.

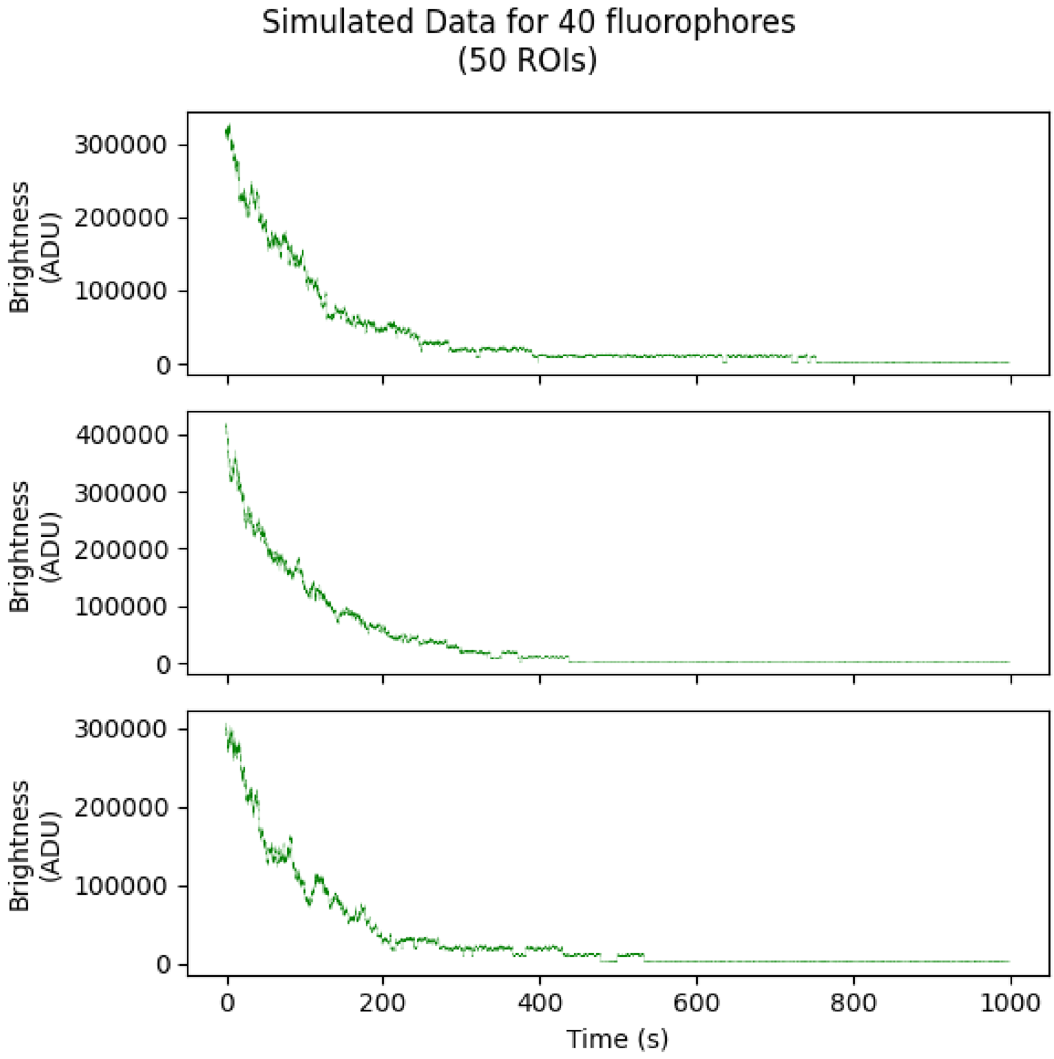

bioRxiv preprint doi: https://doi.org/10.1101/2020.09.28.317057; this version posted September 23, 2021. The copyright holder for this preprint (which was not certified by peer review) is the author/funder, who has granted bioRxiv a license to display the preprint in perpetuity. It is made available under aCC-BY 4.0 International license.

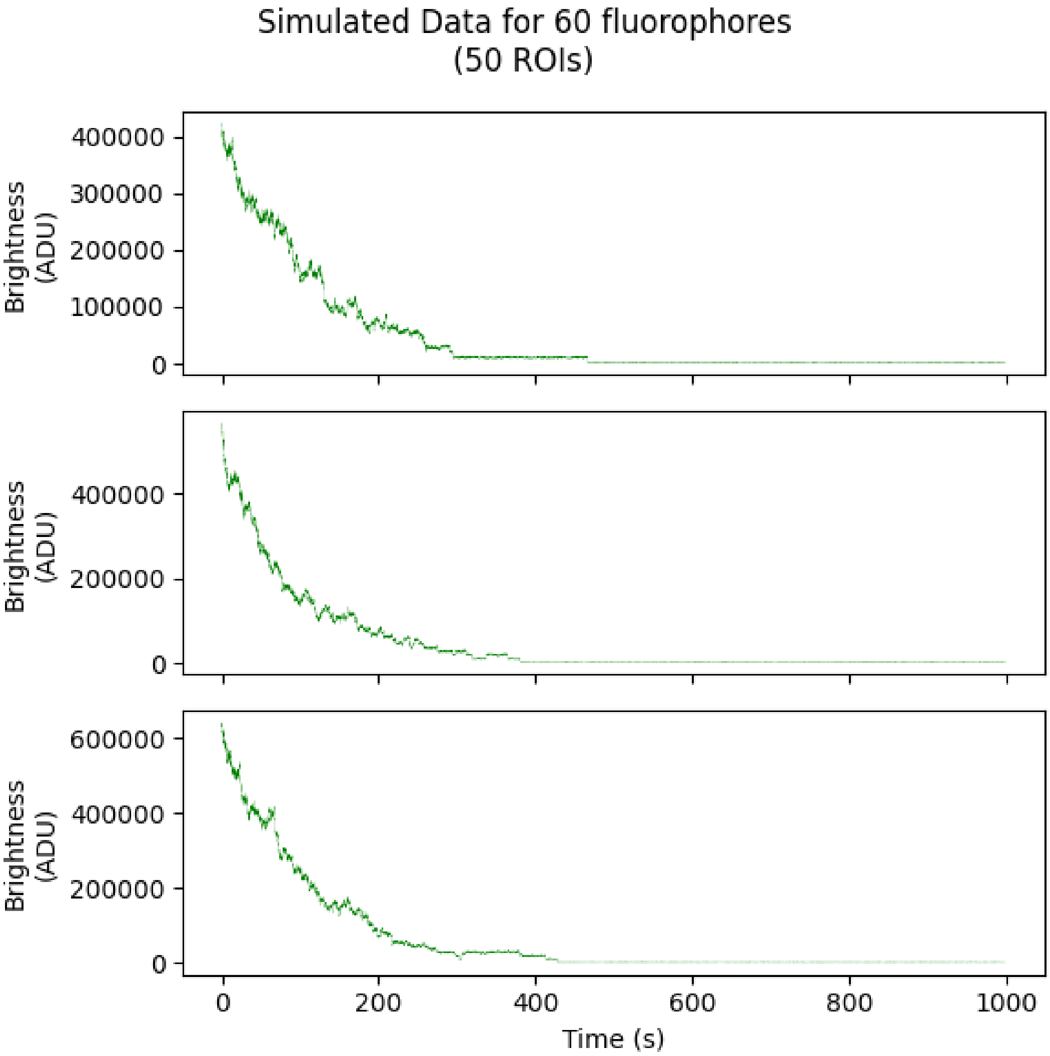

bioRxiv preprint doi: https://doi.org/10.1101/2020.09.28.317057; this version posted September 23, 2021. The copyright holder for this preprint (which was not certified by peer review) is the author/funder, who has granted bioRxiv a license to display the preprint in perpetuity. It is made available under aCC-BY 4.0 International license.

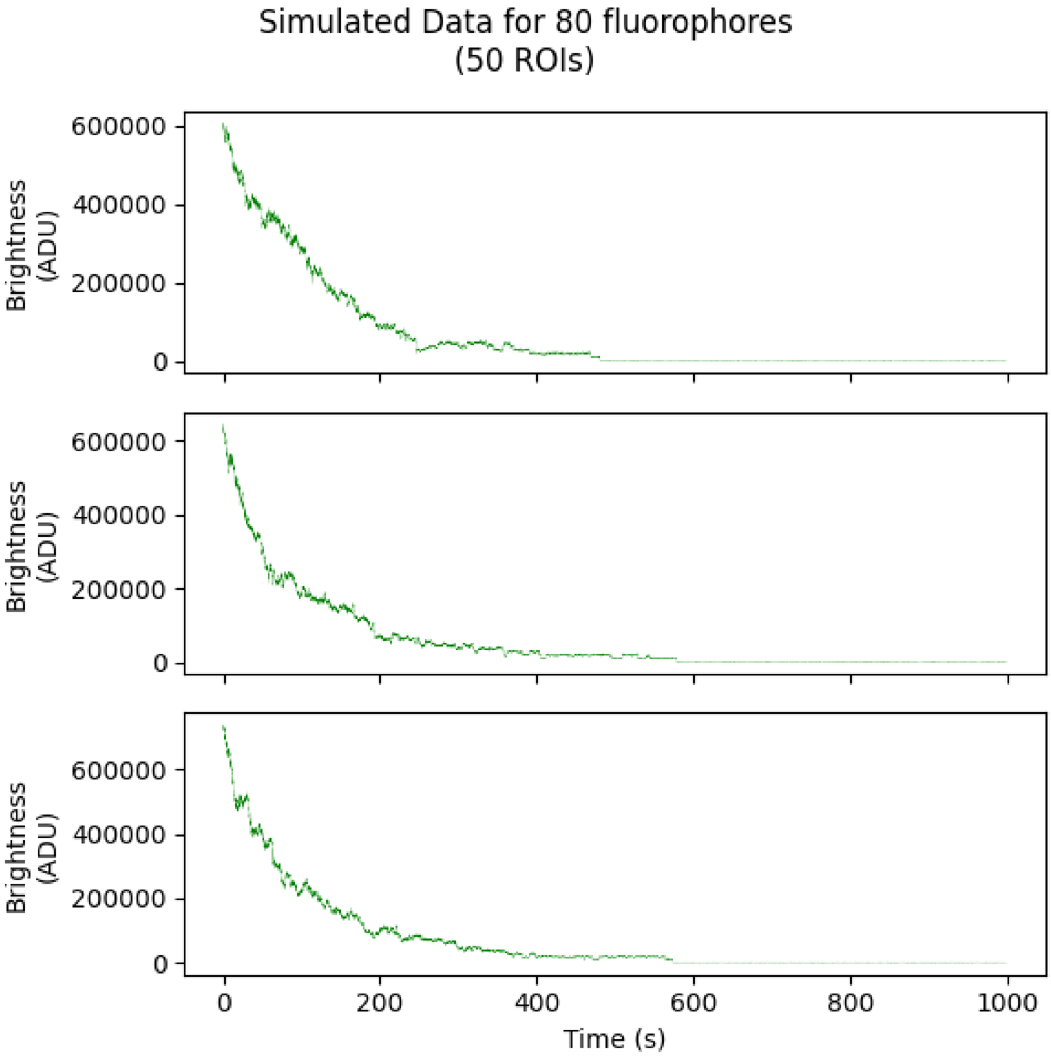

bioRxiv preprint doi: https://doi.org/10.1101/2020.09.28.317057; this version posted September 23, 2021. The copyright holder for this preprint (which was not certified by peer review) is the author/funder, who has granted bioRxiv a license to display the preprint in perpetuity. It is made available under aCC-BY 4.0 International license.

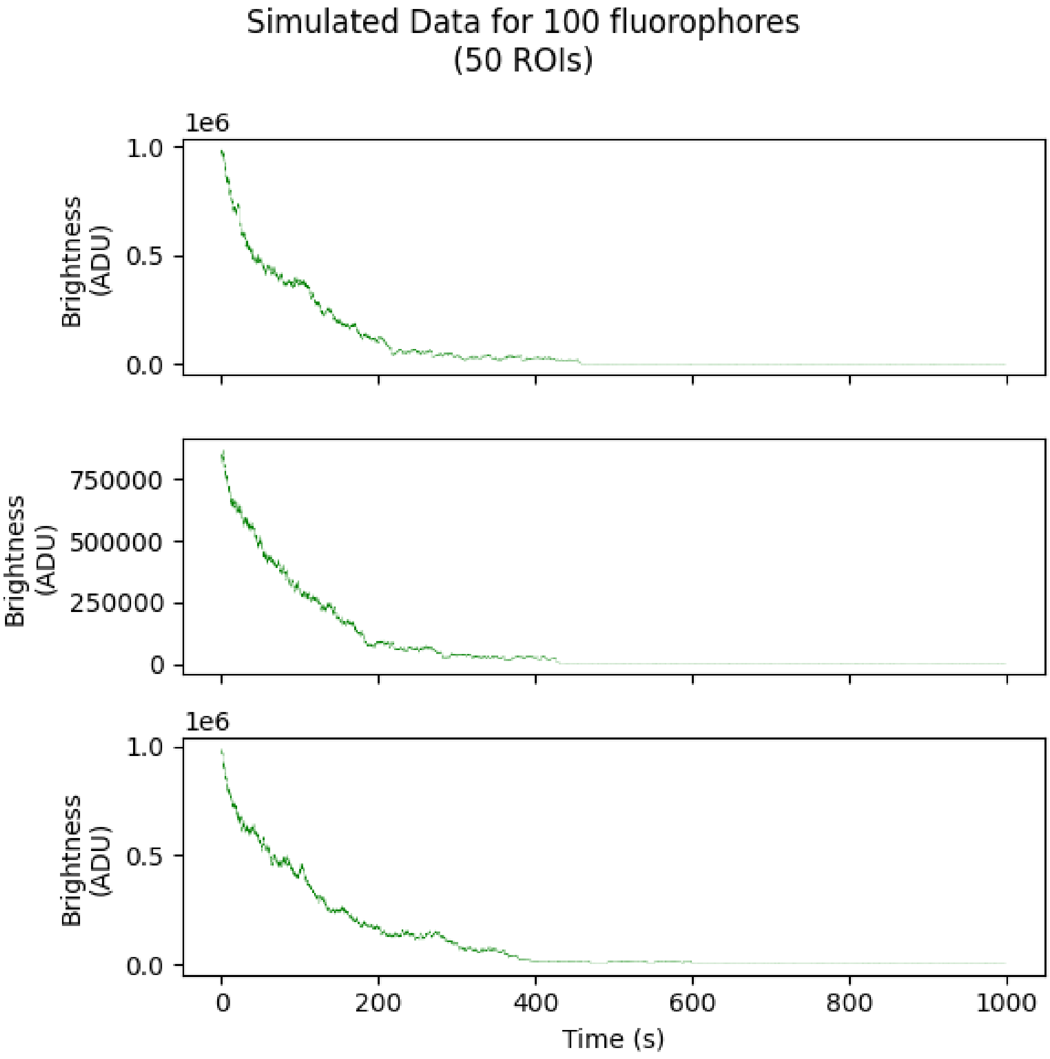

Supplement: 1 [file NIHMS1823309-supplement-1.pdf]
